# Rapid Synthesis of *anti*‐1,3‐Diamino‐4‐phenylbutan‐2‐ol Building Blocks via a Three‐Component Oxyhomologation and a Two‐Component Reducing System

**DOI:** 10.1002/open.202400279

**Published:** 2024-10-30

**Authors:** Maria Chiara Cabua, Xuefeng He, Francesco Secci, Sandrine Deloisy, David J. Aitken

**Affiliations:** ^1^ CNRS ICMMO CP3A Organic Synthesis Group Université Paris-Saclay 17 Avenue des Sciences 91400 Orsay France; ^2^ Department of Chemical and Geological Science University of Cagliari S.P. No. 8 Km 0.700 09042 Monserrato Italy

**Keywords:** Multi-component reactions, HEA drugs, Aminoalcohols, Stereoselectivity, Homologation

## Abstract

*N* ^1^‐substituted derivatives of *anti*‐(2*R*,3*S*)‐1,3‐diamino‐4‐phenylbutan‐2‐ol are important building blocks for the synthesis of therapeutically important molecules. We describe a simple protocol that allows transformation of *N,N*‐dibenzyl‐L‐phenylalaninal into such compounds in only two steps. The first step is a fully stereoselective three‐component MAC (Masked Acyl Cyanide) oxyhomologation reaction implicating different amines to give a panel of ten *N,N*‐dibenzyl‐*O*‐*tert*‐butyldimethylsilyl‐protected *anti*‐(2*S*,3*S*)‐allophenylnorstatin amides. The second step is a carbonyl‐activated hydride deprotection/reduction protocol using trimethylsilyl chloride and lithium aluminium hydride; the one‐pot two‐component system is more efficient than the alternative approach of isolating the deprotected amide intermediate before reduction.

## Graphical abstract

*N* ^1^‐substituted derivatives of *anti‐(*2*R*,3*S)*‐1,3‐diamino‐4‐phenylbutan‐2‐ol are highly important building blocks in medicinal chemistry. This paper describes a remarkably simple, fully stereoselective, two‐step access to a representative panel of these compounds in *N* ^3^‐dibenzyl‐protected form.

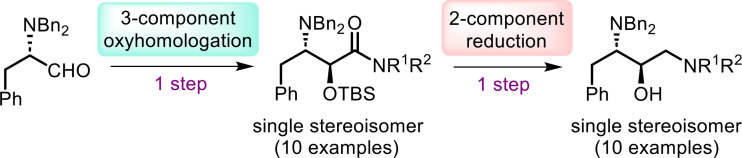

## Introduction

**Figure 1:**
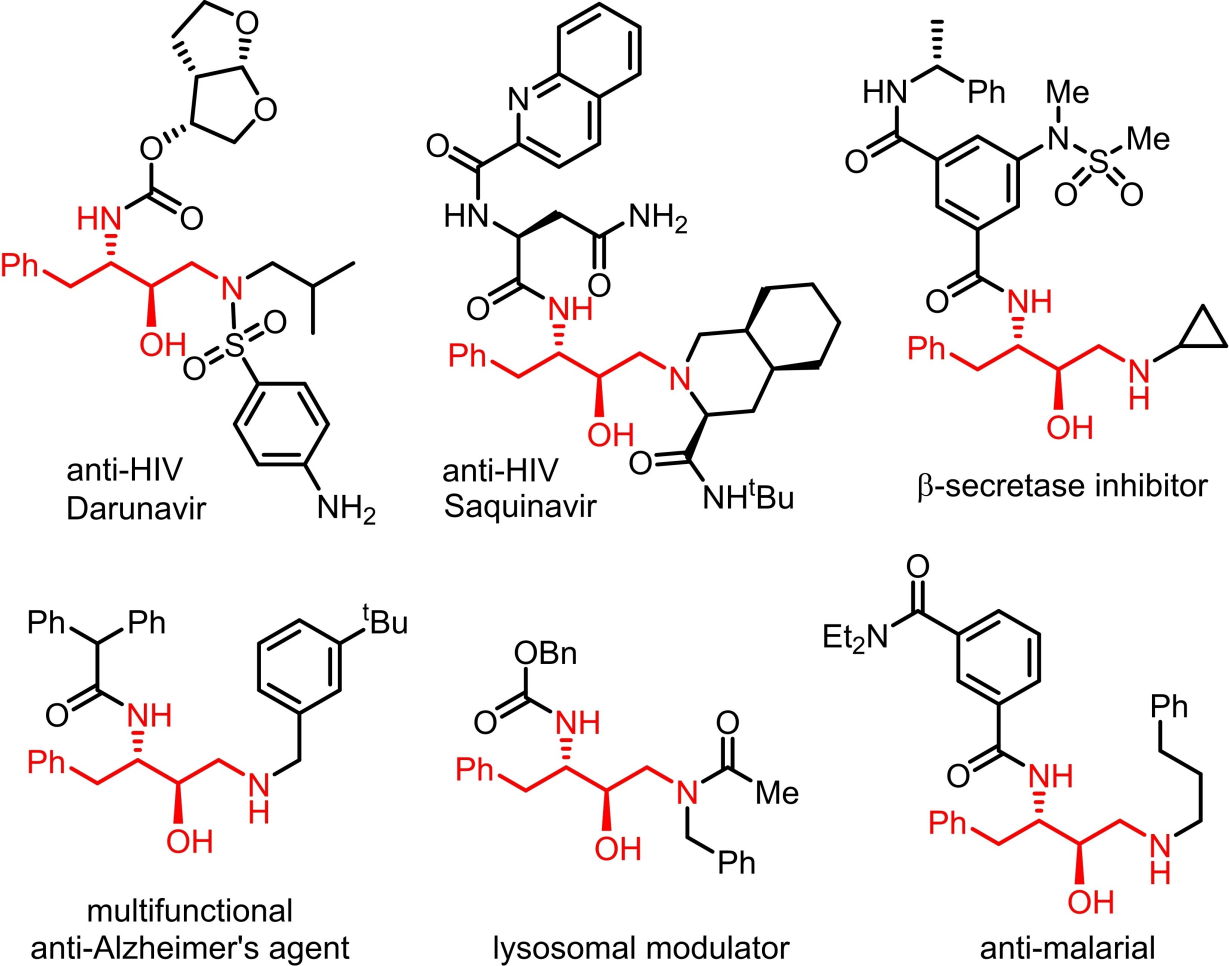
Examples of biologically active molecules that incorporate an *anti*‐(2*R*,3*S*) DAPB unit (highlighted in red).

1,3‐Diamino‐4‐phenylbutan‐2‐ol (DAPB) derivatives are highly important molecular building blocks in biological and medicinal chemistry, appearing as core structural motifs in a wide range of therapeutically important molecules (sometimes referred to as hydroxyethylamine drugs or HEA drugs). In numerous cases, the *N* ^3^ nitrogen is part of an amide or carbamate function, while the *N* ^1^ nitrogen is alkylated and may also be part of an amide function. Many such compounds are potent inhibitors of HIV protease – a key target in HIV therapy^[1]^ – as exemplified by the drugs Darunavir^[2]^ and Saquinavir,^[3]^ while others show activities as diverse as β‐secretase inhibitors,^[4]^ anti‐malarials,^[5]^ lysosomal modulators^[6]^ and multifunctional anti‐Alzheimer's agents^[7]^ (Figure 1). An important common factor in all the molecules is the *anti*‐(2*R*,3*S*) configuration of the DAPB unit.

**Figure 2:**
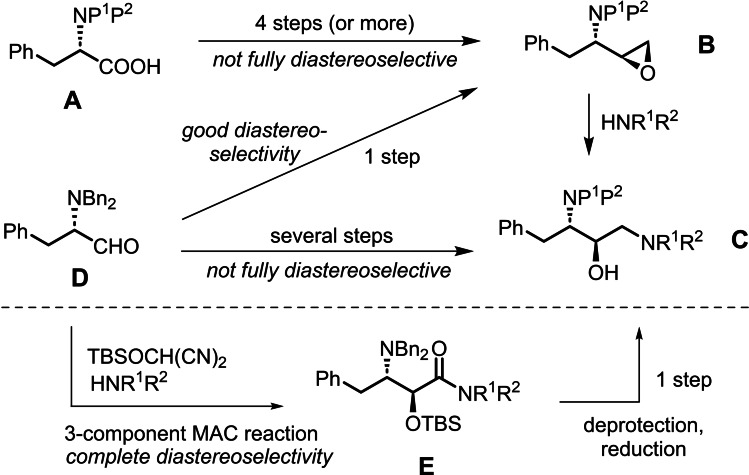
Synthetic routes to *anti*‐(2*R*,3*S*) DAPB building blocks, summarizing known strategies (upper) and the present work (lower).

Various synthetic strategies have been established for the configuration‐controlled preparation of *anti*‐(2*R*,3*S*) DAPB building blocks and the most common practice has been to use an *N*‐protected derivative of L‐phenylalanine **A** as the starting material (Figure 2, upper). Frequently, the strategy implicates an *N*‐protected epoxide intermediate **B**, which reacts with an amine in a regioselective manner to give the target *N* ^1^‐alkylated DAPB structure **C**.^[8]^ However, the syntheses of such epoxides, typically with P^1^=H and P^2^=either *t*‐butyl carbamate (Boc) or benzyl carbamate (Z), require multi‐step protocols that are not always diastereoselective.[^9^, ^10^] As a useful alternative, the *N,N*‐dibenzyl protected L‐phenylalaninal **D** has been used to prepare the corresponding epoxide **B** (P^1^, P^2^=Bn) in one step and with high diastereoselectivity,[^11^, ^12^] and an adaptation of the procedure allowed access to epoxide **B** (P^1^=H and P^2^=Boc) on large scale.^[13]^ Other one‐carbon homologation strategies starting from **D** ‐ via a cyanohydrin[^6^, ^14^] or a nitroaldol (Henry reaction)^[15]^ ‐ have been employed but require several steps to attain **C** and remain limited in scope. Alternative strategies starting from L‐Phe^[16]^ or from other compounds^[17]^ have been reported on only rare occasions.

The three‐component reaction that combines (i) an electrophile (usually an aldehyde), (ii) a nucleophile (an alcohol or an amine), and (iii) the *tert*‐butyldimethylsilyl ether of hydroxymalononitrile (TBSOCH(CN)_2_, named H‐MAC‐TBS) constitutes the oxyhomologation of the aldehyde and is a key feature of MAC (Masked Acyl Cyanide) methodology.[^18^, ^19^, ^20^] We recently discovered that when the electrophilic partner was *N,N*‐dibenzyl‐ L‐phenylalaninal **D** and the nucleophile was an amine, the MAC oxyhomologation reaction provided a single‐step access to (2*S*,3*S*)‐allophenylnorstatin amides **E** with very high *anti* selectivity (dr>98 : 2).^[18a]^ It seemed to us that it should be possible to prepare a panel of such amides and transform them into the corresponding *anti*‐(2*R*,3*S*) DAPBs, thus providing a very short (two‐step) synthesis of these target building blocks (Figure 2, lower). In this paper we describe the accomplishment of this objective.

## Results and Discussion

**Scheme 1:**
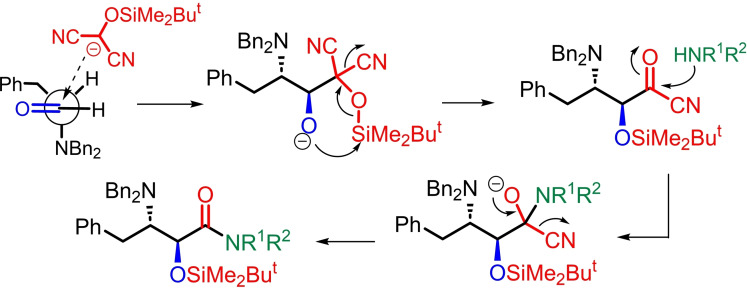
Mechanistic proposal for the formation of carboxamides **1 a**–**j** with an *anti* configuration.

We used the three‐component MAC reaction to prepare the secondary and tertiary amide derivatives **1 a**–**j**, starting from *N,N*‐dibenzyl‐L‐phenylalaninal as shown in Table 1. The reactions were carried out in Et_2_O using a previously‐optimized protocol, employing 4‐(dimethylamino)pyridine (DMAP) as a weak base in mild conditions.^[18a]^ The yields of isolated material were entirely satisfying (63–87 %), regardless of the identity of the nucleophilic amine component. In all cases the *anti* diastereoselectivity was >98 : 2, confirmed by ^1^H NMR spectra. The absolute configuration of compound **1 c** was confirmed by an X‐ray diffraction study of a single crystal (see SI).^[21]^ It is proposed that the origin of the *anti* diastereoselectivity is in the first step of the MAC reaction, whereby the deprotonated form of H‐MAC‐TBS adds to the aldehyde via a Felkin‐Anh model (Scheme 1), followed by a 1,4‐silyl transfer and elimination of cyanide to furnish an *anti* acyl cyanide, that reacts with the amine nucleophile in the final step.

**Table 1:** Three‐component MAC reactions for the preparation of *anti* carboxamides **1 a**–**j** from *N,N*‐dibenzyl‐L‐phenylalaninal.

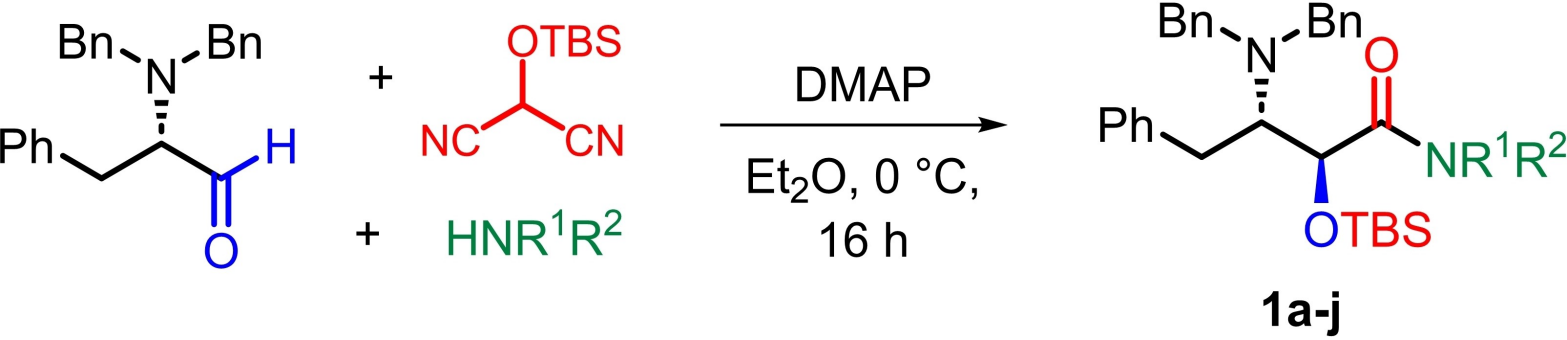		
Compound label	R^1^, R^2^	Yield (%) **1 a**–**j**
a	H, iBu	76
b	H, nBu	76
c	H, (CH_2_)_3_Ph	69
d	H, Bn	70
e	H, allyl	71
f	H, propargyl	72
g	H, iPr	7.9
h	H, *c*C_3_H_5_	87
i	−C_2_H_4_OC_2_H_4_−	69
j	−(CH_2_)_4_−	63

Reactions were carried out on 0.5 mmol scale using 2.4 equiv. H‐MAC‐TBS, 1.2 equiv. HNR^1^R^2^ and 2 equiv. DMAP. Isolated yields are indicated.

To determine appropriate reduction conditions for amides **1 a**–**j** we used compound **1 a** as a representative substrate. The results of studies of its transformation into the target diamine **3 a** are summarized in Table 2. Lithium aluminium hydride (LAH) is a well‐established reducing agent for carboxamides^[22]^ and is also known to deprotect silyl ethers.^[23]^ However, prolonged treatment of **1 a** with LAH in refluxing tetrahydrofuran (THF) gave, after standard work‐up and column chromatography, only a poor yield of **3 a** (21 %), accompanied by a similar amount of the alcohol‐deprotected carboxamide intermediate **2 a** (entry 1). Reduction of secondary and tertiary amides with LAH is reported to be facilitated by activation of the carbonyl group with trimethylsilyl chloride (TMSCl).^[24]^ A THF solution of **1 a** at 0 °C was treated with a slight excess of TMSCl (1.2 equiv.) for 15 min, followed by LAH (2.4 equiv.). After 2 h reaction, **3 a** was obtained in a marginally improved yield (26 %), accompanied by larger amounts of **2 a** (entry 2). When this procedure was repeated using CH_2_Cl_2_ as the solvent instead of THF, very little **2 a** remained after 5 h and the isolated yield of **3 a** improved to 41 % (entry 3). We found that by using less LAH (1.4 equiv.) and a slightly shorter reaction time (3 h), the yield of **3 a** reached 53 % (entry 4). Finally, by limiting the reaction time to 1.5 h, we obtained **3 a** in a very satisfactory 80 % yield (entry 5).

**Table 2:** Optimization of the one‐pot deprotection and reduction of substrate **1 a**.

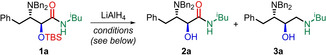				
Entry^[a]^	Solvent	Time (h)	Ratio **2 a** : **3 a** ^[b]^	Yield **3 a** (%)
1^[c]^	THF	32	1 : 1	21
2^[d]^	THF	2	2 : 1	26
3^[d].^	CH_2_Cl_2_	5	1 : 4	41
4	CH_2_Cl_2_	3	1 : 6	53
5	CH_2_Cl_2_	1.5	1 : 7	80

[a] Reactions were carried out at 0 °C using 1.2 equiv. TMSCl followed by 1.4 equiv. LAH, unless otherwise indicated. Isolated yields of **3 a** are indicated. [b] Assessed by inspection of the ^1^H N.MR spectra of crude reaction products. [c] TMSCl was not added; 4 equiv. LAH were used; reaction performed at reflux. [d] 2.4 equiv. LAH were used.

**Scheme 2:**
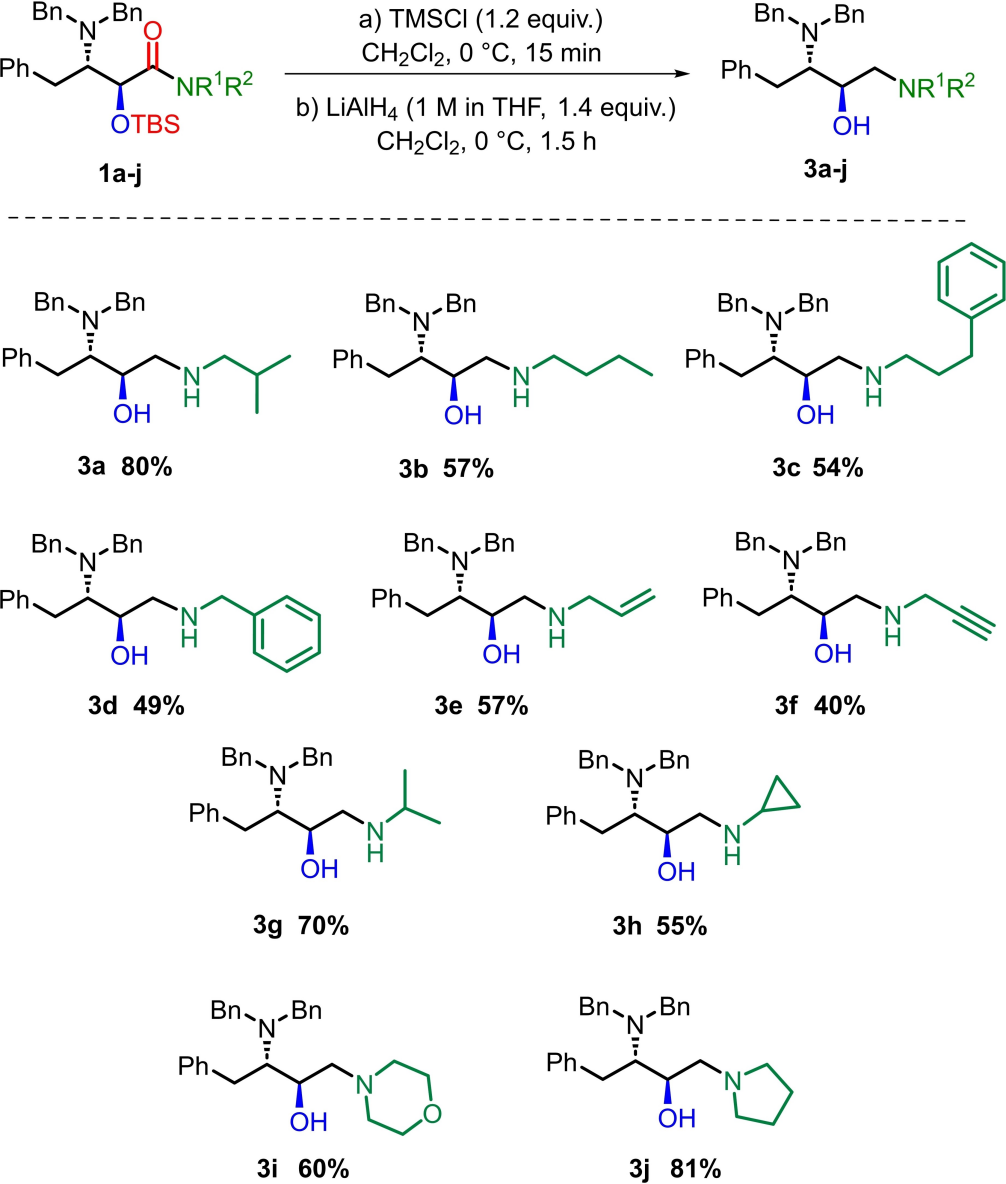
Preparation of *anti*‐(2*R*,3*S*) DAPB building blocks using the two‐component reducing system.

With the optimized one‐pot reduction conditions in hand, we applied them to the full set of ten amide derivatives **1 a**–**j**. The results are presented in Scheme 2 and include the success for *N*‐isobutyl amine **3 a**. The *N*‐butyl, *N*‐3‐phenylpropyl, *N*‐benzyl and *N*‐allyl amines **3 b**–**e** were all isolated in satisfactory yields (49–57 %), while the *N*‐propargyl amine **3 f** was obtained in slightly lower yield (40 %), accompanied by the intermediate **2 f** (21 %). C^α^‐branched substituents were suitably accommodated in the form of *N*‐isopropyl and *N*‐cyclopropyl amines **3 g** and **3 h**, obtained in yields of 70 % and 55 %, respectively. The tertiary amides **1 i** and **1 j** performed well as substrates for the one‐pot transformation, with **3 i** and **3 j** being obtained in 60 % and 81 % yields, respectively. All of the above‐described compounds **3 a**–**j** were obtained as single stereoisomers with no erosion of the *anti* configuration, as testified by their ^1^H NMR spectra. The success of this protocol is noteworthy in that there are few examples in the literature of a LAH reduction of a carboxamide with a stereogenic center α to the carbonyl group into the corresponding amines;[^24^, ^25^] indeed, epimerization was reported on one occasion.^[25b]^

For comparison, we examined in parallel the two‐step sequence. Results are presented in Table 3. For the first step, a solution of the substrate **1 a**–**j** in THF at 0 °C was treated with a slight excess (1.5 equiv.) of tetrabutylammonium fluoride (TBAF) for 1 h, using a protocol that had previously proved successful for the selective deprotection of *O*‐TBS silyl ether derivatives of (2*S*,3*S*)‐allophenylnorstatin esters.^[18c]^ After work‐up and chromatography, the corresponding *anti* alcohols **2 a**–**j** were isolated in uniformly high yields (79–92 %) and ^1^H NMR analysis of each compound indicated that only one diastereoisomer was present. None of these compounds has been described before in the literature. The second step was conducted using the conditions that were optimal for the one‐pot protocol above: a solution of **2 a**–**j** in CH_2_Cl_2_ at 0 °C was treated with TMSCl (1.2 equiv.) for 15 min, followed by LAH (1.4 equiv.), and a reaction time of 1.5 h. These transformations turned out to be less efficient than had been observed in the one‐pot procedure: product mixtures were obtained in which starting materials **2 a**–**j** were still present, with the **2 a**–**j**:**3 a**–**j** ratio varying marginally between 2 : 3 and 3 : 2. From these mixtures it was possible to isolate by chromatography the desired amines **3 a**–**j** in yields that varied in the range 32–54 %. Comparison of the overall yields for the two‐step procedure (Table 3) with those for the one‐pot procedure indicate clearly that the latter was globally more efficient.

**Table 3:** Two‐step transformation of substrates **1 a**–**j** into **2 a**–**j** then **3 a**–**j**.

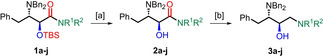				
Compound label	R^1^, R^2^	Yield (%) **2 a–j**	Yield (%) **3 a–j**	Yield (%) for 2 steps
a	H, iBu	92	34	31
b	H, nBu	91	43	39
c	H, (CH_2_)_3_Ph	82	41	34
d	H, Bn	90	47	42
e	H, allyl	87	46	40
f	H, propargyl	86	49	42
g	H, iPr	89	32	28
h	H, *c*C_3_H_5_	88	31	27
i	−C_2_H_4_OC_2_H_4_−	79	44	35
j	−(CH_2_)_4_−	80	54	43

[a] Reaction conditions: 1.5 equiv. TBAF (1 M in THF), THF, 0 °C, 1 h. [b] (i) TMSCl (1.2 equiv.), CH_2_Cl_2_, 0 °C; 15 min; (ii) LiAlH_4_ (1 M in THF, 1.4 equiv.), CH_2_Cl_2_, 0 °C, 1.5 h.

## Conclusions

This work describes an easy‐to‐apply procedure that combines *N*‐protected L‐phenylalaninal and an amine to provide *N* ^3^‐protected *N* ^1^‐substituted derivatives of *anti*‐(2*R*,3*S*)‐1,3‐diamino‐4‐phenylbutan‐2‐ol, which are important building blocks in medicinal chemistry. It requires only two steps and represents the most rapid access to the target molecule family described so far. The process is completely diastereoselective and bypasses the habitual intermediate, an L‐Phe‐derived epoxide. These notable characteristics may be advantageous in the future conception of appropriate synthetic approaches for accessing HEA drug building blocks and various related molecules.

## Experimental Section

### General Information

Preparative flash chromatography was performed using columns packed with Macherey‐Nagel (40–63 μm) silica gel. Analytical thin‐layer chromatography, used to monitor preparative flash chromatography and to provide characteristic retention factors (*R_f_*), was performed on 0.25 mm commercial silica gel plates (Merck 60F‐254); plates were visualized by UV fluorescence at 254 nm and then revealed by heating after dipping in ninhydrin solution (1.5 % in *n*‐BuOH) or KMnO_4_ solution (7.5 % in water). ^1^H and ^13^C NMR spectra were recorded on Bruker Avance I 300 or Bruker Neo 300 spectrometers (300 and 75.5 MHz, respectively), or a Bruker Avance I 400 spectrometer (400 and 100.6 MHz, respectively). Chemical shifts (δ) are given in parts per million, using solvent signals as internal standards (CDCl_3_: δ_H_=7.26 ppm, δ_C_=77.0 ppm). Assignments were aided by JMOD and 2D experiments (HSQC, COSY). Splitting patterns for ^1^H signals are designated as s (singlet), d (doublet), t (triplet), q (quartet), br s (broad singlet), or m (multiplet). Coupling constants (*J*) are reported in hertz (Hz). Positive electrospray (ES^+^) high resolution mass spectra (HRMS) were recorded using a Bruker Daltonics MicroTOF‐Q instrument or a Shimadzu LCMS‐9030 Q‐TOF instrument. Infrared (IR) spectra were recorded on a FT‐IR Perkin‐Elmer Spectrum Two spectrophotometer using an ATR diamond accessory; maximum absorbances (ν) are given in cm^−1^. Melting points (Mp) were determined with a Büchi M‐560 apparatus in open capillary tubes and are uncorrected. Optical rotations were measured on a Jasco P‐1010 polarimeter using a 10 cm quartz cell; values for [α]_D_^*T*^ were obtained with the D‐line of sodium at the indicated temperature *T*, using solutions of concentration (*c*) in units of g.100 mL^−1^.

H‐MAC‐TBS was prepared from malononitrile according to the literature procedure.^[18c]^ *N*,*N*‐Dibenzyl‐L‐phenylalaninal was prepared from commercial (*S*)‐phenylalaninol immediately before use, according to the literature procedure.^[26]^ Carboxamides **1 a**–**b, 1 d** and **1 f**–**j** were prepared via a MAC reaction as previously described;^[18a]^ carboxamides **1 c** and **1 e** are new compounds and their characterization is given below. Et_2_O was distilled under argon from Na/benzophenone. CH_2_Cl_2_ and TMSCl were distilled under argon from CaH_2_. All other solvents and reagents were obtained commercially and were used directly as supplied.

### General Procedure for MAC Reactions

To freshly prepared *N*,*N*‐dibenzyl‐L‐phenylalaninal (~0.5 mmol, 1 equiv.) and H‐MAC‐TBS (2.4 equiv.) under argon, was added Et_2_O (5 mL). After cooling at 0 °C, the amine (1.2 equiv.) was introduced followed by DMAP (2 equiv.) in one portion. The reaction mixture was stirred overnight under argon at 0 °C. A saturated aqueous Na_2_CO_3_ solution (5 mL) was added, followed by water (5 mL) if salts precipitate. The aqueous phase was extracted with Et_2_O (6×10 mL) and the combined organic phases were washed with 1 M HCl (20 mL), brine (20 mL), dried over Na_2_SO_4_ and concentrated under reduced pressure. The crude residue was purified by flash column chromatography to give the corresponding *anti* MAC reaction products.

### (2*S*,3*S*)‐*N*‐(3‐phenylpropyl)‐2‐(*tert*‐butyldimethylsilyloxy)‐3‐(dibenzylamino)‐4‐phenylbutanamide (1 c)

MAC reaction was performed with *N*,*N*‐dibenzyl‐L‐phenylalaninal (167 mg, 0.51 mmol), H‐MAC‐TBS (245 mg, 1.2 mmol), 3‐phenylpropylamine (87 μL, 0.61 mmol) and DMAP (122 mg, 1.0 mmol) in Et_2_O (5 mL). Flash chromatography (pentane/Et_2_O/CH_2_Cl_2_, 10 : 1.5 : 2) gave the *anti* MAC product **1 c** (dr>98 : 2, 212 mg, 69 %) as a pale yellow solid. Mp 90–93 °C. *R_f_* 0.31 (pentane/Et_2_O/CH_2_Cl_2_, 10 : 1.5 : 2). [α]_D_^26^=−27.5 (*c* 1.0 in CHCl_3_). IR (ATR): ν 3427, 3059, 3026, 2929, 2854, 1657, 1519, 1456, 1255, 1069 cm^−1^. HRMS (ES^+^): calcd. for C_39_H_51_N_2_O_2_Si [M+H]^+^ 607.3714; found 607.3717. ^1^H NMR (400 MHz, CDCl_3_), δ −0.10 (s, 3H, SiCH_3_), 0.02 (s, 3H, SiCH_3_), 0.93 (s, 9H, SiC(CH_3_)_3_), 1.66–1.76 (m, 2H, NCH_2_C*H*_2_CH_2_Ph), 2.56–2.65 (m, 2H, NCH_2_CH_2_C*H*_2_Ph), 2.85–2.96 (m, 1H) and 3.27–3.33 (m, 1H) (AB syst., NC*H*_2_CH_2_CH_2_Ph), 3.07 (br d, *J*=7.2 Hz, 2H, PhC*H*_2_CH), 3.33–3.38 (m, 1H, C*H*NBn_2_), 3.80 (s, 4H, N(C*H*_2_Ph)_2_), 4.39 (d, *J*=2.7 Hz, 1H, C*H*OTBS), 6.44 (br t, *J*=5.4 Hz, 1H, CONH), 7.12–7.34 (m, 20H, Ph). ^13^C NMR (100.6 MHz, CDCl_3_), δ −5.2 (SiCH_3_), −4.8 (SiCH_3_), 18.0 (Si*C*(CH_3_)_3_), 25.9 (SiC(*C*H_3_)_3_), 30.9 (NCH_2_ *C*H_2_CH_2_Ph), 32.0 (Ph*C*H_2_CH), 33.3 (NCH_2_CH_2_ *C*H_2_Ph), 38.5 (N*C*H_2_CH_2_CH_2_Ph), 54.6 (N(*C*H_2_Ph)_2_), 64.2 (*C*HNBn_2_), 72.5 (*C*HOTBS), 125.8 (CH_Ph_), 125.9 (CH_Ph_), 126.6 (CH_Ph_), 128.0 (CH_Ph_), 128.1 (CH_Ph_), 128.2 (CH_Ph_), 128.3 (CH_Ph_), 128.6 (CH_Ph_), 129.6 (CH_Ph_), 139.7 (C_Ph_), 139.9 (C_Ph_), 141.1 (C_Ph_), 172.9 (CONH).

### (2*S*,3*S*)‐*N*‐Allyl‐2‐(*tert*‐butyldimethylsilyloxy)‐3‐(dibenzylamino)‐4‐phenylbutanamide (1 e)

MAC reaction was performed with *N*,*N*‐dibenzyl‐L‐phenylalaninal (148 mg, 0.45 mmol), H‐MAC‐TBS (212 mg, 1.1 mmol), allylamine (40 μL, 0.54 mmol) and DMAP (110 mg, 0.90 mmol) in Et_2_O (5 mL). Flash chromatography (pentane/Et_2_O/CH_2_Cl_2_, 10 : 1 : 2) gave the *anti* MAC product **1 e** (dr>98 : 2, 169 mg, 71 %), as a pale yellow oil. *R_f_* 0.28 (pentane/Et_2_O/CH_2_Cl_2_, 10 : 1 : 2). [α]_D_^23^=−29.7 (*c* 1.0 in CHCl_3_). IR (ATR): ν 3425, 3081, 3058, 3026, 2945, 2927, 2859, 1663, 1514, 1451, 1256, 1106 cm^−1^. HRMS (ES^+^): calcd. for C_33_H_45_N_2_O_2_Si [M+H]^+^ 529.3244; found 529.3219. ^1^H NMR (300 MHz, CDCl_3_), δ −0.13 (s, 3H, SiCH_3_), −0.01 (s, 3H, SiCH_3_), 0.89 (s, 9H, SiC(CH_3_)_3_), 3.04 (br d, *J*=7.5 Hz, 2H, PhC*H*_2_CH), 3.32 (td, *J*=7.5, 3.0 Hz, 1H, C*H*NBn_2_), 3.40–3.50 (m, 1H) and 3.84–3.95 (m, 1H) (AB syst., NC*H*_2_CH=CH_2_), 3.77 (s, 4H, N(C*H*_2_Ph)_2_), 4.38 (d, *J*=3.0 Hz, 1H, C*H*OTBS), 5.06–5.19 (m, 2H, NCH_2_CH=C*H*_2_), 5.68 (ddt, *J*=16.8, 10.5, 6.3 Hz, 1H, NCH_2_C*H*=CH_2_), 7.11 (br t, *J*=5.7 Hz, 1H, CONH), 7.14–7.37 (m, 15H, Ph). ^13^C NMR (75.5 MHz, CDCl_3_), δ −5.1 (SiCH_3_), −4.7 (SiCH_3_), 18.1 (Si*C*(CH_3_)_3_), 25.9 (SiC(*C*H_3_)_3_), 32.1 (Ph*C*H_2_CH), 41.6 (N*C*H_2_CH=CH_2_), 54.6 (N(*C*H_2_Ph)_2_), 64.2 (*C*HNBn_2_), 72.7 (*C*HOTBS), 117.4 (NCH_2_CH=*C*H_2_), 125.9 (CH_Ph_), 126.7 (CH_Ph_), 128.1 (CH_Ph_), 128.2 (CH_Ph_), 128.7 (CH_Ph_), 129.7 (CH_Ph_), 133.6 (NCH_2_ *C*H=CH_2_), 139.7 (C_Ph_), 140.0 (C_Ph_), 172.8 (CONH).

### General Procedure for TBS Ether Cleavage

To a stirred solution of MAC reaction product **1 a**–**j** (~0.2 mmol, 1 equiv.) in THF (2 mL) under argon at 0 °C, was added dropwise tetrabutylammonium fluoride (1 M in THF, 1.5 equiv.). After 1 hour at 0 °C (completion of the reaction was monitored by TLC), the mixture was quenched by addition of a saturated aqueous NH_4_Cl solution (5 mL). The mixture was extracted with EtOAc (3×10 mL) and the combined organic phases were dried over Na_2_SO_4_ and concentrated under reduced pressure. Purification by flash column chromatography (pentane/EtOAc 3 : 1 then EtOAc, unless otherwise indicated) gave the corresponding *anti* alcohol **2 a**–**j**.

### (2*S*,3*S*)‐*N*‐Isobutyl‐3‐(dibenzylamino)‐2‐hydroxy‐4‐phenylbutanamide (2 a)

*O*‐TBS cleavage of *anti* MAC product **1 a** (103 mg, 0.19 mmol) was performed with TBAF (285 μL, 0.28 mmol) in THF (1.9 mL). Flash chromatography gave the *anti* alcohol **2 a** (75 mg, 92 %), as a viscous pale yellow oil. *R_f_* 0.10 (pentane/EtOAc, 5 : 1). [α]_D_^21^=−11.1 (*c* 1.0 in CHCl_3_). IR (ATR): ν 3391, 3061, 3029, 2960, 2919, 2873, 2859, 2795, 1645, 1526, 1494, 1453, 1270, 1123, 1072 cm^−1^. HRMS (ES^+^): calcd. for C_28_H_35_N_2_O_2_ [M+H]^+^ 431.2693; found 431.2674. ^1^H NMR (300 MHz, CDCl_3_), δ 0.83 (d, *J*=6.8 Hz, 6H, NCH_2_CH(C*H*_3_)_2_), 1.63 (nonuplet, *J*=6.7 Hz, 1H, NCH_2_C*H*(CH_3_)_2_), 2.95 (t, *J*=6.6 Hz, 2H, NC*H*_2_CH(CH_3_)_2_), 3.15 (dd, *J*=11.5, 4.1 Hz, 1H) and 3.35 (dd, *J*=11.5, 8.7 Hz, 1H) (AB syst., PhC*H*_2_CH), 3.25–3.30 (m, 1H, C*H*NBn_2_), 3.63 (d, *J*=13.5 Hz, 2H) and 3.82 (d, *J*=13.5 Hz, 2H) (AB syst., N(C*H*_2_Ph)_2_), 3.76 (br s, 1H, CHO*H*), 3.99 (br d, *J*=3.9 Hz, 1H, C*H*OH), 6.91 (br t, *J*=6.3 Hz, 1H, CONH), 7.23–7.7.37 (m, 15H, Ph). ^13^C NMR (75.5 MHz, CDCl_3_), δ 20.0 (NCH_2_CH*C*H_3_), 20.1 (NCH_2_CH*C*H_3_), 28.2 (NCH_2_ *C*H(CH_3_)_2_), 30.6 (Ph*C*H_2_CH), 46.5 (N*C*H_2_CH(CH_3_)_2_), 54.8 (N(*C*H_2_Ph)_2_), 63.3 (*C*HNBn_2_), 68.6 (CHOH), 126.0 (CH_Ph_), 127.3 (CH_Ph_), 128.3 (CH_Ph_), 128.5 (CH_Ph_), 128.8 (CH_Ph_), 129.6 (CH_Ph_), 139.1 (C_Ph_), 139.8 (C_Ph_), 173.1 (CONH).

### (2*S*,3*S*)‐*N*‐Butyl‐3‐(dibenzylamino)‐2‐hydroxy‐4‐phenylbutanamide (2 b)

*O*‐TBS cleavage of *anti* MAC product **1 b** (108 mg, 0.20 mmol) was performed with TBAF (300 μL, 0.30 mmol) in THF (2 mL). Flash chromatography gave the *anti* alcohol **2 b** (78 mg, 91 %), as a viscous pale yellow oil. *R_f_* 0.12 (pentane/EtOAc, 5 : 1). [α]_D_^26^=−7.3 (*c* 1.0 in CHCl_3_). IR (ATR): ν 3399, 3067, 3031, 2962, 2926, 2876, 2803, 1642, 1533, 1495, 1451, 1369, 1274, 1128, 1074 cm^−1^. HRMS (ES^+^): calcd. for C_28_H_35_N_2_O_2_ [M+H]^+^ 431.2693; found 431.2672. ^1^H NMR (300 MHz, CDCl_3_), δ 0.83 (t, *J*=7.1 Hz, 3H, NCH_2_CH_2_CH_2_C*H*_3_), 1.17–1.36 (m, 4H, NCH_2_C*H*_2_C*H*_2_CH_3_), 3.01–3.12 (m, 2H, NC*H*_2_CH_2_CH_2_CH_3_), 3.17 (dd, *J*=12.8, 5.7 Hz, 1H) and 3.39 (dd, *J*=12.8, 8.6 Hz, 1H) (AB syst., PhC*H*_2_CH), 3.22–3.31 (m, 1H, C*H*NBn_2_), 3.60 (d, *J*=13.6 Hz, 2H) and 3.84 (d, *J*=13.6 Hz, 2H) (AB syst., N(C*H*_2_Ph)_2_), 3.75 (br s, 1H, CHO*H*), 3.92 (br d, *J*=4.5 Hz, 1H, C*H*OH), 6.91 (br t, *J*=5.5 Hz, 1H, CONH), 7.15–7.46 (m, 15H, Ph). ^13^C NMR (75.5 MHz, CDCl_3_), δ 13.6 (NCH_2_CH_2_CH_2_ *C*H_3_), 20.1 (NCH_2_CH_2_ *C*H_2_CH_3_), 30.5 (Ph*C*H_2_CH), 31.3 (NCH_2_ *C*H_2_CH_2_CH_3_), 38.8 (N*C*H_2_CH_2_CH_2_CH_3_), 54.9 (N(*C*H_2_Ph)_2_), 63.5 (*C*HNBn_2_), 68.5 (CHOH), 126.0 (CH_Ph_), 126.1 (CH_Ph_), 127.4 (CH_Ph_), 128.3 (CH_Ph_), 128.5 (CH_Ph_), 128.8 (CH_Ph_), 129.7 (CH_Ph_), 139.0 (C_Ph_), 139.8 (C_Ph_), 173.0 (CONH).

### (2*S*,3*S*)‐*N*‐(3‐phenylpropyl)‐3‐(dibenzylamino)‐2‐hydroxy‐4‐phenylbutanamide (2 c)

*O*‐TBS cleavage of *anti* MAC product **1 c** (121 mg, 0.20 mmol) was performed with TBAF (300 μL, 0.30 mmol) in THF (2 mL). Flash chromatography (pentane/EtOAc, 3 : 1; then 2 : 1) gave *anti* alcohol **2 c** (81 mg, 82 %) as a sticky whitish oil. *R_f_* 0.29 (pentane/EtOAc, 3 : 1). [α]_D_^26^=−14.2 (*c* 1.0 in CHCl_3_). IR (ATR): ν 3382, 3286, 3056, 3026, 2926, 2863, 2798, 1645, 1527, 1496, 1451, 1365, 1252, 1119, 1075 cm^−1^. HRMS (ES^+^): calcd. for C_33_H_37_N_2_O_2_ [M+H]^+^ 493.2849; found 493.2863. ^1^H NMR (400 MHz, CDCl_3_), δ 1.61–1.70 (m, 2H, NCH_2_C*H*_2_CH_2_Ph), 2.52 (t, *J*=7.7 Hz, 2H, NCH_2_CH_2_C*H*_2_Ph), 3.04–3.22 (m, 3H) and 3.38 (dd, *J*=13.0, 8.9 Hz, 1H) (AB syst. PhC*H*_2_CH, NC*H*_2_CH_2_CH_2_Ph), 3.23–3.29 (m, 1H, C*H*NBn_2_), 3.59 (d, *J*=13.7 Hz, 2H) and 3.82 (d, *J*=13.7 Hz, 2H) (AB syst., N(C*H*_2_Ph)_2_), 3.83 (br s, 1H, CHO*H*), 3.90 (br d, *J*=4.8 Hz, 1H, C*H*OH), 6.96 (br t, *J*=5.6 Hz, 1H, CONH), 7.02–7.07 (m, 2H, Ph), 7.17–7.38 (m, 18H, Ph). ^13^C NMR (100.6 MHz, CDCl_3_), δ 30.4 (Ph*C*H_2_CH), 30.8 (NCH_2_ *C*H_2_CH_2_Ph), 33.1 (NCH_2_CH_2_ *C*H_2_Ph), 38.6 (N*C*H_2_CH_2_CH_2_Ph), 54.8 (N(*C*H_2_Ph)_2_), 63.4 (*C*HNBn_2_), 68.5 (CHOH), 125.9 (CH_Ph_), 126.1 (CH_Ph_), 127.4 (CH_Ph_), 128.2 (CH_Ph_), 128.3 (CH_Ph_), 128.6 (CH_Ph_), 128.8 (CH_Ph_), 129.6 (CH_Ph_), 139.0 (C_Ph_), 139.7 (C_Ph_), 141.1 (C_Ph_), 173.1 (CONH).

### (2*S*,3*S*)‐*N*‐Benzyl‐3‐(dibenzylamino)‐2‐hydroxy‐4‐phenylbutanamide (2 d)

*O*‐TBS cleavage of *anti* MAC product **1 d** (100 mg, 0.17 mmol) was performed with TBAF (260 μL, 0.26 mmol) in THF (1.7 mL). Flash chromatography gave the *anti* alcohol **2 d** (72 mg, 90 %), as a viscous pale yellow oil. *R_f_* 0.10 (pentane/EtOAc, 5 : 1). [α]_D_^26^=−5.0 (*c* 1.0 in CHCl_3_). IR (ATR): ν 3379, 3062, 3031, 2927, 2800, 1650, 1523, 1495, 1451, 1256, 1099, 1030 cm^−1^. HRMS (ES^+^): calcd. for C_31_H_33_N_2_O_2_ [M+H]^+^ 465.2536; found 465.2514. ^1^H NMR (300 MHz, CDCl_3_), δ 3.17 (dd, *J*=12.8, 5.1 Hz, 1H) and 3.43 (dd, *J*=12.8, 8.8 Hz, 1H) (AB syst., PhC*H*_2_CH), 3.27–3.36 (m, 1H, C*H*NBn_2_), 3.60 (d, *J*=13.6 Hz, 2H) and 3.85 (d, *J*=13.6 Hz, 2H) (AB syst., N(C*H*_2_Ph)_2_), 3.75 (br s, 1H, CHO*H*), 3.99 (d, *J*=4.9 Hz, 1H, C*H*OH), 4.24 (dd, *J*=14.5, 5.5 Hz, 1H) and 4.35 (dd, *J*=14.5, 6.0 Hz, 1H) (AB syst., NHC*H*_2_Ph), 7.09–7.44 (m, 21H, Ph, CONH). ^13^C NMR (75.5 MHz, CDCl_3_), δ 30.5 (Ph*C*H_2_CH), 43.3 (NH*C*H_2_Ph), 54.8 (N(*C*H_2_Ph)_2_), 63.5 (*C*HNBn_2_), 68.6 (CHOH), 126.1 (CH_Ph_), 127.3 (CH_Ph_), 127.4 (CH_Ph_), 127.9 (CH_Ph_), 128.3 (CH_Ph_), 128.6 (CH_Ph_), 128.8 (CH_Ph_), 129.6 (CH_Ph_), 137.5 (C_Ph_), 138.9 (C_Ph_), 139.8 (C_Ph_), 173.0 (CONH).

### (2*S*,3*S*)‐*N*‐Allyl‐3‐(dibenzylamino)‐2‐hydroxy‐4‐phenylbutanamide (2 e)

*O*‐TBS cleavage of *anti* MAC product **1 e** (99 mg, 0.19 mmol) was performed with TBAF (280 μL, 0.28 mmol) in THF (1.9 mL). Flash chromatography gave the *anti* alcohol **2 e** (67 mg, 87 %), as a viscous pale yellow oil. *R_f_* 0.21 (pentane/EtOAc, 5 : 1). [α]_D_^25^=−1.7 (*c* 1.0 in CHCl_3_). IR (ATR): ν 3384, 3307, 3058, 3026, 2913, 2800, 1641, 1518, 1496, 1451, 1364, 1269, 1124 cm^−1^. HRMS (ES^+^): calcd. for C_27_H_31_N_2_O_2_ [M+H]^+^ 415.2380; found 415.2360. ^1^H NMR (300 MHz, CDCl_3_), δ 3.17 (dd, *J*=12.7, 4.9 Hz, 1H) and 3.40 (dd, *J*=12.7, 8.9 Hz, 1H) (AB syst., PhC*H*_2_CH), 3.31 (td, *J*=8.9, 4.9 Hz, 1H, C*H*NBn_2_), 3.61 (d, *J*=13.7 Hz, 2H) and 3.85 (d, *J*=13.7 Hz, 2H) (AB syst., N(C*H*_2_Ph)_2_), 3.72–3.80 (m, 2H, NC*H*_2_CH=CH_2_), 3.72–3.90 (br s, 1H, CHO*H*), 3.97 (br d, *J*=4.3 Hz, 1H, C*H*OH), 5.00–5.17 (m, 2H, NCH_2_CH=C*H*_2_), 5.66 (ddt, *J*=16.2, 10.2, 6.0 Hz, 1H, NCH_2_C*H*=CH_2_), 7.11 (br t, *J*=5.8 Hz, 1H, CONH), 7.22–7.41 (m, 15H, Ph). ^13^C NMR (75.5 MHz, CDCl_3_), δ 30.6 (Ph*C*H_2_CH), 41.5 (N*C*H_2_CH=CH_2_), 54.9 (N(*C*H_2_Ph)_2_), 63.5 (*C*HNBn_2_), 68.6 (*C*HOH), 116.9 (NCH_2_CH=*C*H_2_), 126.1 (CH_Ph_), 127.4 (CH_Ph_), 128.3 (CH_Ph_), 128.6 (CH_Ph_), 128.9 (CH_Ph_), 129.6 (CH_Ph_), 133.6 (NCH_2_ *C*H=CH_2_) 139.0 (C_Ph_), 139.7 (C_Ph_), 173.0 (CONH).

### (2*S*,3*S*)‐*N*‐(Prop‐2‐yn‐1‐yl)‐3‐(dibenzylamino)‐2‐hydroxy‐4‐phenylbutanamide (2 f)

*O*‐TBS cleavage of *anti* MAC product **1 f** (123 mg, 0.23 mmol) was performed with TBAF (350 μL, 0.35 mmol) in THF (2.3 mL). Flash chromatography gave the *anti* alcohol **2 f** (83 mg, 86 %), as a viscous yellow oil. *R_f_* 0.14 (pentane/EtOAc, 5 : 1). [α]_D_^26^=−14.2 (*c* 1.0 in CHCl_3_). IR (ATR): ν 3398, 3294, 3085, 3063, 3026, 2922, 2836, 2804, 1654, 1518, 1491, 1455, 1256, 1129, 1070 cm^−1^. HRMS (ES^+^): calcd. for C_27_H_29_N_2_O_2_ [M+H]^+^ 413.2223; found 413.2210. ^1^H NMR (300 MHz, CDCl_3_), δ 2.10 (t, *J*=2.4 Hz, 1H, NCH_2_C≡C*H*), 3.18 (dd, *J*=12.4, 4.7 Hz, 1H) and 3.41 (dd, *J*=12.4, 8.7 Hz, 1H) (AB syst., PhC*H*_2_CH), 3.34 (ddd, *J*=8.7, 5.1, 4.7 Hz, 1H, C*H*NBn_2_), 3.59 (d, *J*=13.6 Hz, 2H) and 3.86 (d, *J*=13.6 Hz, 2H) (AB syst., N(C*H*_2_Ph)_2_), 3.80–3.91 (m, 1H, CHO*H*), 3.80 (br ddd, *J*=17.5, 4.7, 2.4 Hz, 1H) and 4.03 (ddd, *J*=17.5, 6.2, 2.4 Hz, 1H) (AB syst., NC*H*_2_C≡CH), 3.98 (br d, *J*=5.1 Hz, 1H, C*H*OH), 7.22–7.47 (m, 16H, Ph, CONH).^13^C NMR (75.5 MHz, CDCl_3_), δ 28.6 (N*C*H_2_C≡CH), 30.6 (Ph*C*H_2_CH), 54.8 (N(*C*H_2_Ph)_2_), 63.3 (*C*HNBn_2_), 68.6 (CHOH), 71.5 (NCH_2_C≡*C*H), 79.0 (NCH_2_ *C*≡CH), 126.1 (CH_Ph_), 127.4 (CH_Ph_), 128.3 (CH_Ph_), 128.5 (CH_Ph_), 129.0 (CH_Ph_), 129.6 (CH_Ph_), 138.8 (C_Ph_), 139.7 (C_Ph_), 172.9 (CONH).

### (2*S*,3*S*)‐*N*‐Isopropyl‐3‐(dibenzylamino)‐2‐hydroxy‐4‐phenylbutanamide (2 g)

*O*‐TBS cleavage of *anti* MAC product **1 g** (76 mg, 0.14 mmol) was performed with TBAF (215 μL, 0.21 mmol) in THF (1.4 mL). Flash chromatography gave the *anti* alcohol **2 g** (53 mg, 89 %), as a viscous pale yellow oil. *R_f_* 0.10 (pentane/EtOAc, 5 : 1). [α]_D_^23^=−23.7 (*c* 1.0 in CHCl_3_). IR (ATR): ν 3379, 3062, 3031, 2972, 2936, 2795, 1636, 1523, 1505, 1455, 1369, 1124, 1097 cm^−1^. HRMS (ES^+^): calcd. for C_27_H_33_N_2_O_2_ [M+H]^+^ 417.2536; found 417.2525. ^1^H NMR (300 MHz, CDCl_3_), δ 0.93 (d, *J*=6.4 Hz, 3H, NCHC*H*_3_), 1.08 (d, *J*=6.4 Hz, 3H, NCHC*H*_3_), 3.15 (dd, *J*=12.6, 5.1 Hz, 1H) and 3.39 (dd, *J*=12.6, 8.8 Hz, 1H) (AB syst., PhC*H*_2_CH), 3.27 (ddd, *J*=8.8, 5.1, 4.3 Hz, 1H, C*H*NBn_2_), 3.63 (d, *J*=13.7 Hz, 2H) and 3.84 (d, *J*=13.7 Hz, 2H) (AB syst., N(C*H*_2_Ph)_2_), 3.76 (br s, 1H, CHO*H*), 3.90 (d, *J*=4.3 Hz, 1H, C*H*OH), 3.93–4.05 (m, 1H, NC*H*(CH_3_)_2_), 6.67 (br d, *J*=7.8 Hz, 1H, CONH), 7.20–7.43 (m, 15H, Ph). ^13^C NMR (75.5 MHz, CDCl_3_), δ 22.2 (NCH*C*H_3_), 22.5 (NCH*C*H_3_), 30.3 (Ph*C*H_2_CH), 41.0 (N*C*H(CH_3_)_2_), 54.9 (N(*C*H_2_Ph)_2_), 63.5 (*C*HNBn_2_), 68.4 (CHOH), 126.0 (CH_Ph_), 127.4 (CH_Ph_), 128.3 (CH_Ph_), 128.5 (CH_Ph_), 128.8 (CHPh), 129.6 (CH_Ph_), 139.0 (C_Ph_), 139.7 (C_Ph_), 172.1 (CONH).

### (2*S*,3*S*)‐*N*‐Cyclopropyl‐3‐(dibenzylamino)‐2‐hydroxy‐4‐phenylbutanamide (2 h)

*O*‐TBS cleavage of *anti* MAC product **1 h** (96 mg, 0.18 mmol) was performed with TBAF (270 μL, 0.27 mmol) in THF (1.8 mL). Flash chromatography gave the *anti* alcohol **2 h** (66 mg, 88 %) as a white solid. Mp 147–149 °C. *R_f_* 0.17 (pentane/EtOAc, 3 : 1). [α]_D_^25^=−11.4 (*c* 1.0 in CHCl_3_). IR (ATR): ν 3392, 3271, 3025, 2922, 2852, 2794, 1649, 1514, 1451, 1363, 1243, 1112 cm^−1^. HRMS (ES^+^): calcd. for C_27_H_31_N_2_O_2_ [M+H]^+^ 415.2380; found 415.2387. ^1^H NMR (400 MHz, CDCl_3_), δ 0.14–0.24 (m, 1H) and 0.28–0.37 (m, 1H) (AB syst., NCHC*H*_2_), 0.60–0.66 (m, 1H) and 0.66–0.72 (m, 1H) (AB syst., NCHC*H*_2_), 2.51–2.61 (m, 1H, NC*H*(CH_2_)_2_), 3.15 (dd, *J*=13.2, 5.0 Hz, 1H) and 3.44 (dd, *J*=13.2, 9.1 Hz, 1H) (AB syst., PhC*H*_2_CH), 3.19–3.26 (m, 1H, C*H*NBn_2_), 3.57 (d, *J*=13.6 Hz, 2H) and 3.83 (d, *J*=13.6 Hz, 2H) (AB syst., N(C*H*_2_Ph)_2_), 3.66 (br d, *J*=7.3 Hz, 1H, CHO*H*), 3.84 (br s, 1H, C*H*OH), 7.08 (br s, 1H, CONH), 7.20–7.44 (m, 15H, Ph). ^13^C NMR (100.6 MHz, CDCl_3_), δ 5.6 (NCH*C*H_2_), 6.2 (NCH*C*H_2_), 21.9 (N*C*H(CH_2_)_2_), 30.3 (Ph*C*H_2_CH), 54.9 (N(*C*H_2_Ph)_2_), 63.5 (*C*HNBn_2_), 68.4 (CHOH), 126.1 (CH_Ph_), 127.4 (CH_Ph_), 128.3 (CH_Ph_), 128.6 (CH_Ph_), 128.8 (CH_Ph_), 129.6 (CH_Ph_), 138.9 (C_Ph_), 139.6 (C_Ph_), 174.6 (CONH).

### (2*S*,3*S*)‐3‐(Dibenzylamino)‐2‐hydroxy‐1‐morpholino‐4‐phenylbutan‐1‐one (2 i)

*O*‐TBS cleavage of *anti* MAC product **1 i** (91 mg, 0.16 mmol) was performed with TBAF (250 μL, 0.25 mmol) in THF (1.6 mL). Flash chromatography gave the *anti* alcohol **2 i** (57 mg, 79 %), as a viscous pale yellow oil. *R_f_* 0.20 (pentane/EtOAc, 5 : 1). [α]_D_^25^=+29.0 (*c* 1.0 in CHCl_3_). IR (ATR): ν 3411, 3058, 3026, 2967, 2922, 2854, 2804, 1641, 1497, 1455, 1392, 1274, 1120, 1029 cm^−1^. HRMS (ES^+^): calcd. for C_28_H_33_N_2_O_3_ [M+H]^+^ 445.2485; found 445.2469. ^1^H NMR (300 MHz, CDCl_3_), δ 2.49–2.58 (m, 1H) and 2.64–2.82 (m, 1H) (AB syst., NC*H*_2_CH_2_O), 2.64–2.82 (m, 1H) and 3.01–3.17 (m, 1H) (AB syst., NCH_2_C*H*_2_O), 2.64–2.82 (m, 1H) and 3.01–3.17 (m, 1H) (AB syst., PhC*H*_2_CH), 3.01–3.17 (m, 1H, C*H*NBn_2_), 3.01–3.17 (m, 1H) and 3.28–3.37 (m, 1H) (AB syst., NC*H*_2_CH_2_O), 3.01–3.17 (m, 1H) and 3.46–3.55 (m, 1H) (AB syst., NCH_2_C*H*_2_O), 3.83 (d, *J*=14.4 Hz, 2H) and 3.96 (d, *J*=14.4 Hz, 2H) (AB syst., N(C*H*_2_Ph)_2_), 4.21 (d, *J*=6.0 Hz, 1H, CHO*H*), 4.71 (d, *J*=6.0 Hz, 1H, C*H*OH), 7.10–7.16 (m, 2H, Ph), 7.19–7.36 (m, 13H, Ph). ^13^C NMR (75.5 MHz, CDCl_3_), δ 30.4 (Ph*C*H_2_CH), 42.6 (N*C*H_2_CH_2_O), 44.7 (N*C*H_2_CH_2_O), 54.5 (N(*C*H_2_Ph)_2_), 60.9 (*C*HNBn_2_), 65.5 (NCH_2_ *C*H_2_O), 66.2 (NCH_2_ *C*H_2_O), 70.1 (CHOH), 126.1 (CH_Ph_), 126.9 (CH_Ph_), 128.0 (CH_Ph_), 128.2 (CH_Ph_), 128.5 (CH_Ph_), 129.7 (CH_Ph_), 140.0 (C_Ph_), 140.1 (C_Ph_), 171.7 (CONH).

### (2*S*,3*S*)‐3‐(Dibenzylamino)‐2‐hydroxy‐4‐phenyl‐1‐(pyrrolidin‐1‐yl)butan‐1‐one (2 j)

*O*‐TBS cleavage of *anti* MAC product **1 h** (84 mg, 0.15 mmol) was performed with TBAF (230 μL, 0.23 mmol) in THF (1.5 mL). Flash chromatography gave the *anti* alcohol **2 j** (53 mg, 80 %), as a viscous pale yellow oil. *R_f_* 0.11 (pentane/EtOAc, 5 : 1). [α]_D_^25^=+37.3 (*c* 1.0 in CHCl_3_). IR (ATR): ν 3393, 3090, 3063, 3026, 2963, 2881, 2798, 1632, 1491, 1451, 1378, 1102, 1029 cm^−1^. HRMS (ES^+^): calcd. for C_28_H_33_N_2_O_2_ [M+H]^+^ 429.2536; found 429.2516. ^1^H NMR (300 MHz, CDCl_3_), δ 1.45–1.76 (m, 4H, N(CH_2_C*H*_2_)_2_), 2.15–2.27 (m, 1H) and 2.92–3.02 (m, 1H) (AB syst., NC*H*_2_CH_2_), 2.78 (dd, *J*=14.2, 5.9 Hz, 1H) and 3.06 (dd, *J*=14.2, 7.3 Hz, 1H) (AB syst., PhC*H*_2_CH), 2.92–3.02 (m, 1H) and 3.28–3.40 (m, 1H) (AB syst., NC*H*_2_CH_2_), 3.18–3.26 (m, 1H, C*H*NBn_2_), 3.88 (d, *J*=14.4 Hz, 2H) and 3.95 (d, *J*=14.4 Hz, 2H) (AB syst., N(C*H*_2_Ph)_2_), 4.24 (br s, 1H, CHO*H*), 4.66 (br s, 1H, C*H*OH), 7.08–7.14 (m, 2H, Ph), 7.17–7.32 (m, 13H, Ph). ^13^C NMR (75.5 MHz, CDCl_3_), δ 23.4 (NCH_2_ *C*H_2_), 25.8 (NCH_2_ *C*H_2_), 30.9 (Ph*C*H_2_CH), 45.4 (N*C*H_2_CH_2_), 46.2 (N*C*H_2_CH_2_), 54.6 (N(*C*H_2_Ph)_2_), 59.7 (*C*HNBn_2_), 71.0 (CHOH), 125.9 (CH_Ph_), 126.7 (CH_Ph_), 127.9 (CH_Ph_), 128.1 (CH_Ph_), 128.5 (CH_Ph_), 129.6 (CH_Ph_), 140.1 (C_Ph_), 140.3 (C_Ph_), 171.3 (CONH).

### General Procedure for the Reduction Step

To MAC reaction product **1 a–j** or TBS‐deprotected MAC reaction product **2 a–j** (~0.2 mmol, 1 equiv.) under argon, was added dry CH_2_Cl_2_ (1.5 mL). After cooling at 0 °C, TMSCl (1.2 equiv.) was added dropwise and the reaction mixture was stirred at this temperature for 15 minutes. LiAlH_4_ (1 M in THF; 1.4 equiv.) was slowly introduced and the reaction mixture was stirred at 0 °C for 1.5 hours. After dropwise addition of a 2 M NaOH solution (5 mL) at 0 °C and stirring for 30 minutes at this temperature, CH_2_Cl_2_ (5 mL) was added. The aqueous phase was extracted with CH_2_Cl_2_ (6×10 mL) and the combined organic phases were dried over Na_2_SO_4_ and concentrated under reduced pressure. The crude residue was purified by flash column chromatography (pentane/EtOAc 1 : 2 then EtOAc, unless otherwise indicated) to afford the corresponding *anti* 1,3‐diamino‐4‐phenylbutan‐2‐ol derivative **3 a–j**.

### (2*R*,3*S*)‐3‐(Dibenzylamino)‐1‐(isobutylamino)‐4‐phenylbutan‐2‐ol (3 a)

From **1 a**: reduction of *anti* MAC product **1 a** (101 mg, 0.19 mmol) was performed with TMSCl (28 μL, 0.22 mmol) and LiAlH_4_ (260 μL, 0.26 mmol) in CH_2_Cl_2_ (1.5 mL). Flash chromatography gave *anti* DAPB **3 a** (62 mg, 80 %). From **2 a**: reduction of *anti* alcohol **2 a** (69 mg, 0.16 mmol) was performed with TMSCl (24 μL, 0.19 mmol) and LiAlH_4_ (224 μL, 0.22 mmol) in CH_2_Cl_2_ (1.3 mL). Flash chromatography gave *anti* DAPB **1 a** (23 mg, 34 %). Pale yellow oil. *R_f_* 0.10 (pentane/EtOAc, 1 : 2). [α]_D_^23^=+4.1 (*c* 1.05 in CHCl_3_), lit.^[14]^ +4.7 (*c* 1.05 in CHCl_3_). IR (ATR): ν 3367, 3084, 3062, 3027, 2958, 2929, 2855, 2804, 1604, 1497, 1455, 1362, 1248, 1199, 1121, 1027 cm^−1^. HRMS (ES^+^): calcd. for C_28_H_37_N_2_O [M+H]^+^ 417.2900; found 417.2885. ^1^H NMR (400 MHz, CDCl_3_), 0.91 (d, *J*=6.6 Hz, 3H, NCH_2_CHC*H*_3_), 0.92 (d, *J*=6.6 Hz, 3H, NCH_2_CHC*H*_3_), 1.68 (nonuplet, *J*=6.6 Hz, 1H, NCH_2_C*H*(CH_3_)_2_), 2.35 (dd, *J*=11.6, 6.6 Hz, 1H) and 2.42 (dd, *J*=11.6, 6.6 Hz, 1H) (AB syst., NC*H*_2_CH(CH_3_)_2_), 2.50 (dd, *J*=12.1, 9.1 Hz, 1H) and 2.77 (dd, *J*=12.1, 3.6 Hz, 1H) (AB syst., CH_2_N), 2.57 (br s, 2H, CHO*H*, NH), 2.85–2.91 (m, 1H, C*H*NBn_2_), 3.01 (dd, *J*=14.3, 5.0 Hz, 1H) and 3.09 (dd, *J*=14.3, 8.2 Hz, 1H) (AB syst., PhC*H*_2_CH), 3.66 (d, *J*=13.8 Hz, 2H) and 3.73 (d, *J*=13.8 Hz, 2H) (AB syst., N(C*H*_2_Ph)_2_), 3.87–3.96 (m, 1H, C*H*OH), 7.13–7.37 (m, 15H, Ph). ^13^C NMR (100.6 MHz, CDCl_3_), δ 20.50 (NCH_2_CH*C*H_3_), 20.53 (NCH_2_CH*C*H_3_), 28.3 (NCH_2_ *C*H(CH_3_)_2_), 32.6 (Ph*C*H_2_CH), 52.9 (CH_2_N), 54.5 (N(*C*H_2_Ph)_2_), 57.3 (N*C*H_2_CH(CH_3_)_2_), 62.2 (*C*HNBn_2_), 68.6 (CHOH), 125.7 (CH_Ph_), 126.8 (CH_Ph_), 128.1 (CH_Ph_), 128.2 (CH_Ph_), 128.8 (CH_Ph_), 129.6 (CH_Ph_), 139.9 (C_Ph_), 141.5 (C_Ph_). NMR data were in agreement with those described in literature.^[14]^

### (2*R*,3*S*)‐1‐(Butylamino)‐3‐(dibenzylamino)‐4‐phenylbutan‐2‐ol (3 b)

From **1 b**: reduction of *anti* MAC product **1 b** (110 mg, 0.20 mmol) was performed with TMSCl (33 μL, 0.26 mmol) and LiAlH_4_ (300 μL, 0.30 mmol) in CH_2_Cl_2_ (1.5 mL). Flash chromatography gave *anti* DAPB **3 b** (48 mg, 57 %). From **2 b**: reduction of *anti* alcohol **2 b** (69 mg, 0.16 mmol) was perfor.med with TMSCl (25 μL, 0.20 mmol) and LiAlH_4_ (225 μL, 0.22. mmol) in CH_2_Cl_2_ (1.2 mL). Flash chromatography gave *anti* DAPB **3 b** (29 mg, 43 %). Whitish oil. *R_f_* 0.10 (pentane/EtOAc, 1 : 2). [α]_D_^26^=−0.5 (*c* 1.0 in CHCl_3_). IR (ATR): ν 3335, 3085, 3064, 3027, 2960, 2933, 2856, 1598, 1494, 1452, 1372, 1259, 1123, 1072, 1027 cm^−1^. HRMS (ES^+^): calcd. for C_28_H_37_N_2_O [M+H]^+^ 417.2900; found 417.2889. ^1^H NMR (400 MHz, CDCl_3_), δ 0.94 (t, *J*=7.3 Hz, 3H, NCH_2_CH_2_CH_2_C*H*_3_), 1.29–1.46 (m, 4H, NCH_2_C*H*_2_C*H*_2_CH_3_), 2.50–2.64 (m, 3H) and 2.77 (dd, *J*=11.9, 3.6 Hz, 1H) (AB syst. CH_2_N, NC*H*_2_CH_2_CH_2_CH_3_), 2.65–2.85 (m, 2H, CHO*H*, NH), 2.87–2.92 (m, 1H, C*H*NBn_2_), 3.02 (dd, *J*=14.1, 5.0 Hz, 1H) and 3.10 (dd, *J*=14.1, 8.0 Hz, 1H) (AB syst., PhC*H*_2_CH), 3.66 (d, *J*=13.8 Hz, 2H) and 3.73 (d, *J*=13.8 Hz, 2H) (AB syst., N(C*H*_2_Ph)_2_), 3.87–3.96 (m, 1H, C*H*OH), 7.15–7.37 (m, 15H, Ph). ^13^C NMR (100.6 MHz, CDCl_3_), δ 13.9 (NCH_2_CH_2_CH_2_ *C*H_3_), 20.3 (NCH_2_CH_2_ *C*H_2_CH_3_), 31.8 (NCH_2_ *C*H_2_CH_2_CH_3_), 32.6 (Ph*C*H_2_CH), 49.1 (N*C*H_2_CH_2_CH_2_CH_3_), 52.8 (CH_2_N), 54.5 (N(*C*H_2_Ph)_2_), 62.2 (*C*HNBn_2_), 68.9 (CHOH), 125.7 (CH_Ph_), 126.8 (CH_Ph_), 128.1 (CH_Ph_), 128.2 (CH_Ph_), 128.8 (CH_Ph_), 129.6 (CH_Ph_), 139.9 (C_Ph_), 141.5 (C_Ph_).

### (2*R*,3*S*)‐3‐(Dibenzy.lamino)‐4‐phenyl‐1‐((3‐phenylpropyl)amino)butan‐2‐ol (3 c)

From **1 c**: reduction of *anti* MAC product **1 c** (121 mg, 0.20 mmol) was performed with TMSCl (31 μL, 0.24 mmol) and LiAlH_4_ (280 μL, 0.28 mmol) in CH_2_Cl_2_ (1.5 mL). Flash chromatography (pentane/EtOAc 2 : 1, then EtOAc, then EtOAc+2 % MeOH) gave *anti* DAPB **3 c** (52 mg, 54 %). From **2 c**: reduction of *anti* alcohol **2 c** (94 mg, 0.19 mmol) was performed with TMSCl (31 μL, 0.24 mmol) and LiAlH_4_ (280 μL, 0.28 mmol) in CH_2_Cl_2_ (1.5 mL). Flash chromatography (pentane/EtOAc 2 : 1, then EtOAc, then EtOAc+2 % MeOH) gave *anti*‐DAPB **3 c** (37 mg, 41 %). Colorless oil. *R_f_* 0.12 (pentane/EtOAc, 1 : 2). [α]_D_^25^=−2.4 (*c* 1.0 in CHCl_3_). IR (ATR): ν 3341, 3085, 3062, 3026, 2925, 2852, 2805, 1602, 1494, 1452, 1367, 1115, 1071, 1029 cm^−1^. HRMS (ES^+^): calcd. for C_33_H_39_N_2_O [M+H]^+^ 479.3057; found 479.3066. ^1^H NMR (400 MHz, CDCl_3_), δ 1.71–1.80 (m, 2H, NCH_2_C*H*_2_CH_2_Ph), 2.44 (br s, 2H, CHO*H*, NH), 2.54–2.67 (m, 5H) and 2.74 (dd, *J*=12.1, 3.5 Hz, 1H) (AB syst. CH_2_N, NC*H*_2_CH_2_C*H*_2_Ph), 2.86–2.92 (m, 1H, C*H*NBn_2_), 3.02 (dd, *J*=14.2, 5.1 Hz, 1H) and 3.09 (dd, *J*=14.2, 8.1 H.z, 1H) (AB syst., PhC*H*_2_CH), 3.64 (d, *J*=13.7 Hz, 2H) and. 3.72 (d, *J*=13.7 Hz, 2H) (AB syst., N(C*H*_2_Ph)_2_), 3.85–3.91 (m, 1H, C*H*OH), 7.17–7.35 (m, 20H, Ph). ^13^C NMR (100.6 MHz, CDCl_3_), δ 31.5 (NCH_2_ *C*H_2_CH_2_Ph), 32.6 (Ph*C*H_2_CH), 33.4 (NCH_2_CH_2_ *C*H_2_Ph), 48.9 (N*C*H_2_CH_2_CH_2_Ph), 52.7 (CH_2_N), 54.5 (N(*C*H_2_Ph)_2_), 62.1 (*C*HNBn_2_), 69.1 (CHOH), 125.7 (CH_Ph_), 125.8 (CH_Ph_), 126.8 (CH_Ph_), 128.1 (CH_Ph_), 128.2 (CH_Ph_), 128.3 (CH_Ph_), 128.4 (CH_Ph_), 128.8 (CH_Ph_), 129.6 (CH_Ph_), 139.9 (C_Ph_), 141.5 (C_Ph_), 141.9 (C_Ph_).

### (2*R*,3*S*)‐1‐(Benzylamino)‐3‐(dibenzylamino)‐4‐phenylbutan‐2‐ol (3 d)

From **1 d**: reduction of *anti* MAC product **1 d** (96 mg, 0.17 mmol) was performed with TMSCl (25 μL, 0.20 mmol) and LiAlH_4_ (230 μL, 0.23 mmol) in CH_2_Cl_2_ (1.3 mL). Flash chromatography gave *anti* DAPB **3 d** (37 mg, 49 %). From **2 d**: reduction of *anti* alcohol **2 d** (85 mg, 0.18 mmol) was performed with TMSCl (28 μL, 0.22 mmol) and LiAlH_4_ (260 μL, 0.26 mmol) in CH_2_Cl_2_ (1.4 mL). Flash chromatography gave *anti* DAPB **3 d** (39 mg, 47 %). Pale yellow oil. *R_f_* 0.12 (pentane/EtOAc, 1 : 2). [α]_D_^26^=−8.2 (*c* 1.0 in CHCl_3_), lit.^[27]^ −10.7 (*c* 1.0 in CHCl_3_). IR (ATR):. ν 3358, 3084, 3061, 3027, 2923, 2849, 2803, 1601, 1494, 1.453, 1367, 1256, 1104, 1072, 1028 cm^−1^. HRMS (ES^+^): calcd. for C_31_H_35_N_2_O [M+H]^+^ 451.2744; found 451.2730. ^1^H NMR (300 MHz, CDCl_3_), δ 2.55 (dd, *J*=12.1, 8.9 Hz, 1H) and 2.83 (dd, *J*=12.1, 3.3 Hz, 1H) (AB syst., CH_2_N), 2.62 (br s, 2H, CHO*H*, NH), 2.86–2.93 (m, 1H, C*H*NBn_2_), 2.99 (dd, *J*=14.1, 5.1 Hz, 1H) and 3.08 (dd, *J*=14.1, 8.0 Hz, 1H) (AB syst., PhC*H*_2_CH), 3.60 (d, *J*=13.7 Hz, 2H) and 3.70 (d, *J*=13.7 Hz, 2H) (AB syst., N(C*H*_2_Ph)_2_), 3.76 (s, 2H, NHC*H*_2_Ph), 3.87–3.96 (m, 1H, C*H*OH), 7.11–7.39 (m, 20H, Ph). ^13^C NMR (75.5 MHz, CDCl_3_), δ 32.6 (Ph*C*H_2_CH), 52.1 (CH_2_N), 53.3 (NH*C*H_2_Ph), 54.6 (N(*C*H_2_Ph)_2_), 62.2 (*C*HNBn_2_), 69.3 (CHOH), 125.7 (CH_Ph_), 126.8 (CH_Ph_), 127.3 (CH_Ph_), 128.1 (CH_Ph_), 128.2 (CH_Ph_), 128.3 (CH_Ph_), 128.5 (CH_Ph_), 128.8 (CH_Ph_), 129.6 (CH_Ph_), 139.0 (C_Ph_), 139.8 (C_Ph_), 141.4 (C_Ph_). NMR data were in agreement with those described in literature.^[27]^

### (2*R*,3*S*)‐1‐(Allylamino)‐3‐(dibenzylamino)‐4‐phenylbutan‐2‐ol (3 e)

From **1 e**: reduction of *anti* MAC product **1 e** (103 mg, 0.19 mmol) was performed with TMSCl (30 μL, 0.24 mmol) and LiAlH_4_ (270 μ.L, 0.27 mmol) in CH_2_Cl_2_ (1.5 mL). Flash chromatograp.hy gave *anti* DAPB **3 e** (44 mg, 57 %). From **2 e**: reduction of *anti* alcohol **2 e** (78 mg, 0.19 mmol) was performed with TMSCl (29 μL, 0.23 mmol) and LiAlH_4_ (265 μL, 0.26 mmol) in CH_2_Cl_2_ (1.5 mL). Flash chromatography gave *anti* DAPB **3 e** (35 mg, 46 %). Colorless oil. *R_f_* 0.22 (pentane/EtOAc, 1 : 2). [α]_D_^26^=−0.5 (*c* 1.0 in CHCl_3_). IR (ATR): ν 3360, 3079, 3061, 3024, 2926, 2849, 2803, 1601, 1494, 1453, 1367, 1275, 1260, 1114, 1072, 1026 cm^−1^. HRMS (ES^+^): calcd. for C_27_H_33_N_2_O [M+H]^+^ 401.2587; found 401.2574. ^1^H NMR (300 MHz, CDCl_3_), δ 2.51 (dd, *J*=12.1, 9.0 Hz, 1H) and 2.83–291 (m, 2H) (AB syst. CH_2_N, C*H*NBn_2_), 2.70 (br s, 2H, CHO*H*, NH), 2.99 (dd, *J*=14.2, 4.9 Hz, 1H) and 3.06 (dd, *J*=14.2, 8.1 Hz, 1H) (AB syst., PhC*H*_2_CH), 3.23 (br d, *J*=6.3 Hz, 2H, NC*H*_2_CH=CH_2_), 3.57 (d, *J*=13.7 Hz, 2H) and 3.69 (d, *J*=13.7 Hz, 2H) (AB syst., N(C*H*_2_Ph)_2_), 3.90–3.99 (m, 1H, C*H*OH), 5.12–5.24 (m, 2H, NCH_2_CH=C*H*_2_), 5.83 (ddt, *J*=16.7, 10.4, 6.3 Hz, 1H, NCH_2_C*H*=CH_2_), 7.12–7.32 (m, 15H, Ph). ^13^C NMR (75.5 MHz, CDCl_3_), δ 32.6 (Ph*C*H_2_CH), 51.5 (N*C*H_2_CH=CH_2_), 51.9 (CH_2_N) 54.5 (N(*C*H_2_Ph)_2_), 62.2 (*C*HNBn_2_), 68.9 (CHOH), 118.0 (NCH_2_CH=*C*H_2_), 125.8 (CH_Ph_), 126.9 (CH_Ph_), 128.2 (CH_Ph_), 128.3 (CH_Ph_), 128.9 (CH_Ph_), 129.6 (CH_Ph_), 134.4 (NCH_2_ *C*H=CH_2_) 139.7 (C_Ph_), 141.4 (C_Ph_).

### (2*R*,3*S*)‐3‐(Dibenzylamino)‐4‐phenyl‐1‐(prop‐2‐yn‐1‐ylamino)butan‐2‐ol (3 f)

From **1 f**: reduction of *anti* MAC product **1 f** (103 mg, 0.20 mmol) was performed with TMSCl (30 μL, 0.24 mmol) and LiAlH_4_ (270 μL, 0.27 mmol) in CH_2_Cl_2_ (1.5 mL). Flash chromatography gave *anti* DAPB **3 f** (30 mg, 40 %). From **2 f**: reduction of *anti* alcohol **2 f** (68 mg, 0.16 mmol) was performed with TMSCl (25 μL, 0.20 mmol) and LiAlH_4_ (230 μL, 0.23 mmol) in CH_2_Cl_2_ (1.2 mL). Flash chromatography gave *anti* DAPB **3 f** (32 mg, 49 %). Pale yellow oil. *R_f_* 0.13 (pentane/EtOAc, 1 : 2). [α]_D_^25^=−4.3 (*c* 1.0 in CHCl_3_). IR (ATR): ν 3406, 3299, 3084, 3061, 3028, 2928, 2842, 2804, 1603, 1494, 1450, 1372, 1252, 1110, 1072, 1026 cm^−1^. HRMS (ES^+^): calcd. for C_27_H_31_N_2_O [M+H]^+^ 399.2431; found 399.2413. ^1^H NMR (300 MHz, CDCl_3_), δ 2.04 (br s, 2H, CHO*H*, NH), 2.26 (t, *J*=2.3 Hz, 1H, NCH_2_C≡C*H*), 2.73 (dd, *J*=12.0, 8.2 Hz, 1H) and 2.84–3.05 (m, 3H) (AB syst. CH_2_N, C*H*NBn_2_, PhC*H*HCH), 3.1.2 (dd, *J*=13.9, 7.7 Hz, 1H, AB syst. PhCH*H*CH), 3.36 (d, *J*=2.0 Hz, 2H, NC*H*_2_C≡CH), 3.63 (d, *J*=13.7 Hz, 2H) and 3.75 (d, *J*=13.7 Hz, 2H) (AB syst., N(CH_2_Ph)_2_), 3.82–3.91 (m, 1H, C*H*OH), 7.19–7.36 (m, 15H, Ph). ^13^C NMR (75.5 MHz, CDCl_3_), δ 32.6 (Ph*C*H_2_CH), 37.9 (N*C*H_2_C≡CH), 51.6 (CH_2_N) 54.6 (N(*C*H_2_Ph)_2_), 62.0 (*C*HNBn_2_), 69.8 (CHOH), 71.5 (NCH_2_C≡*C*H), 81.9 (NCH_2_ *C*≡CH), 125.8 (CH_Ph_), 126.9 (CH_Ph_), 128.2 (CH_Ph_), 128.3 (CH_Ph_), 128.9 (CH_Ph_), 129.6 (CH_Ph_), 139.8 (C_Ph_), 141.4 (C_Ph_).

### (2*R*,3*S*)‐3‐(Dibenzylamino)‐1‐(isopropylamino)‐4‐phenylbutan‐2‐ol (3 g)

From **1 g**: reduction of *anti* MAC product **1 g** (80 mg, 0.15 mmol) was performed with TMSCl (23 μL, 0.18 mmol) and LiAlH_4_ (210 μL, 0.21 mmol) in CH_2_Cl_2_ (1.2 mL). Flash chromatography gave *anti* DAPB **3 g** (42 mg, 70 %). From **2 g**: reduction of *anti* alcohol **2 g** (59 mg, 0.14 mmol) was performed with TMSCl (22 μL, 0.17 mmol) and LiAlH_4_ (200 μL, 0.20 mmol) in CH_2_Cl_2_ (1.2 mL). Flash chromatography gave *anti* DAPB **3 c** (18 mg, 32 %). Colorless oil. *R_f_* 0.10 (pentan.e/EtOAc, 1 : 2). [α]_D_^25^=−1.8 (*c* 1.0 in CHCl_3_). IR (ATR.): ν 3317, 3089, 3062, 3026, 2967, 2921, 2844, 2803, 1601, 1491, 1449, 1403, 1365, 1119, 1095, 1072 cm^−1^. HRMS (ES^+^): calcd. for C_27_H_35_N_2_O [M+H]^+^ 403.2744; found 403.2732. ^1^H NMR (300 MHz, CDCl_3_), δ 1.10 (d, *J*=6.8 Hz, 3H, NCHC*H*_3_), 1.13 (d, *J*=6.8 Hz, 3H, NCHC*H*_3_), 2.45 (dd, *J*=12.1, 9.1 Hz, 1H) and 2.81–2.96 (m, 3H) (AB syst. CH_2_N, C*H*NBn_2_, NC*H*(CH_3_)_2_), 3.04 (dd, *J*=14.2, 7.2 Hz, 1H) and 3.10 (dd, *J*=14.2, 5.8 Hz, 1H) (AB syst., PhC*H*_2_CH), 3.60 (d, *J*=13.7 Hz, 2H) and 3.72 (d, *J*=13.7 Hz, 2H) (AB syst., N(C*H*_2_Ph)_2_), 3.94–4.13 (m, 3H, C*H*OH, CHO*H*, NH), 7.10–7.42 (m, 15H, Ph). ^13^C NMR (75.5 MHz, CDCl_3_), δ 21.5 (NCH*C*H_3_), 21.7 (NCH*C*H_3_), 32.7 (Ph*C*H_2_CH), 49.3 (N*C*H(CH_3_)_2_), 50.0 (CH_2_N), 54.5 (N(*C*H_2_Ph)_2_), 62.4 (*C*HNBn_2_), 68.8 (CHOH), 125.8 (CH_Ph_), 126.8 (CH_Ph_), 128.1 (CH_Ph_), 128.2 (CH_Ph_), 128.9 (CHPh), 129.6 (CH_Ph_), 139.8 (C_Ph_), 141.5 (C_Ph_).

### (2*R*,3*S*)‐1‐(Cyclopropylamino)‐3‐(dibenzylamino)‐4‐phenylbutan‐2‐ol (3 h)

From **1 h**: reduction of *anti* MAC product **1 h** (104. mg, 0.20 mmol) was performed with TMSCl (30 μL, 0.23 mmol) and LiAlH_4_ (280 μL, 0.28 mmol) in CH_2_Cl_2_ (1.5 mL). Flash chromatography gave *anti*‐DAPB **3 h** (44 mg, 55 %). From **2 h**: reduction of *anti* alcohol **2 h** (84 mg, 0.20 mmol) was performed with TMSCl (31 μL, 0.24 mmol) and LiAlH_4_ (280 μL, 0.28 mmol) in CH_2_Cl_2_ (1.5 mL). Flash chromatography gave *anti*‐DAPB **3 h** (25 mg, 31 %). Pale yellow oil. *R_f_* 0.22 (pentane/EtOAc, 1 : 2). [α]_D_^26^=−8.9 (*c* 0.98 in MeOH), lit.^[12]^ [α]_D_^25^=−7 (*c* 0.96 in MeOH). IR (ATR): ν 3415, 3300, 3084, 3061, 3024, 2923, 2844, 2798, 1601, 1491, 1450, 1367, 1104, 1072, 1021 cm^−1^. HRMS (ES^+^): calcd. for C_27_H_33_N_2_O [M+H]^+^ 401.2587; found 401.2591. ^1^H NMR (400 MHz, CDCl_3_), δ 0.24–0.38 (m, 2H, NCHC*H*_2_), 0.39–0.52 (m, 2H, NCHC*H*_2_), 2.05–2.13 (m, 1H, NC*H*(CH_2_)_2_), 2.41 (br s, 2H, CHO*H*, NH), 2.68 (dd, *J*=11.8, 8.6 Hz, 1H) and 2.84–2.94 (m, 2H) (AB syst., CH_2_N, C*H*NBn_2_), 3.00 (dd, *J*=14.1, 5.2 Hz, 1H) and 3.10 (dd, *J*=14.1, 8.2 Hz, 1H) (AB syst., PhC*H*_2_CH), 3.66 (d, *J*=13.6 Hz, 2H) and 3.75 (d, *J*=13.6 Hz, 2H) (AB syst., N(C*H*_2_Ph)_2_), 3.86–3.93 (m, 1H, C*H*OH), 7.19–7.44 (m, 15H, Ph). ^13^C NMR (100.6 MHz, CDCl_3_), δ 5.9 (NCH*C*H_2_), 6.8. (NCH*C*H_2_), 30.3 (N*C*H(CH_2_)_2_), 32.6 (Ph*C*H_2_CH), 52.8 (CH_2_N), 54.6 (N(*C*H_2_Ph)_2_), 62.0 (*C*HNBn_2_), 69.2 (CHOH), 125.7 (CH_Ph_), 126.8 (CH_Ph_), 12.8.1 (CH_Ph_), 128.2 (CH_Ph_), 128.8 (CH_Ph_), 129.6 (CH_Ph_), 139.9 (C_Ph_), 141.4 (C_Ph_). NMR data were in agreement with those described in literature.^[12]^

### (2*R*,3*S*)‐3‐(Dibenzylamino)‐1‐morpholino‐4‐phenylbutan‐2‐ol (3 i)

From **1 i**: reduction of *anti* MAC product **1 i** (82 mg, 0.15 mmol) was performed with TMSCl (22 μL, 0.17 mmol) and LiAlH_4_ (210 μL, 0.21 mmol) in CH_2_Cl_2_ (1.2 mL). Flash chromatography gave *anti* DAPB **3 i** (38 mg, 60 %). From **2 i**: reduction of *anti* alcohol **2 i** (56 mg, 0.13 mmol) was performed with TMSCl (19 μL, 0.15 mmol) and LiAlH_4_ (180 μL, 0.18 mmol) in CH_2_Cl_2_ (1 mL). Flash chromatography gave *anti* DAPB **3 i** (24 mg, 44 %). Pale yellow oil. *R_f_* 0.31 (pentane/EtOAc, 1 : 2). [α]_D_^23^=+5.6 (*c* 1.0 in CHCl_3_). IR (ATR): ν 3415, 3084, 3061, 3026, 2960, 2928, 2876, 2799, 1602, 1494, 1453, 1364, 1143, 1074, 1028 cm^−1^. HRMS (ES^+^): calcd. for C_28_H_35_N_2_O_2_ [M+H]^+^ 431.2693; found 431.2673. ^1^H NMR (400 MHz, CDCl_3_), δ 2.09 (br dd, *J*=12.2, 11.0 Hz, 1H) and 2.44 (dd, *J*=12.2, 3.5 Hz, 1H) (AB syst., CH_2_N), 2.28–2.36 (m, 2H) and 2.60–2.68 (m, 2H) (AB syst., N(C*H*_2_CH_2_)_2_O), 2.85–2.90 (m, 1H, C*H*NBn_2_), 2.99 (dd, *J*=14.3, 5.2 Hz, 1H) and 3.10 (dd, *J*=14.3, 8.2 . Hz, 1H) (AB syst., PhC*H*_2_CH), 3.64–3.72 (m, 4H, (N(CH_2_C*H*_2_)_2_O), 3.72 (s, 4H, N(C*H*_2_Ph)_2_), 4.10 (br ddd, *J*=11.0, 3.5, 3.5 Hz, 1H, C*H*OH), 7.16–7.35 (m, 15H, Ph) [CHO*H* signal not observed]. ^13^C NMR (100.6 MHz, CDCl_3_), δ 32.3 (Ph*C*H_2_CH), 53.4 (N(*C*H_2_CH_2_)_2_O), 54.6 (N(*C*H_2_Ph)_2_), 62.3 (*C*HNBn_2_), 62.9 (CH_2_N), 65.7 (CHOH), 67.0 (N(CH_2_ *C*H_2_)_2_O), 125.7 (CH_Ph_), 126.8 (CH_Ph_), 128.1 (CH_Ph_), 128.7 (CH_Ph_), 129.6 (CH_Ph_), 139.9 (C_Ph_), 141.4 (C_Ph_).

### (2*R*,3*S*)‐3‐(Dibenzylamino)‐4‐phenyl‐1‐(pyrrolidin‐1‐yl)butan‐2‐ol (3 j)

From **1 j**: reduction of *anti* MAC product **1 j** (82 mg, 0.15 mmol) was performed with TMSCl (23 μL, 0.18 mmol) and LiAlH_4_ (210 μL, 0.21 mmol) in CH_2_Cl_2_ (1.2 mL). Flash chromatography gave *anti* DAPB **3 j** (51 mg, 81 %). From **2 j**: reduction of *anti* alcohol **2 j** (52 mg, 0.12 mmol) was performed with TMSCl (19 μL, 0.15 mmol) and LiAlH_4_ (170 μL, 0.17 mmol) in CH_2_Cl_2_ (1 mL). Flash chromatography gave *anti* DAPB **3 j** (27 mg, 54 %). Pale yellow oil. *R_f_* 0.13 (pentane/.EtOAc, 1 : 2). [α]_D_^23^=+5.1 (*c* 1.0 in CHCl_3_). IR (ATR): ν 3444, 3084, 3061, 3026, 2963, 2926, 2852, 2807, 1602, 1495, 1453, 1369, 1294, 1118, 1071, 102.8 cm^−1^. HRMS (ES^+^): calcd. for C_28_H_35_N_2_O [M+H]^+^ 415.2744; found 415.2727. ^1^H NMR (300 MHz, CDCl_3_), δ 1.76–1.90 (m, 4H, N(CH_2_C*H*_2_)_2_), 2.44 (dd, *J*=12.0, 4.0 Hz, 1H) and 2.47–2.58 (m, 3H) (AB syst. CH_2_N, NC*H*_2_CH_2_), 2.70–2.80 (m, 2H, AB syst., NC*H*_2_CH_2_), 2.87–2.94 (m, 1H, C*H*NBn_2_), 3.02 (dd, *J*=14.3, 4.7 Hz, 1H) and 3.12 (dd, *J*=14.3, 8.6 Hz, 1H) (AB syst., PhC*H*_2_CH), 3.72 (d, *J*=14.0 Hz, 2H) and 3.80 (d, *J*=14.0 Hz, 2H) (AB syst., N(C*H*_2_Ph)_2_), 4.08 (ddd, *J*=10.6, 4.2, 4.0 Hz, 1H, C*H*OH), 4.33 (br s, 1H, CHO*H*), 7.16–7.36 (m, 15H, Ph). ^13^C NMR (75.5 MHz, CDCl_3_), δ 23.5 (N(CH_2_ *C*H_2_)_2_), 32.4 (Ph*C*H_2_CH), 53.8 (N(*C*H_2_CH_2_)_2_), 54.5 (N(*C*H_2_Ph)_2_), 60.4 (CH_2_N), 62.4 (*C*HNBn_2_), 67.3 (CHOH), 125.6 (CH_Ph_), 126.6 (CH_Ph_), 128.0 (CH_Ph_), 128.7 (CH_Ph_), 129.6 (CH_Ph_), 140.0 (C_Ph_), 141.4 (C_Ph_).

## Conflict of Interests

The authors declare no conflict of interest.

1

## Supporting information

As a service to our authors and readers, this journal provides supporting information supplied by the authors. Such materials are peer reviewed and may be re‐organized for online delivery, but are not copy‐edited or typeset. Technical support issues arising from supporting information (other than missing files) should be addressed to the authors.

Supporting Information

## Data Availability

The data that support the findings of this study are available in the supplementary material of this article.
