# Supplementary material for: Rapid Synthesis of anti‐1,3‐Diamino‐4‐phenylbutan‐2‐ol Building Blocks via a Three‐Component Oxyhomologation and a Two‐Component Reducing System
Source: ChemistryOpen. 2024 Oct 30;14(2):e202400279. doi: 10.1002/open.202400279 (PMC12128162; doi:10.1002/open.202400279)
Supplement: Supplementary file 1 — Supporting Information [file OPEN-14-e202400279-s001.pdf]

ChemistryOpen

Supporting Information

Rapid Synthesis of *anti*-1,3-Diamino-4-phenylbutan-2-ol Building Blocks via a Three-Component Oxyhomologation and a Two-Component Reducing System

Maria Chiara Cabua, Xuefeng He, Francesco Secci, Sandrine Deloisy,* and David J. Aitken*

Supporting Information

Copies of ^{1}H and ^{13}C NMR spectra S2

Crystallographic data for compound **1c** S24

Copies of ^{1}H and ^{13}C NMR spectra.

(2*S*,3*S*)-*N*-(3-phenylpropyl)-2-(*tert*-butyldimethylsilyloxy)-3-(dibenzylamino)-4-phenylbutanamide (**1c**)

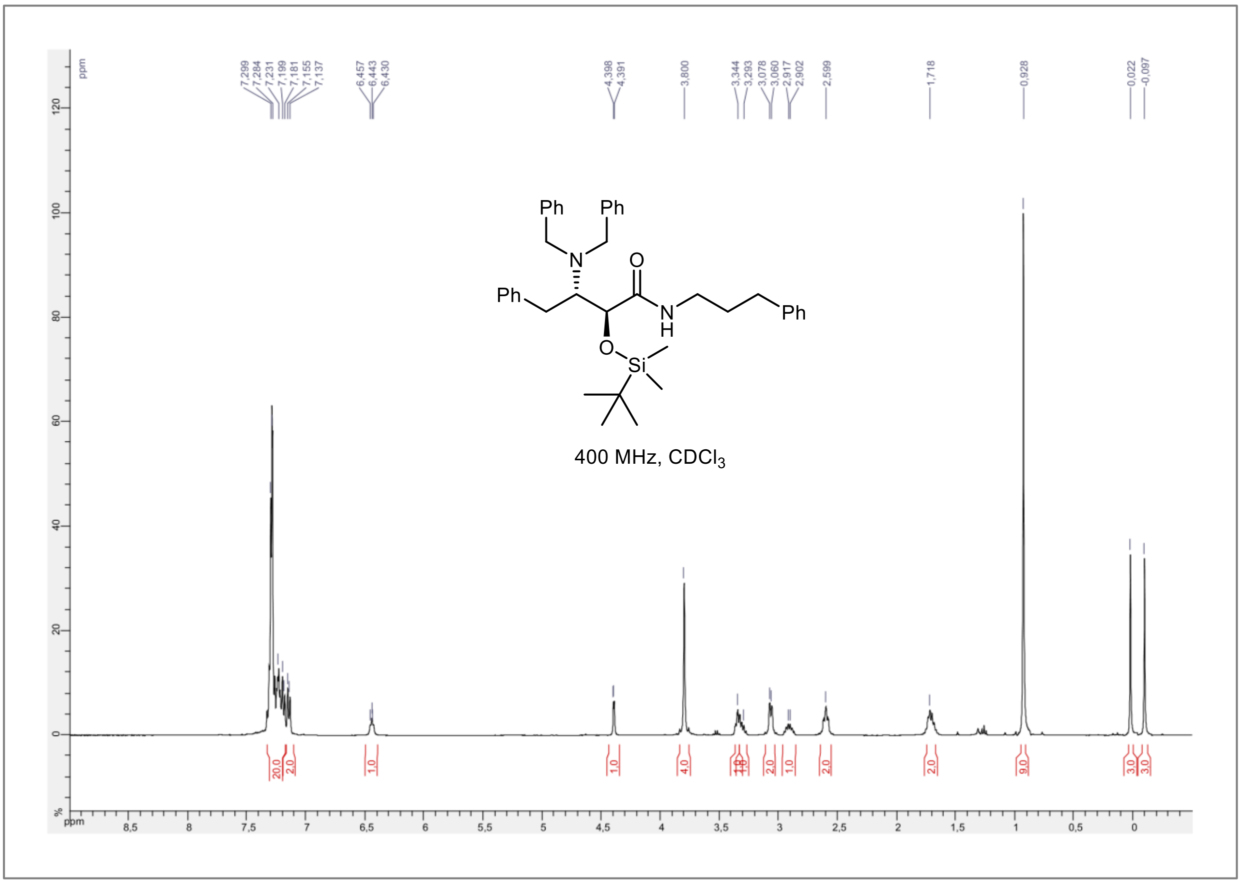

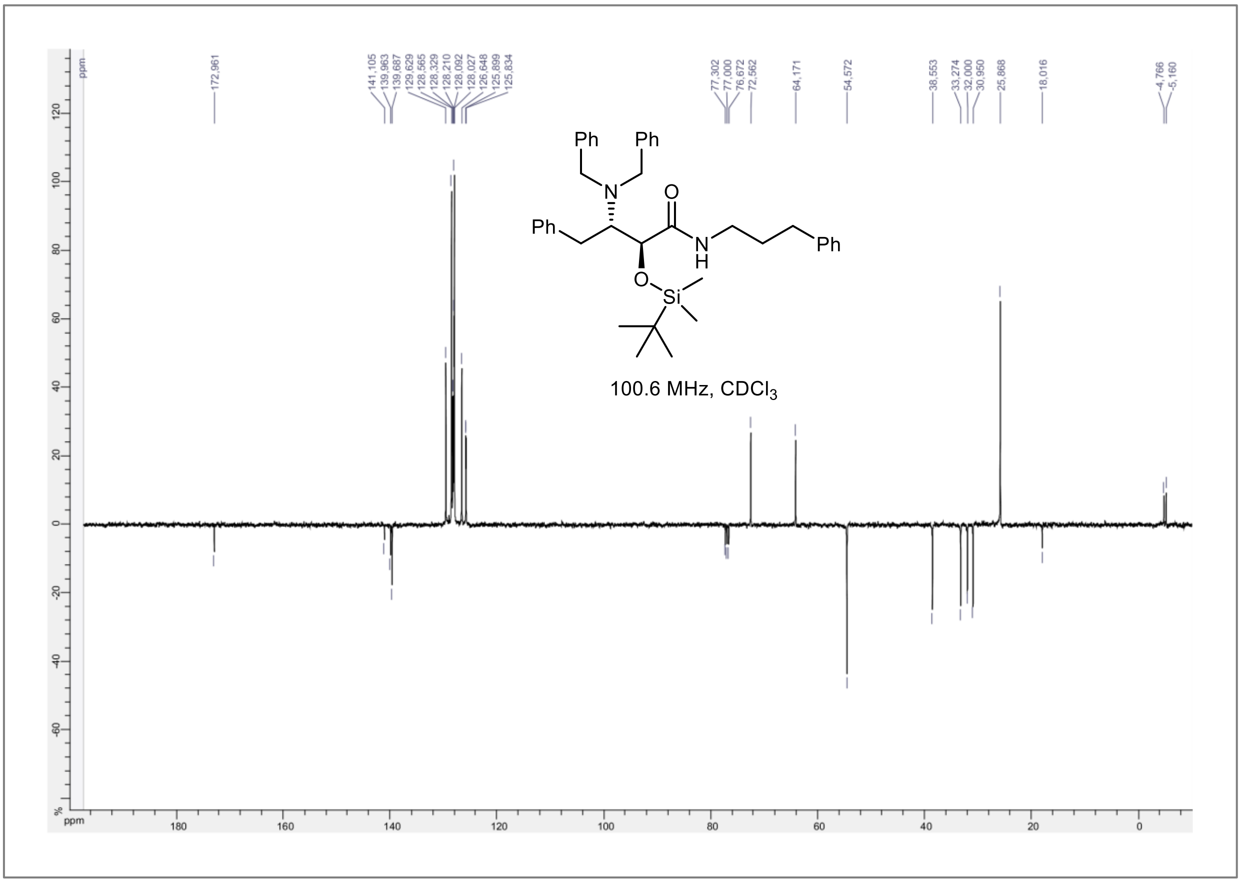

(2*S*,3*S*)-*N*-Allyl-2-(*tert*-butyldimethylsilyloxy)-3-(dibenzylamino)-4-phenylbutanamide (**1e**)

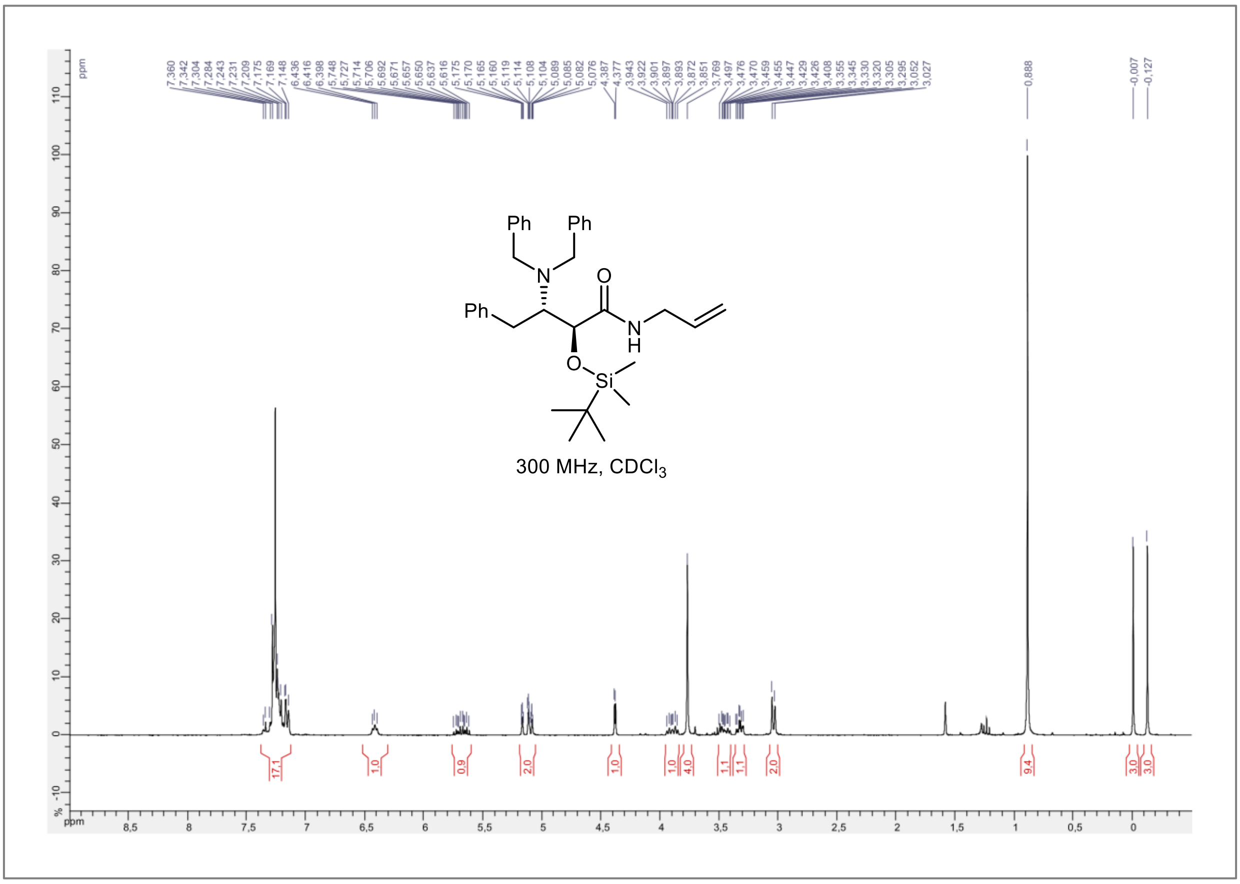

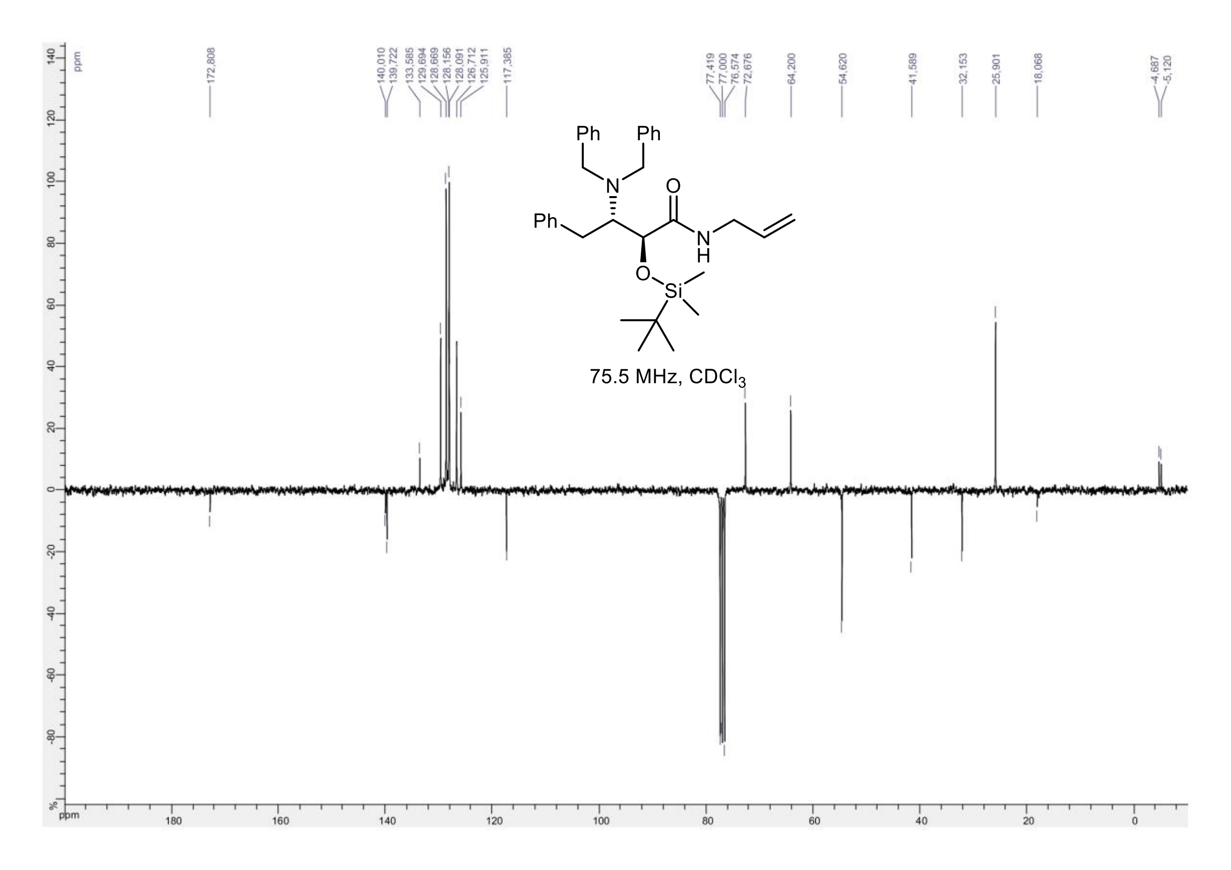

(2*S*,3*S*)-*N*-Isobutyl-3-(dibenzylamino)-2-hydroxy-4-phenylbutanamide (**2a**)

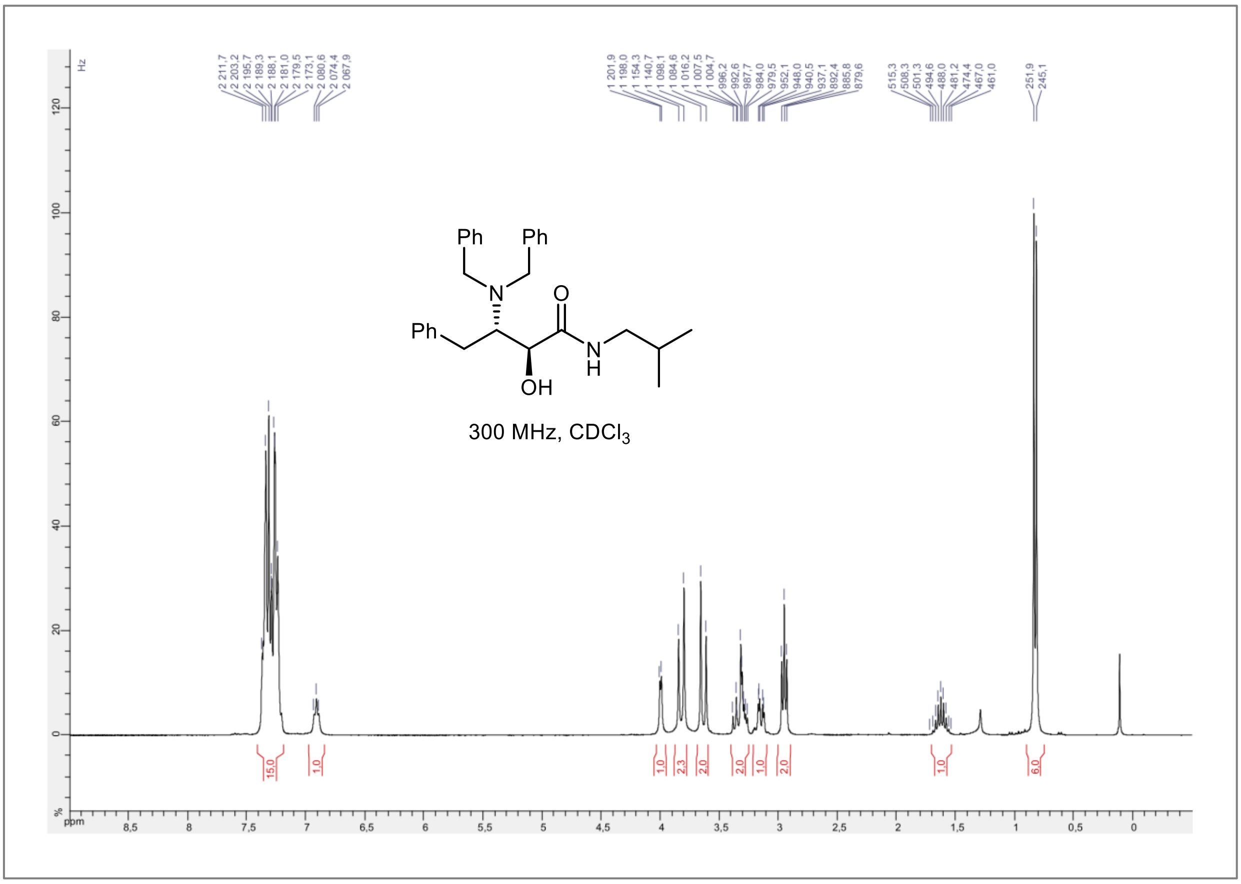

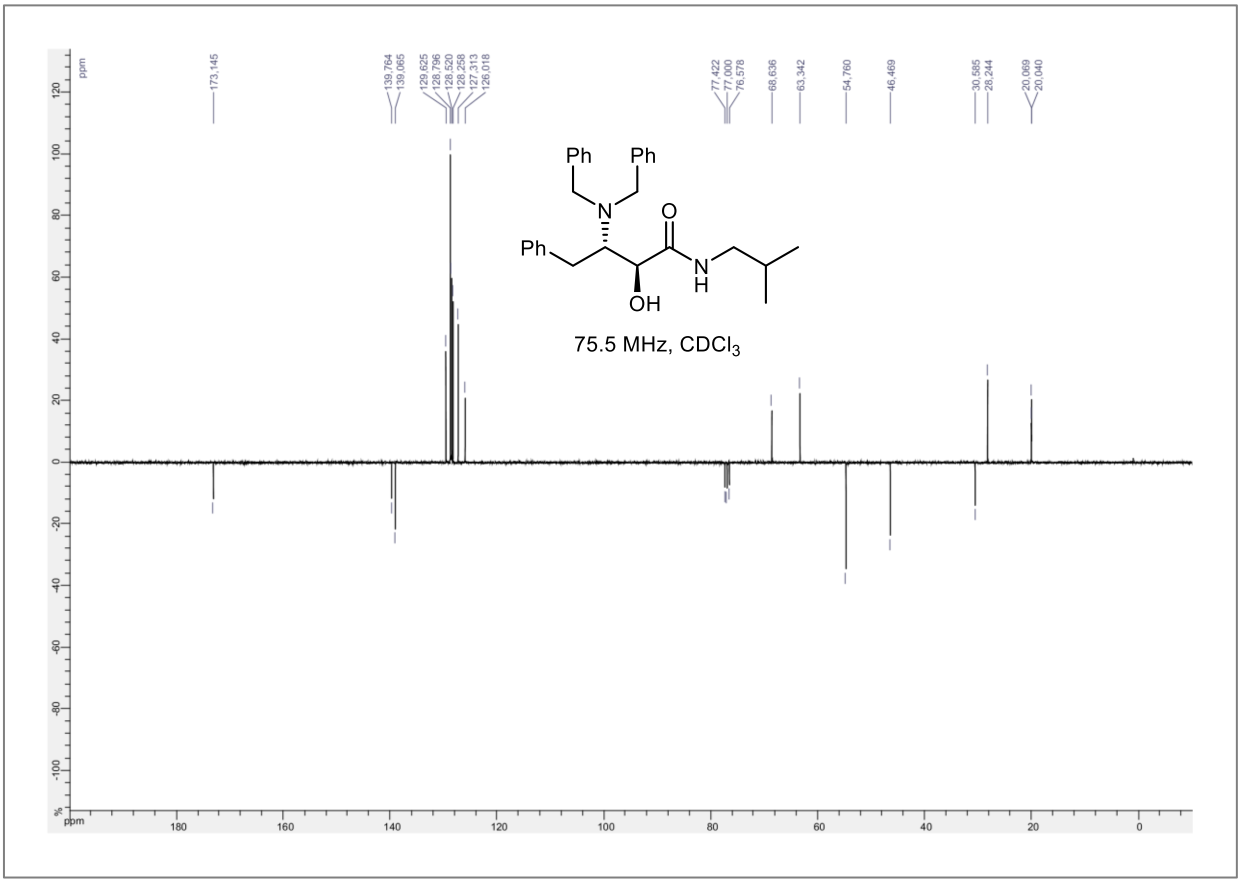

(2*S*,3*S*)-*N*-Butyl-3-(dibenzylamino)-2-hydroxy-4-phenylbutanamide (**2b**)

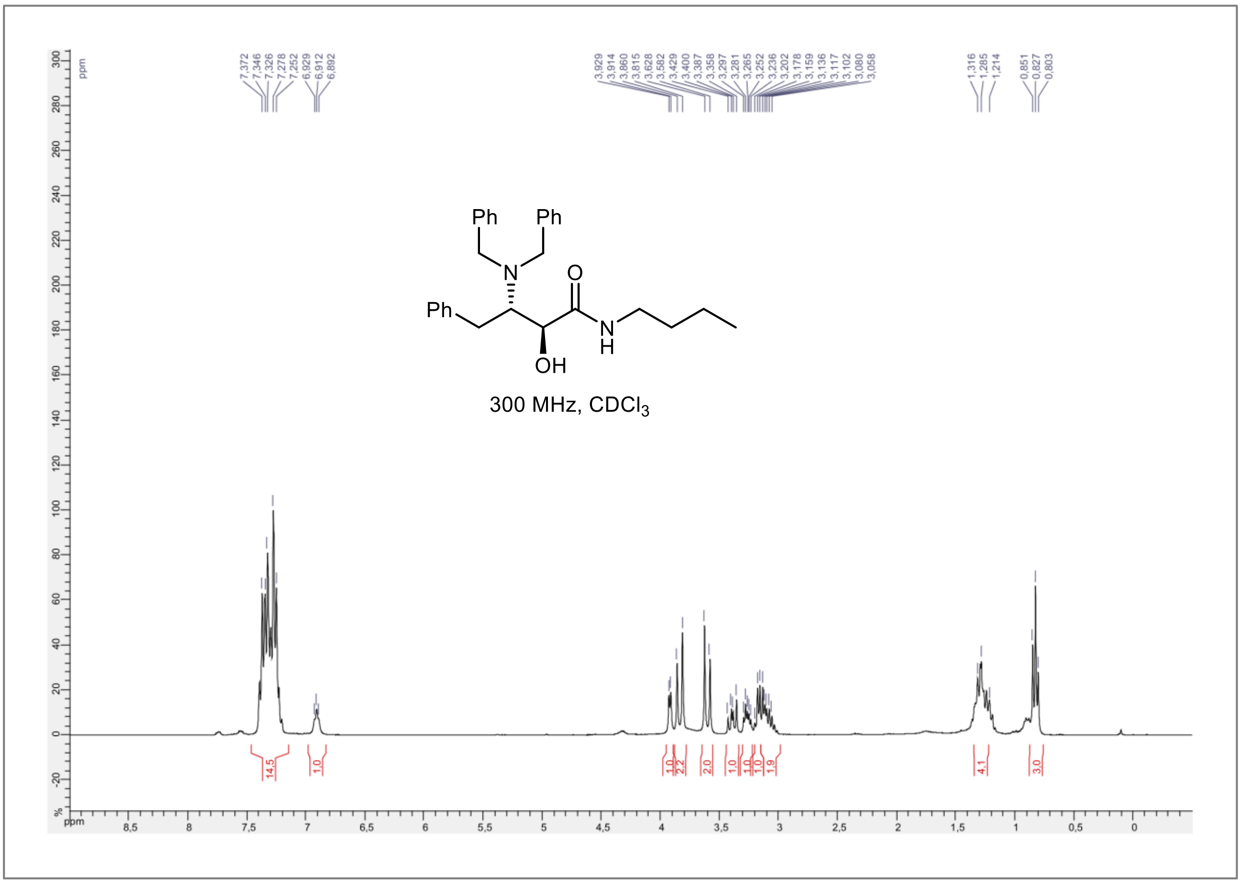

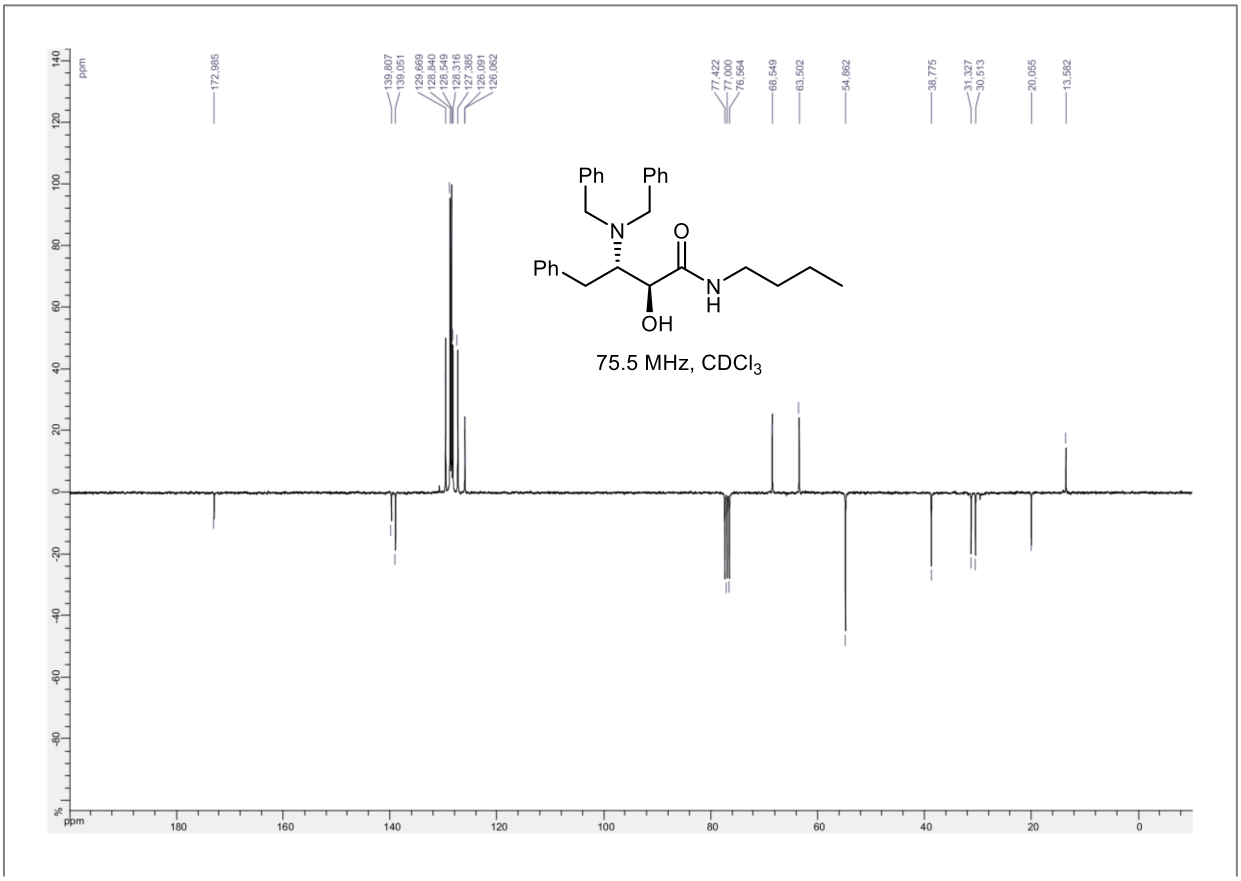

(2*S*,3*S*)-*N*-(3-phenylpropyl)-3-(dibenzylamino)-2-hydroxy-4-phenylbutanamide (**2c**)

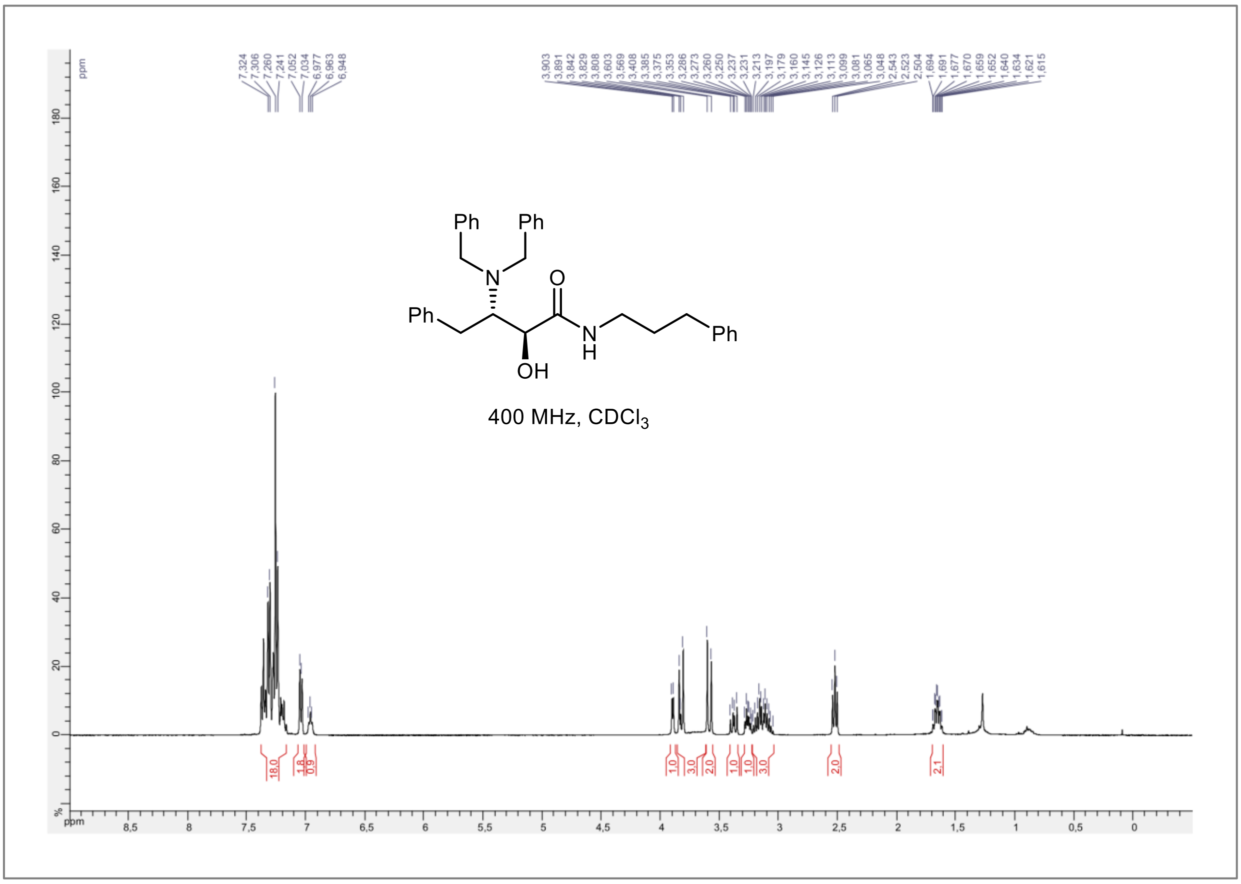

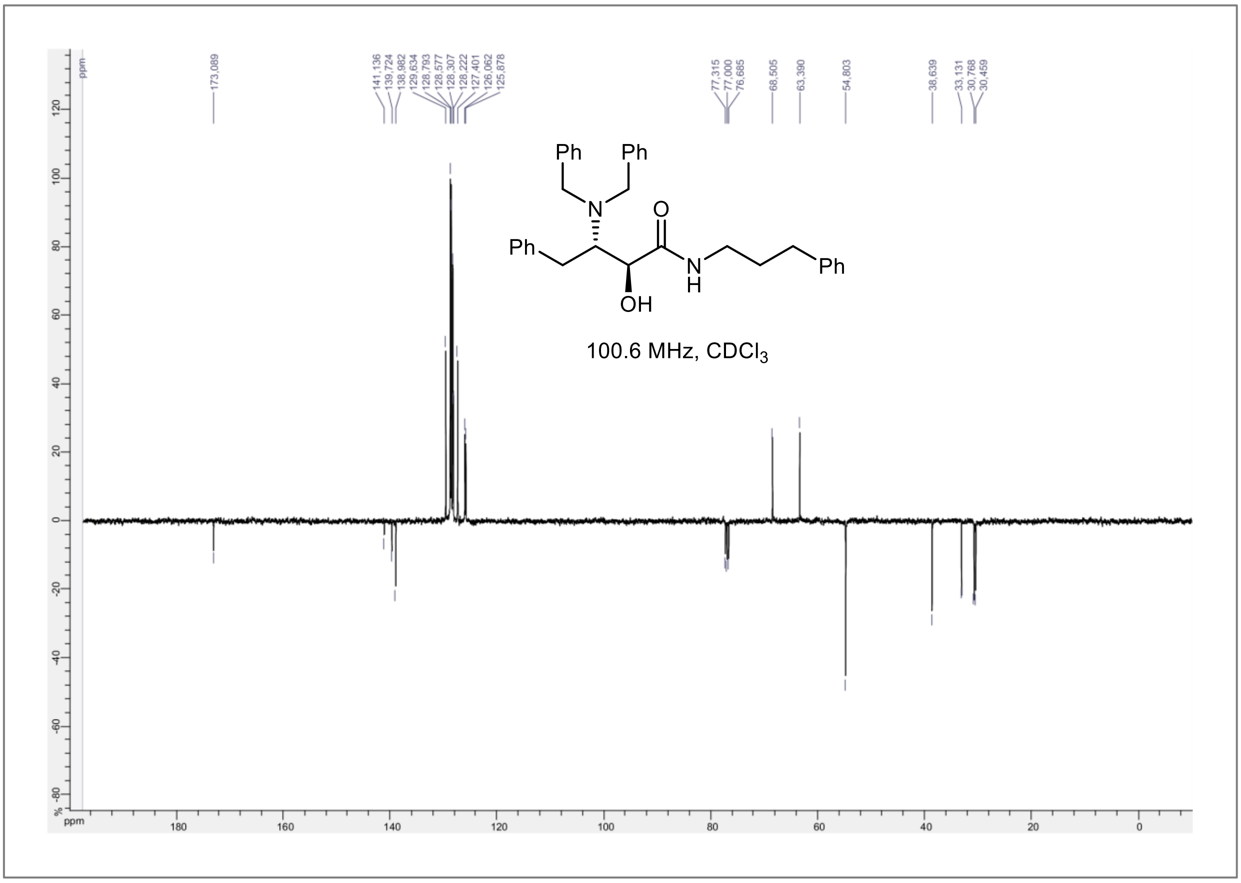

(2*S*,3*S*)-*N*-Benzyl-3-(dibenzylamino)-2-hydroxy-4-phenylbutanamide (**2d**)

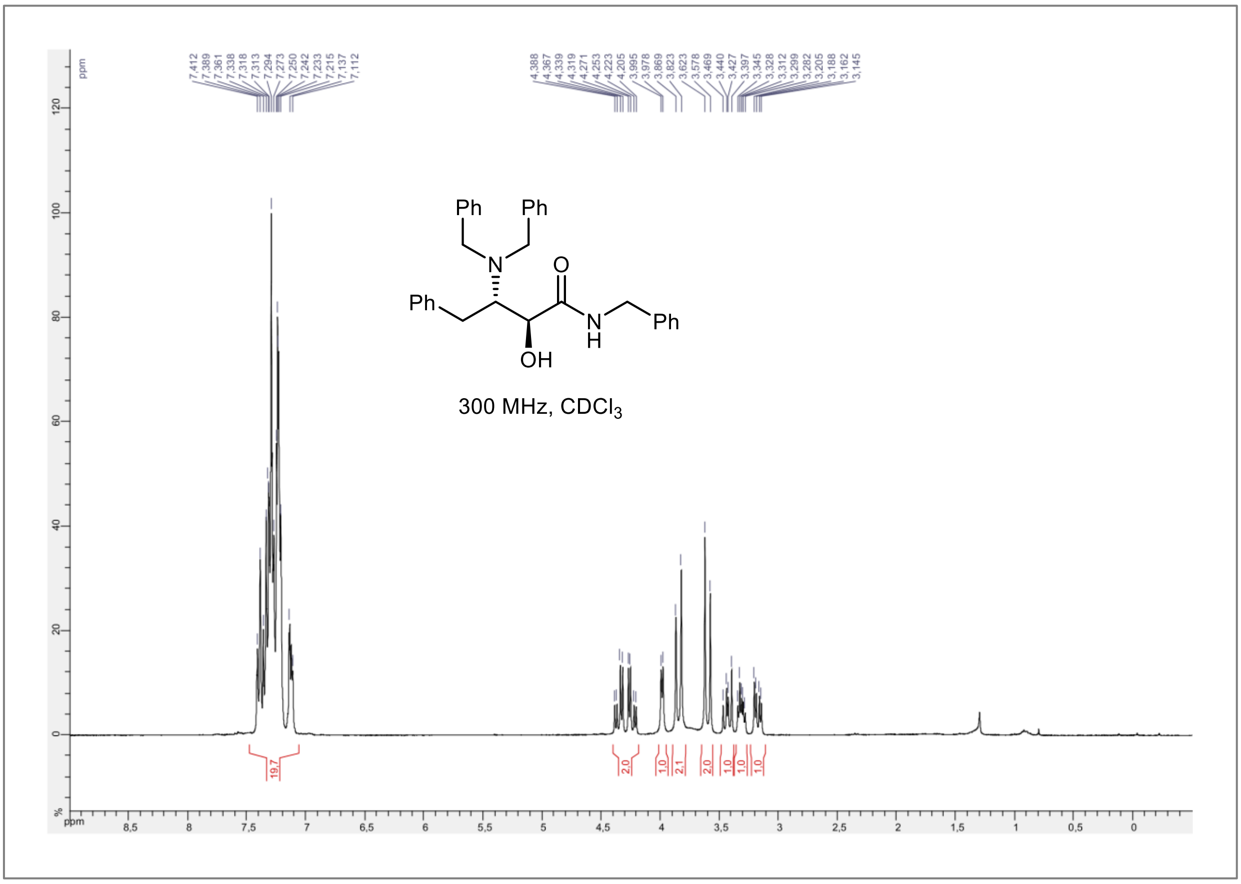

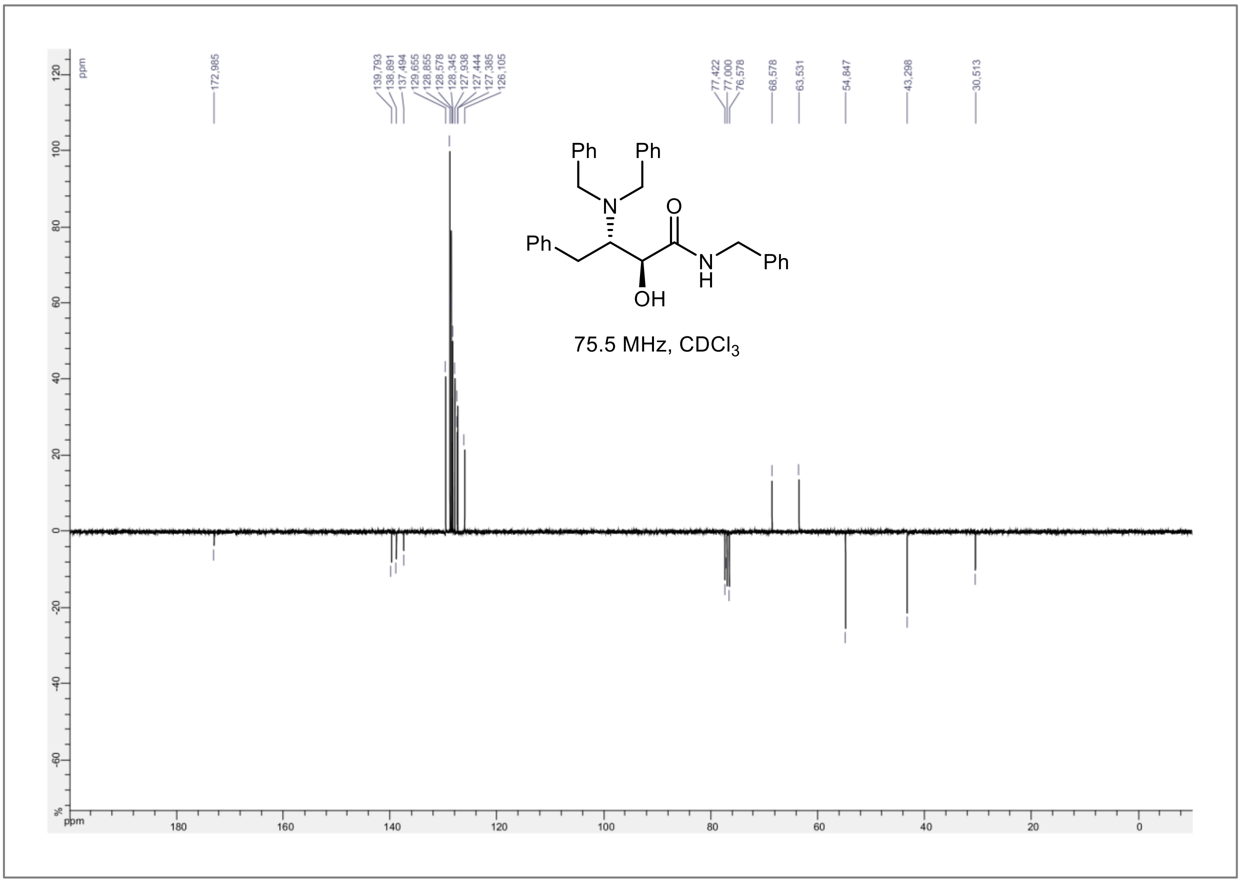

(2*S*,3*S*)-*N*-Allyl-3-(dibenzylamino)-2-hydroxy-4-phenylbutanamide (**2e**)

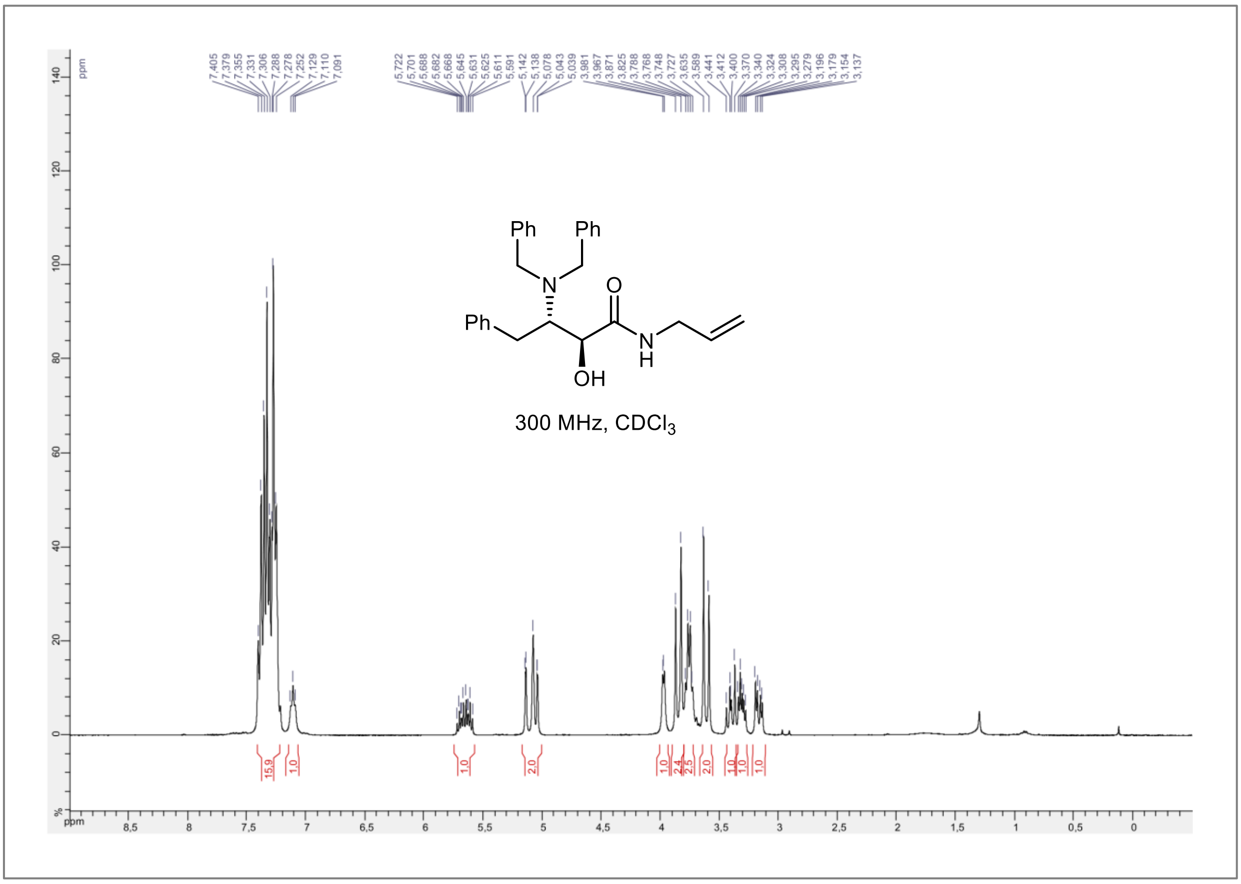

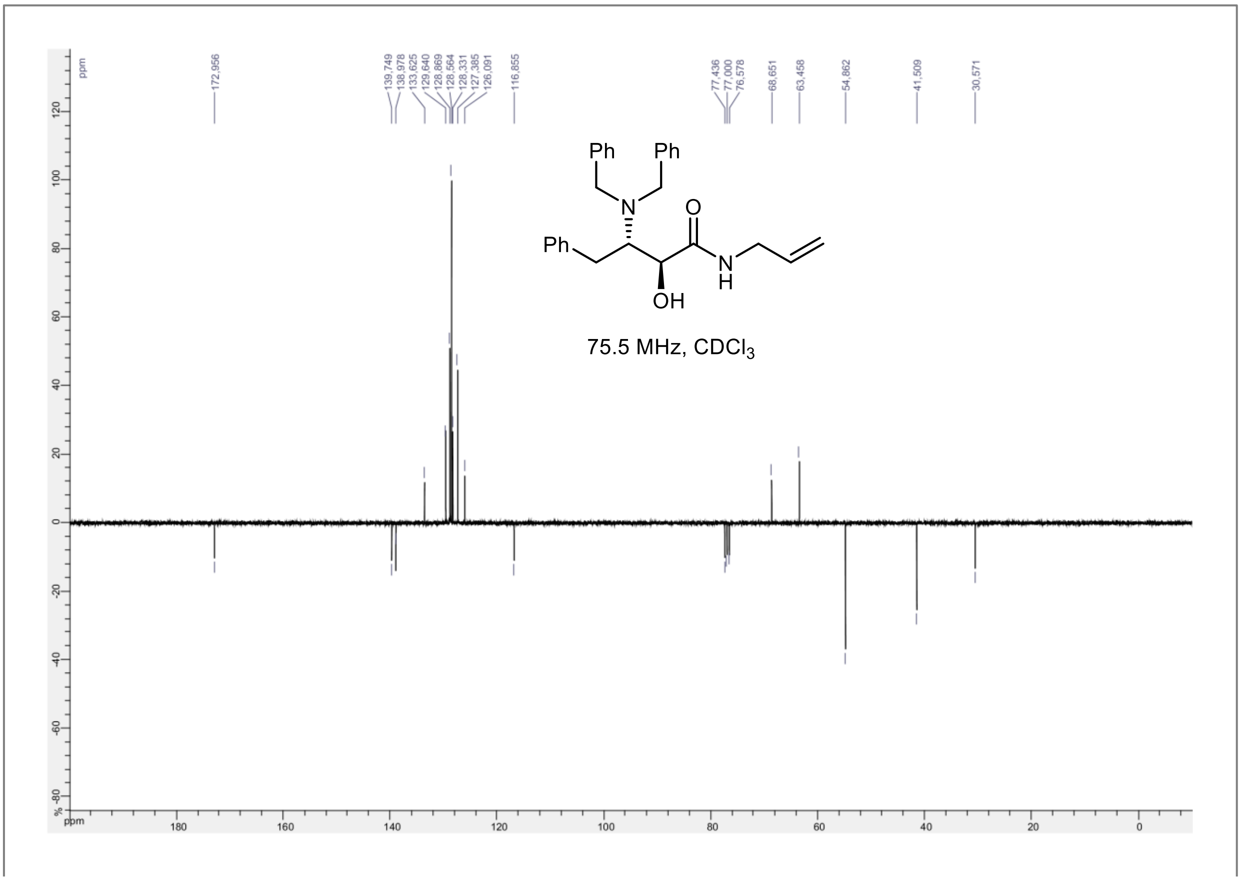

(2*S*,3*S*)-*N*-(Prop-2-yn-1-yl)-3-(dibenzylamino)-2-hydroxy-4-phenylbutanamide (**2f**)

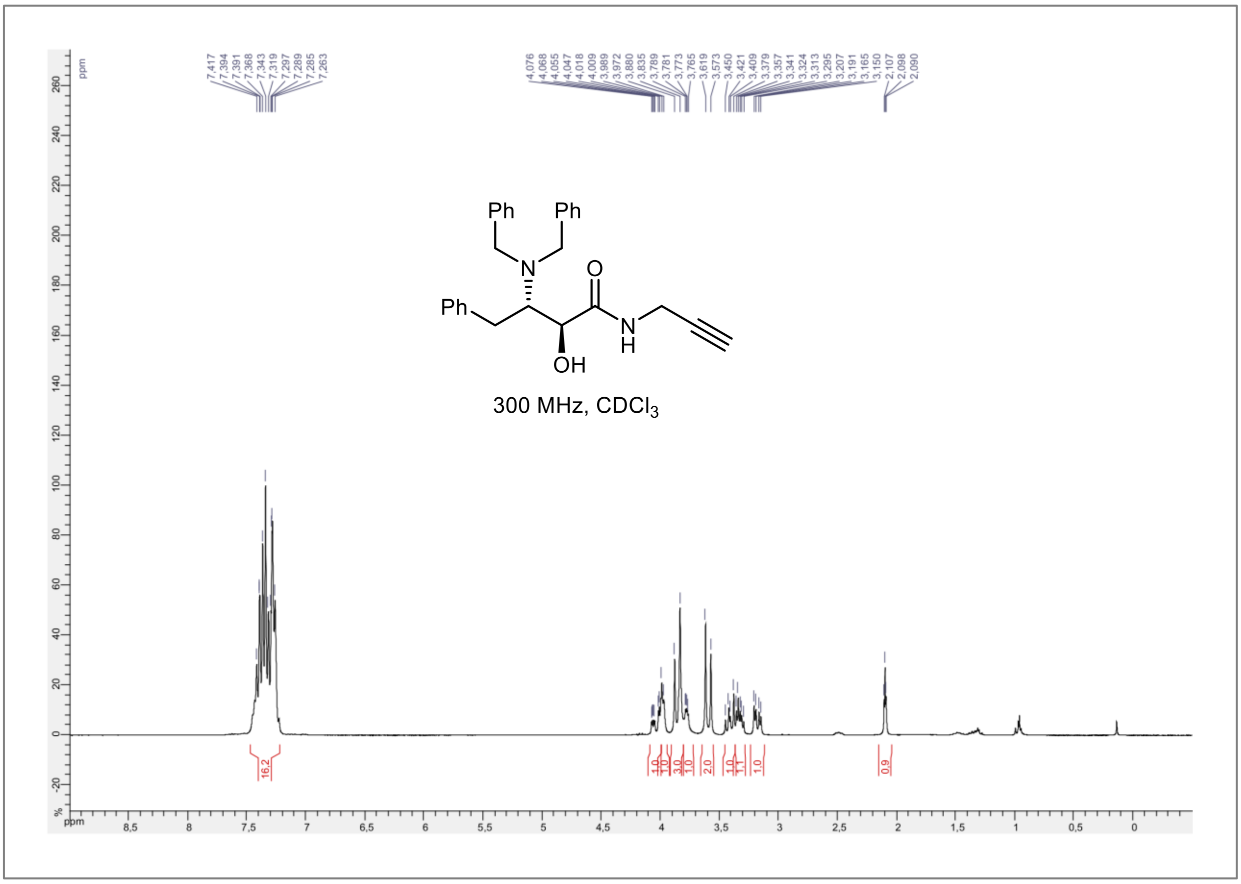

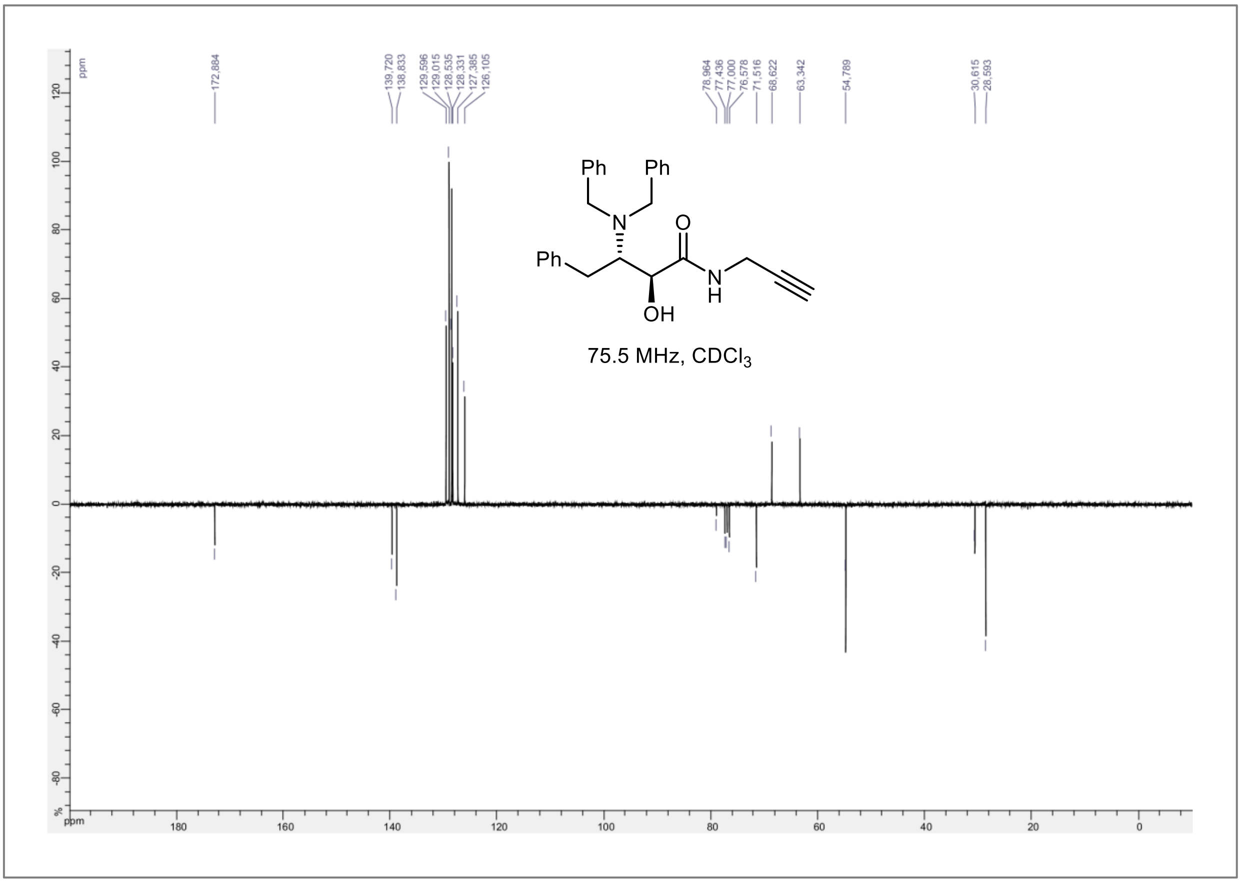

(2*S*,3*S*)-*N*-Isopropyl-3-(dibenzylamino)-2-hydroxy-4-phenylbutanamide (**2g**)

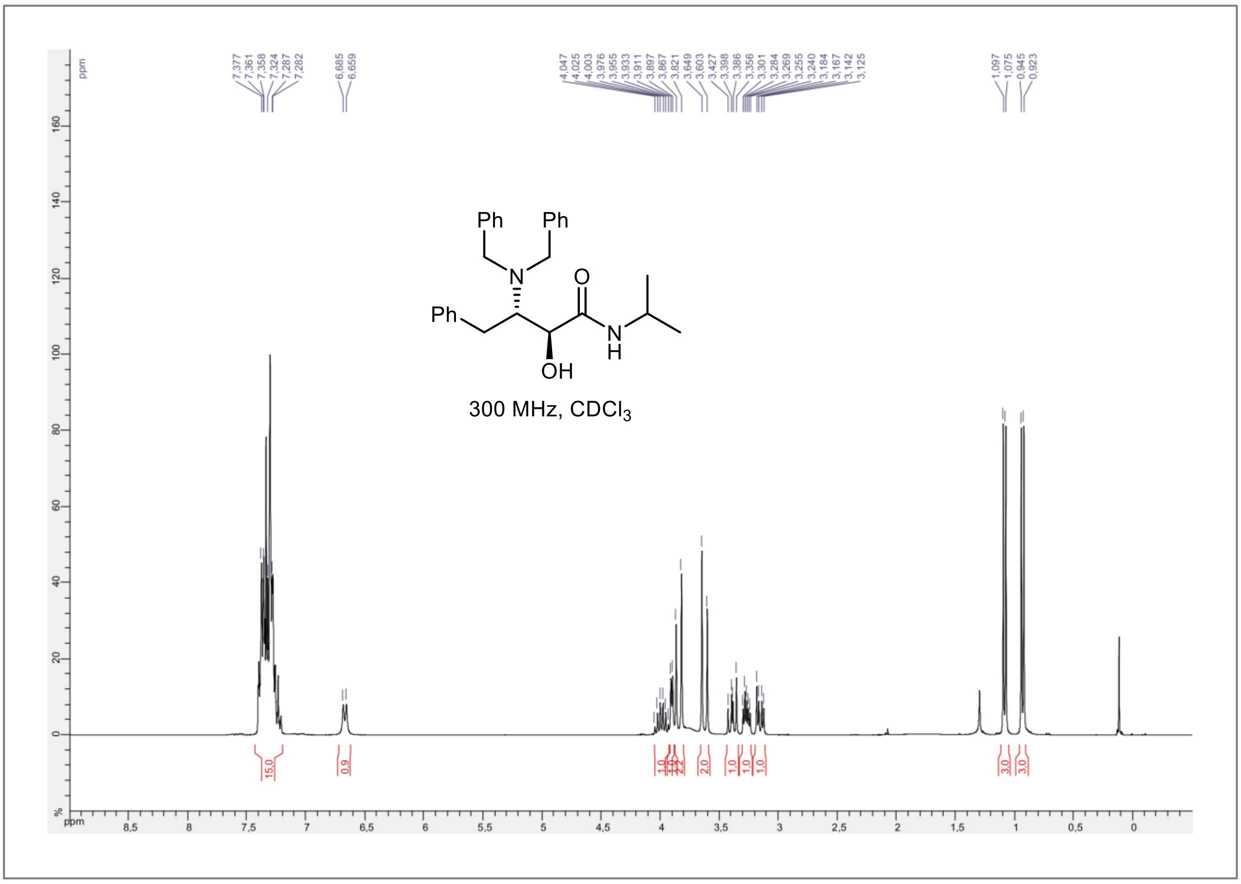

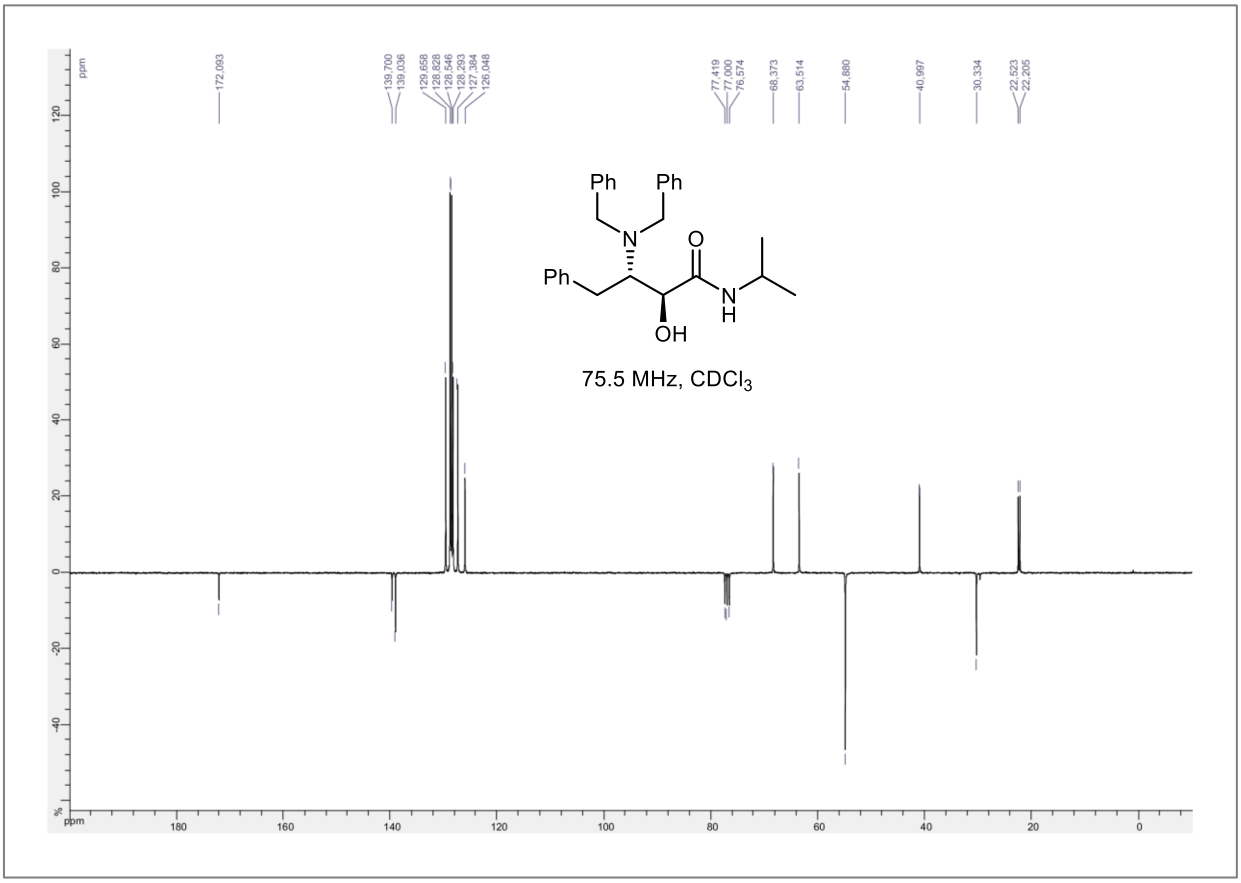

(2*S*,3*S*)-*N*-Cyclopropyl-3-(dibenzylamino)-2-hydroxy-4-phenylbutanamide (**2h**)

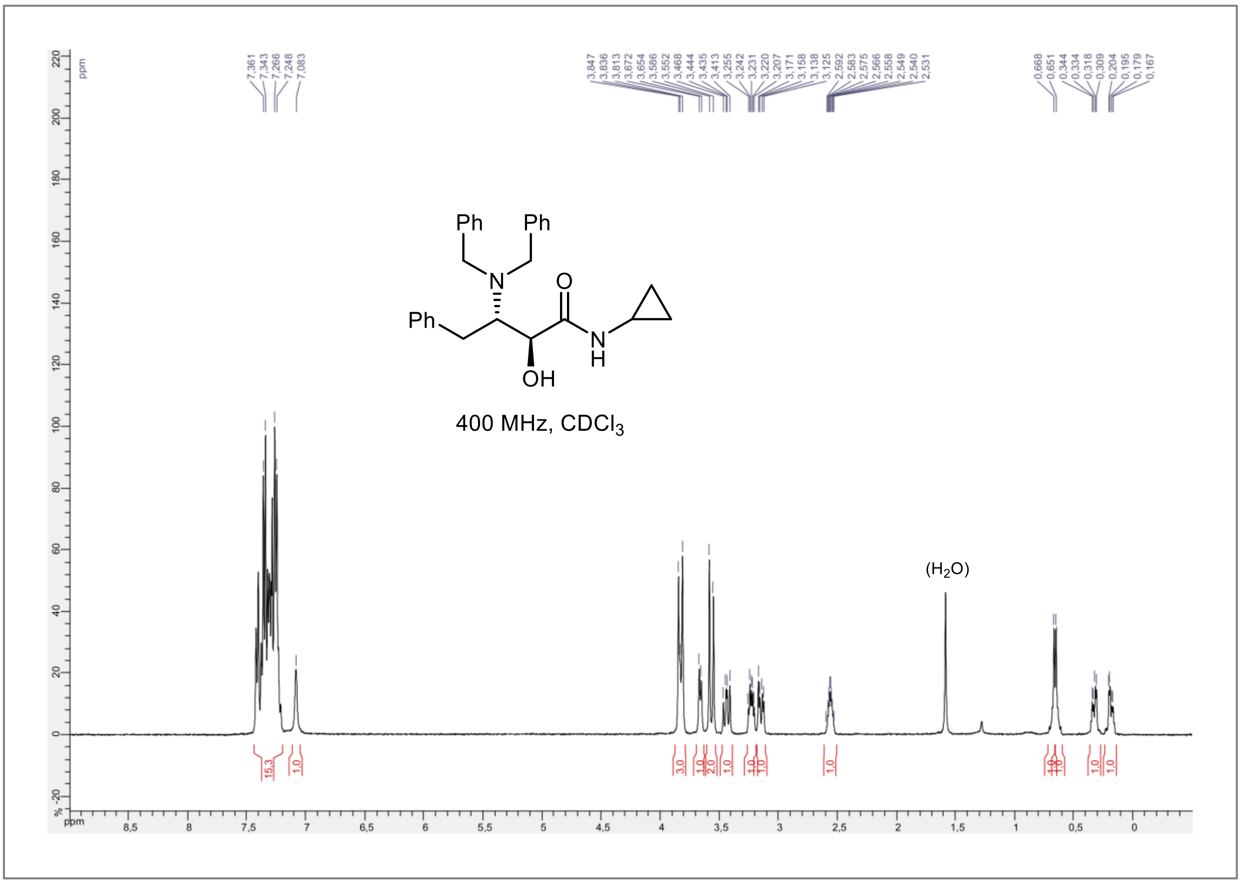

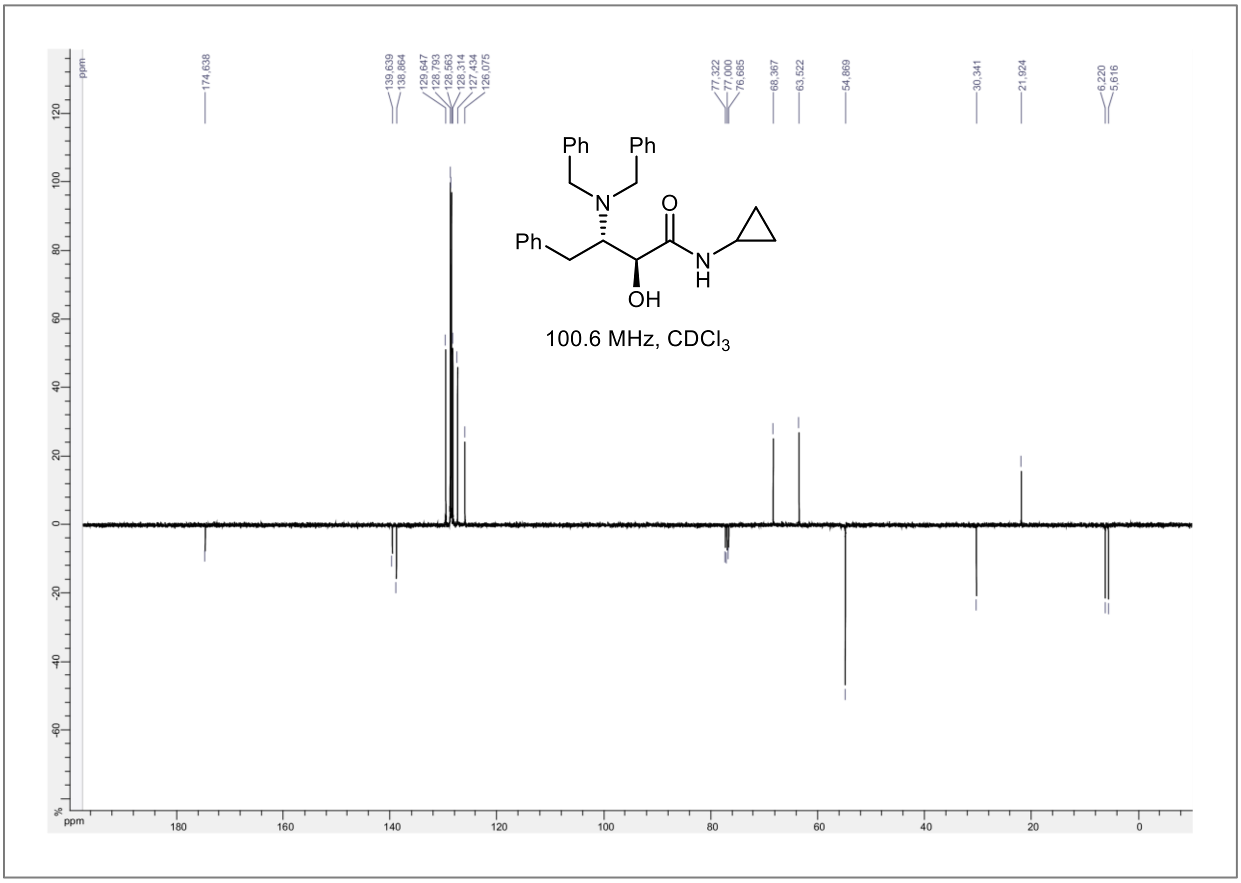

(2*S*,3*S*)-3-(Dibenzylamino)-2-hydroxy-1-morpholino-4-phenylbutan-1-one (**2i**)

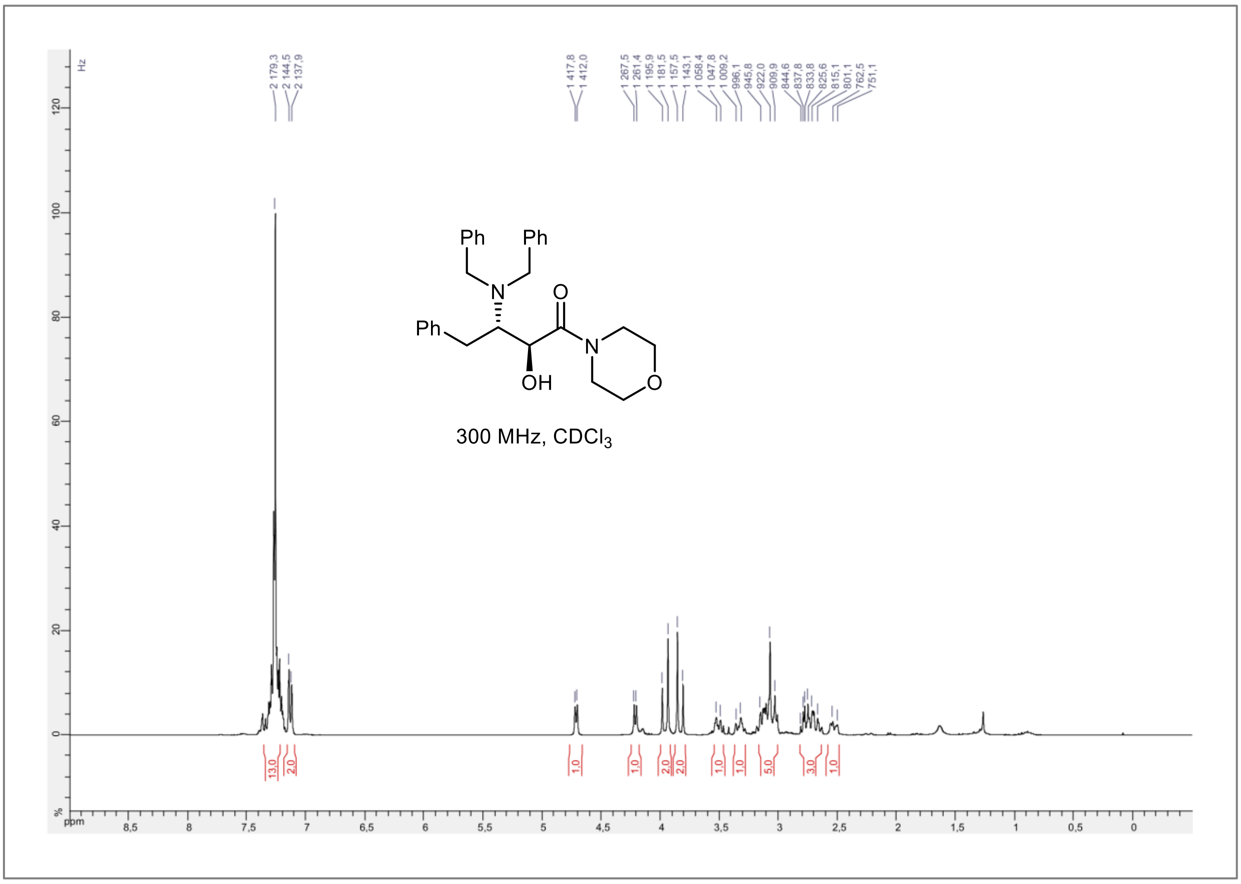

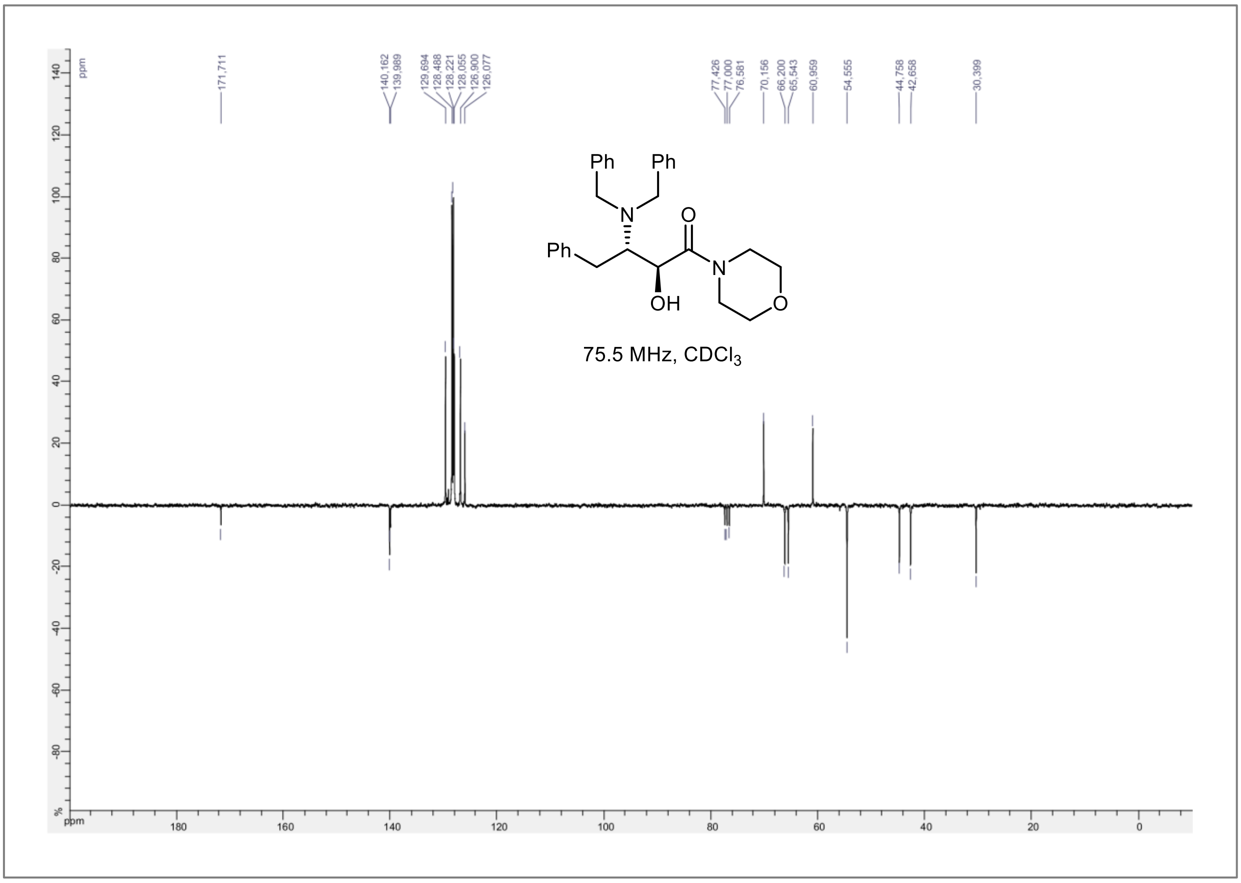

(2*S*,3*S*)-3-(Dibenzylamino)-2-hydroxy-4-phenyl-1-(pyrrolidin-1-yl)butan-1-one (**2j**)

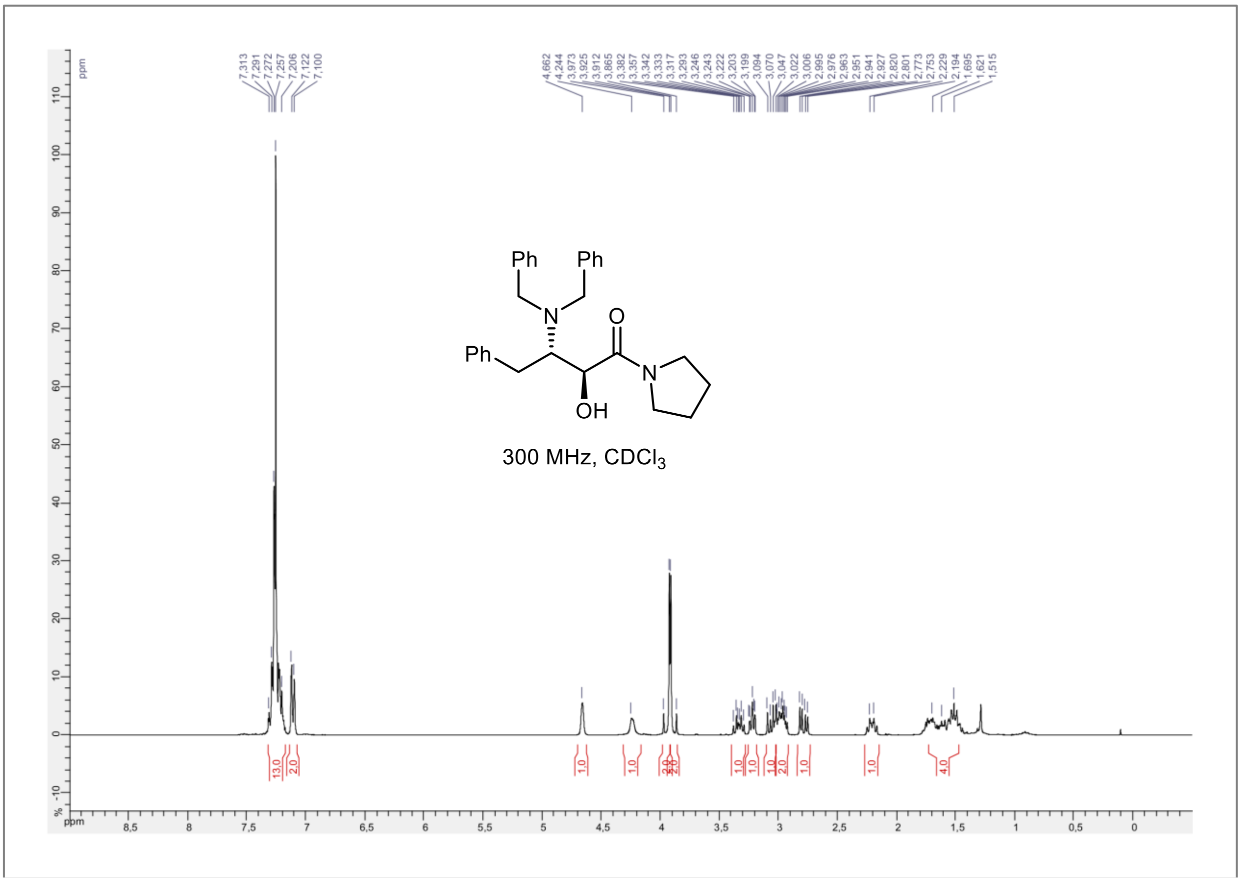

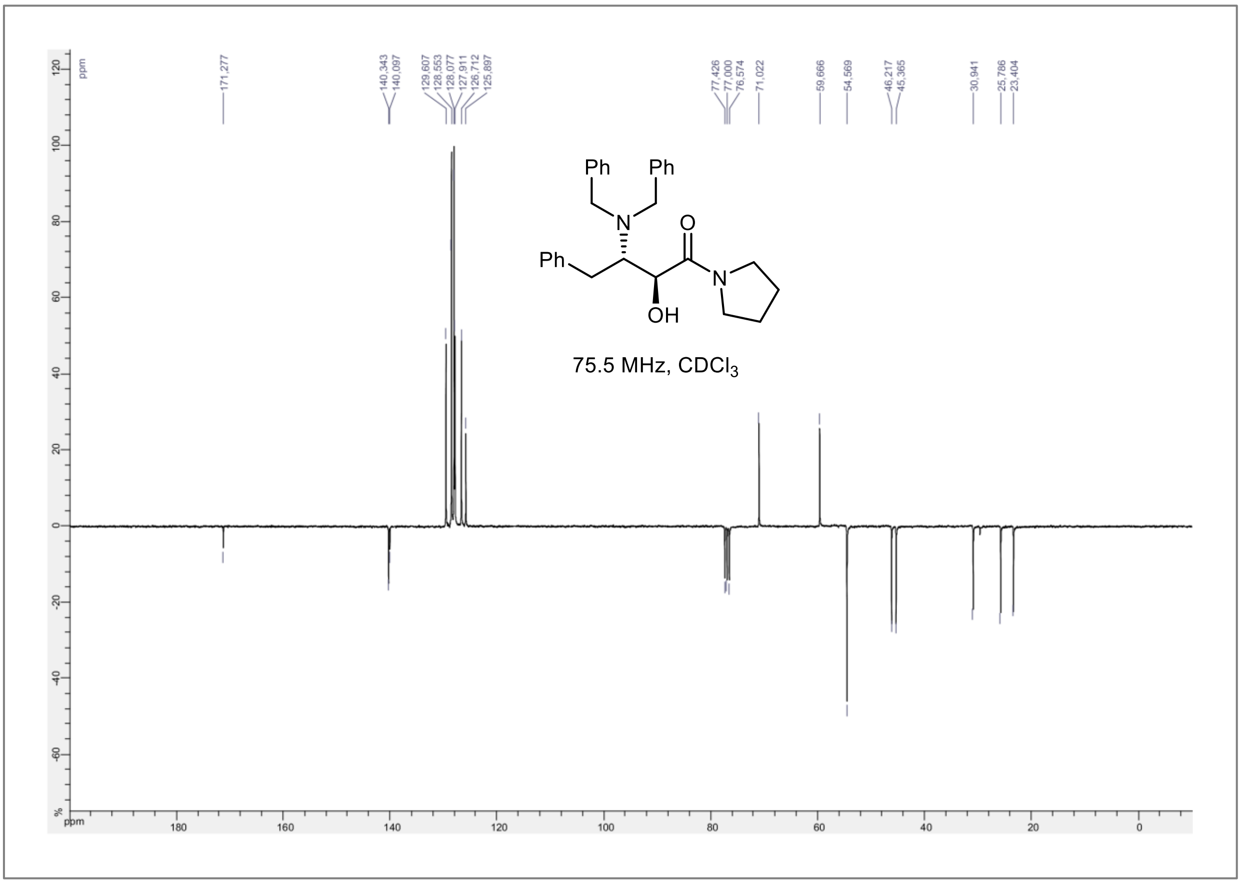

(2*R*,3*S*)-3-(Dibenzylamino)-1-(isobutylamino)-4-phenylbutan-2-ol (**3a**)

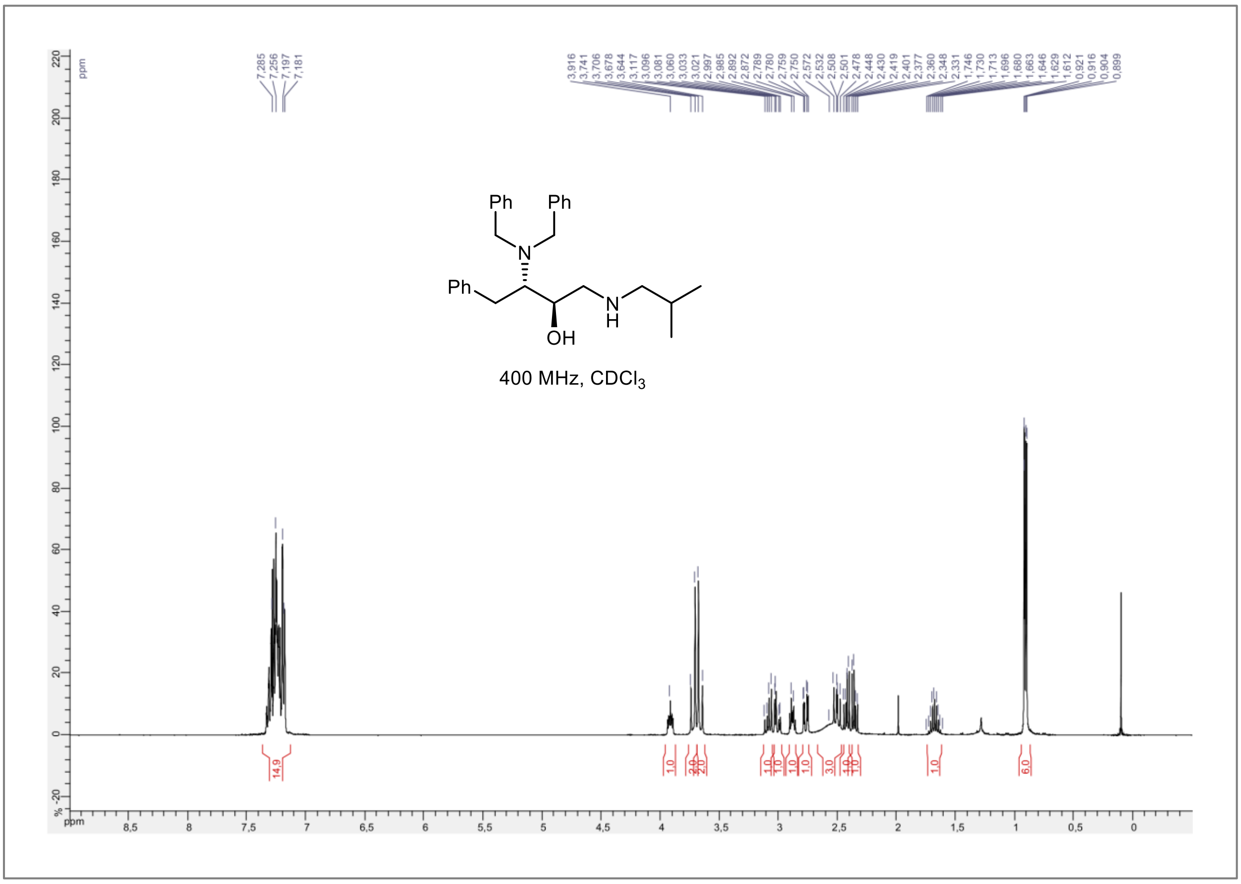

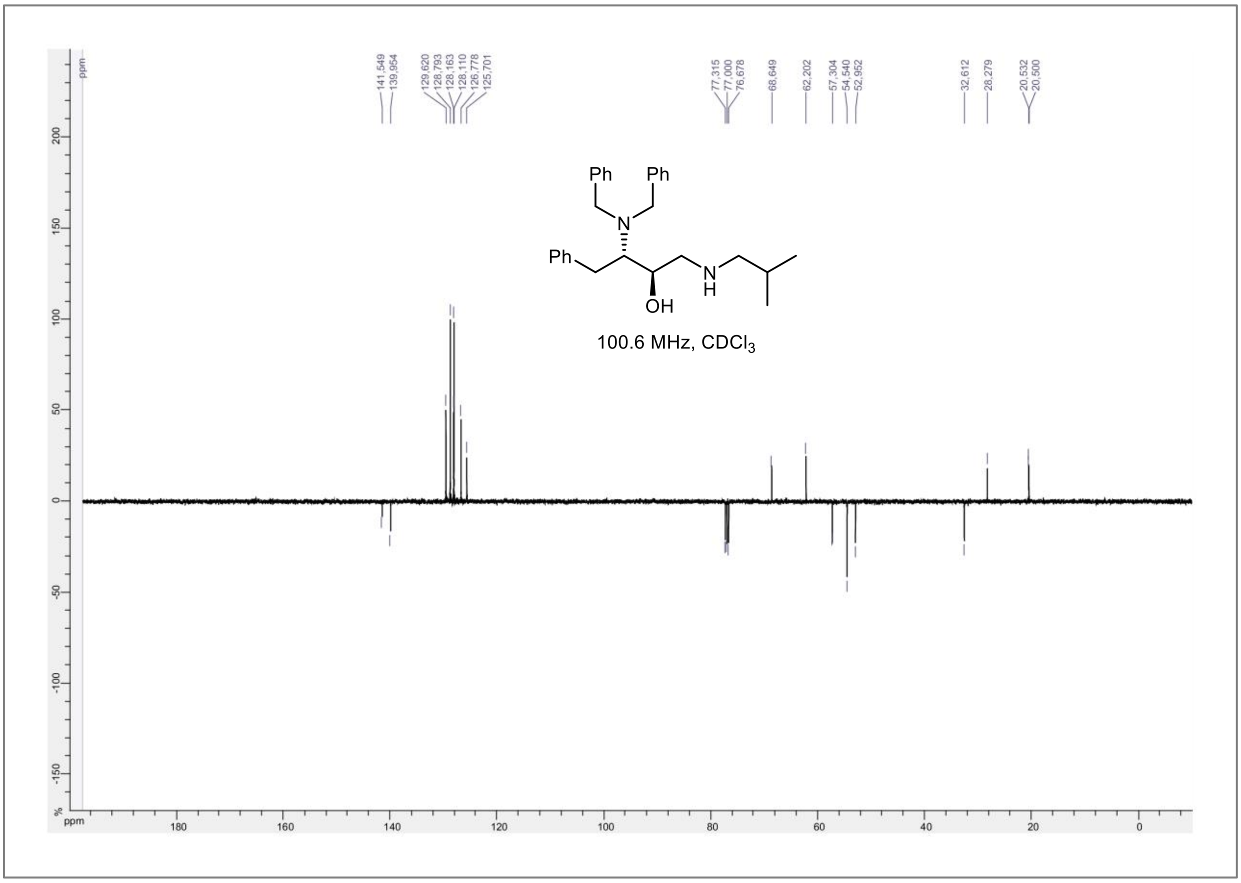

(2*R*,3*S*)-1-(Butylamino)-3-(dibenzylamino)-4-phenylbutan-2-ol (**3b**)

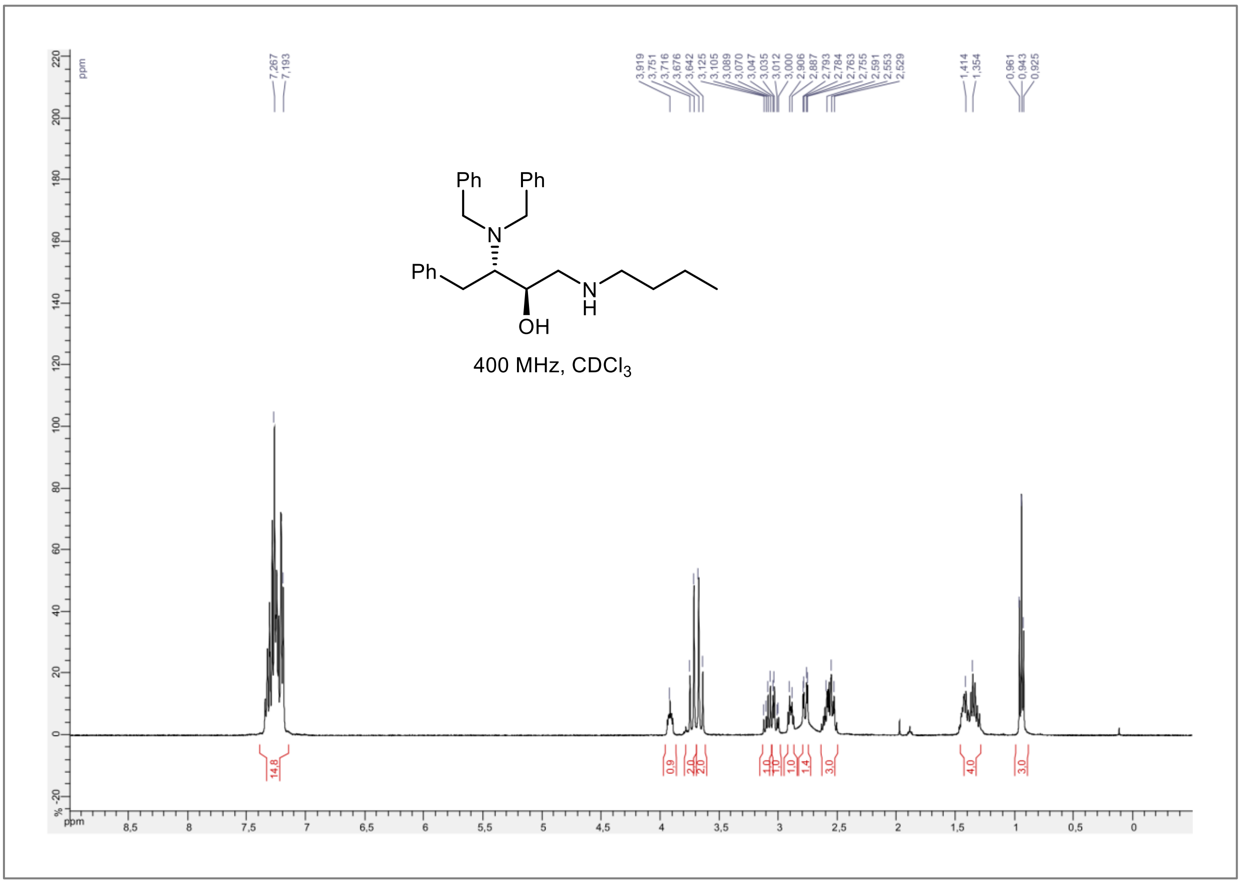

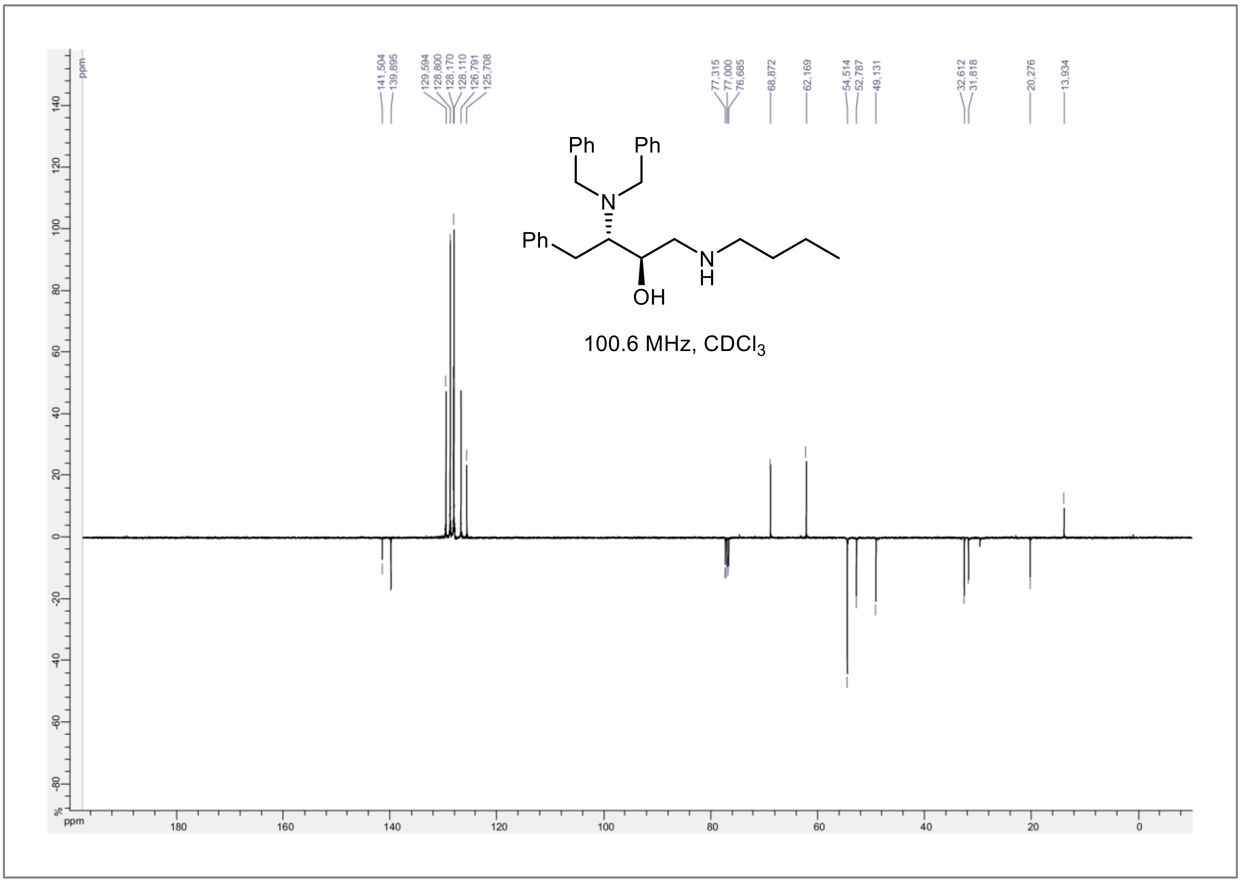

(2R,3S)-3-(Dibenzylamino)-4-phenyl-1-((3-phenylpropyl)amino)butan-2-ol (**3c**)

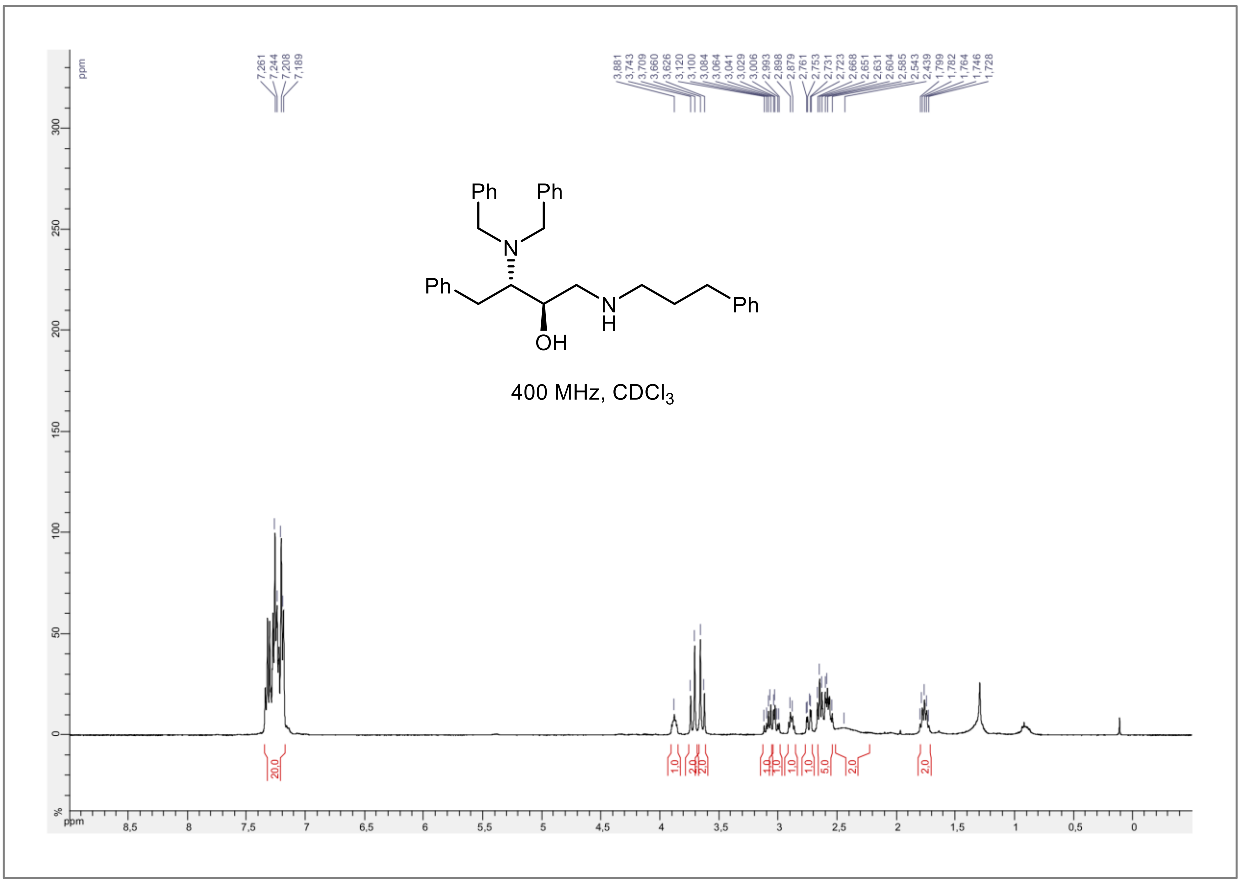

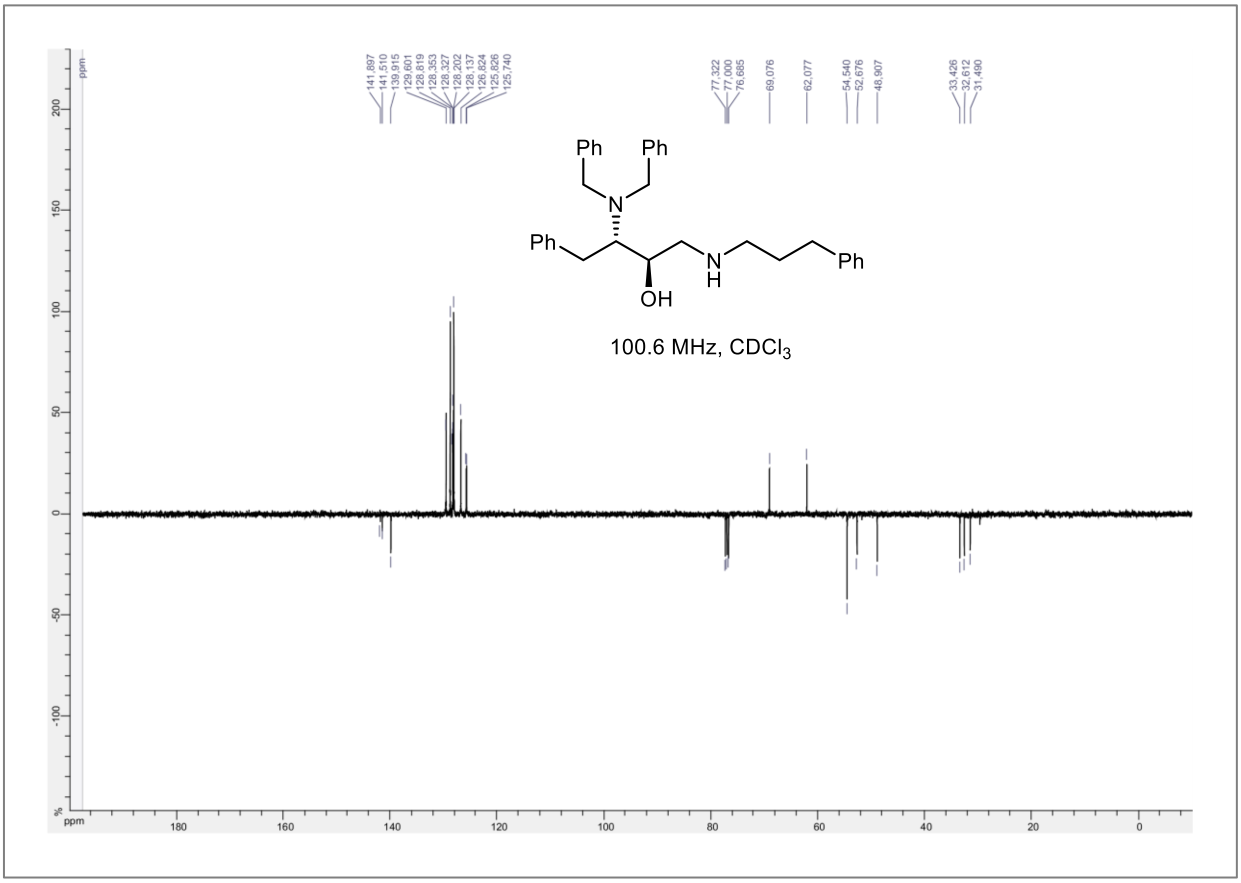

(2*R*,3*S*)-1-(Benzylamino)-3-(dibenzylamino)-4-phenylbutan-2-ol (**3d**)

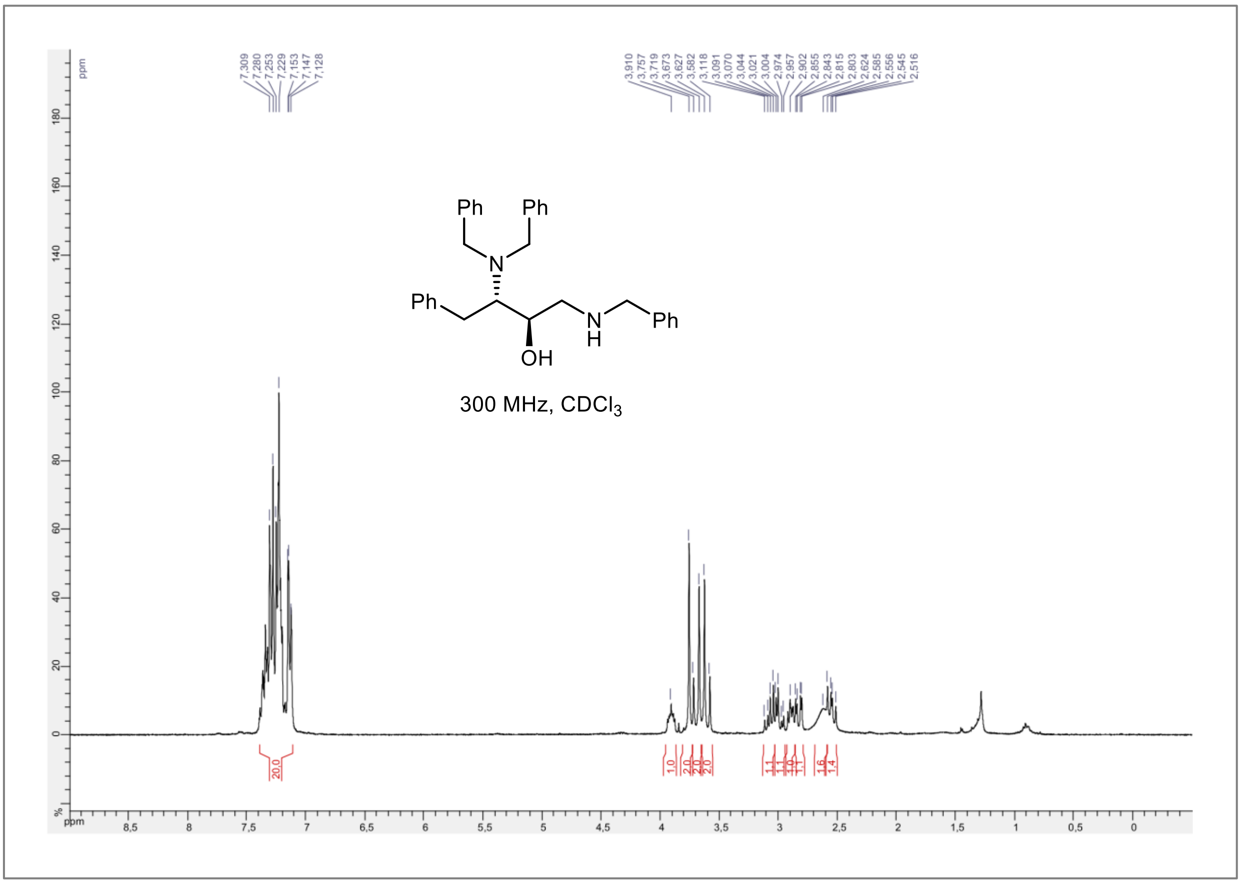

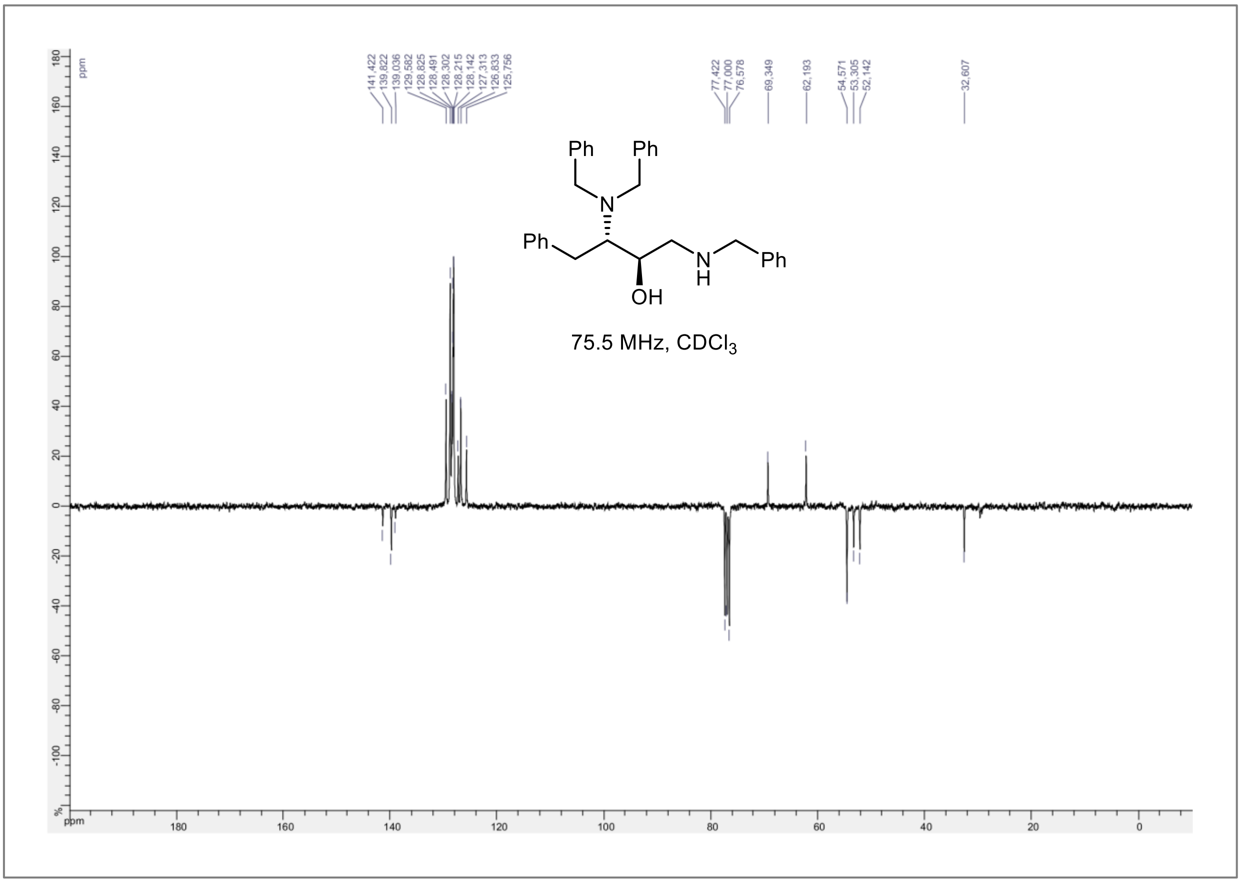

(2*R*,3*S*)-1-(Allylamino)-3-(dibenzylamino)-4-phenylbutan-2-ol (**3e**)

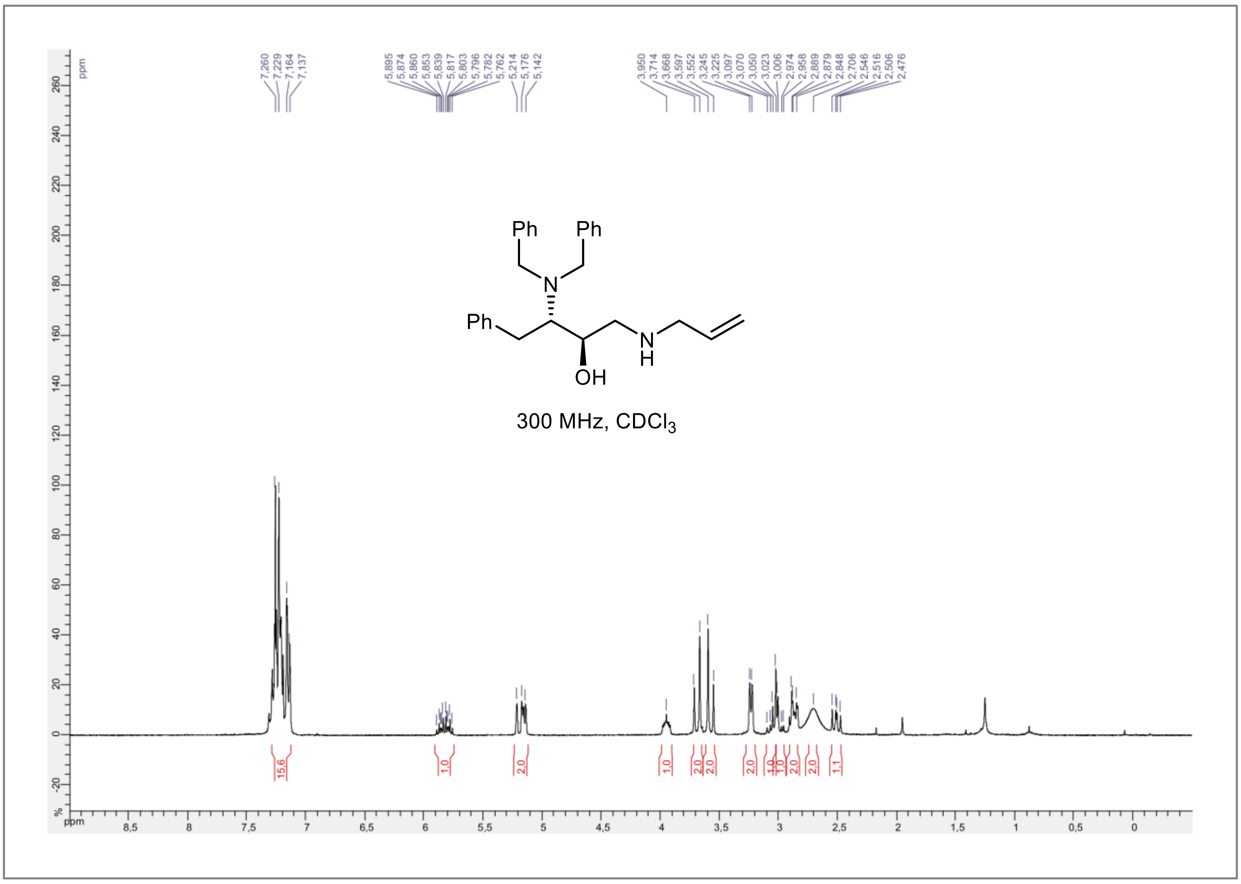

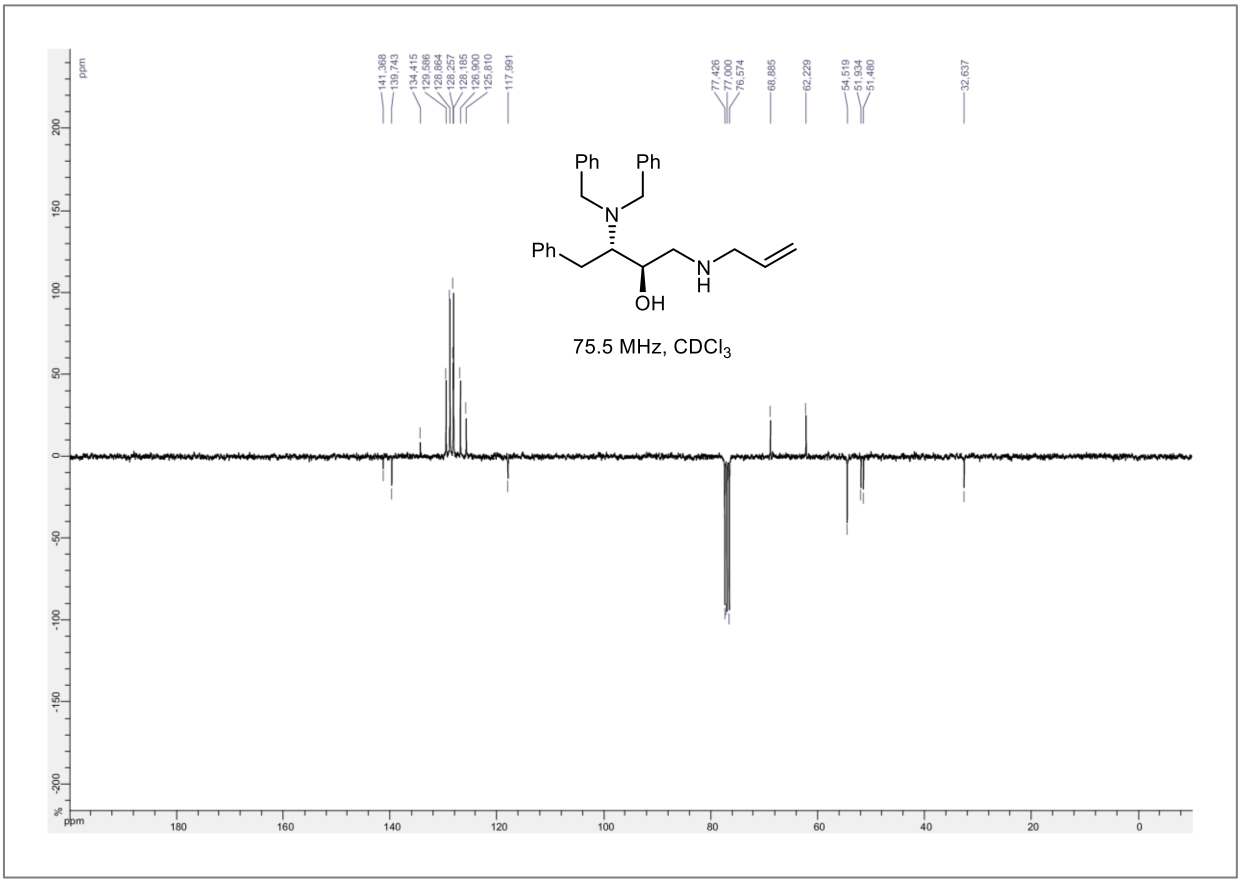

(2*R*,3*S*)-3-(Dibenzylamino)-4-phenyl-1-(prop-2-yn-1-ylamino)butan-2-ol (**3f**)

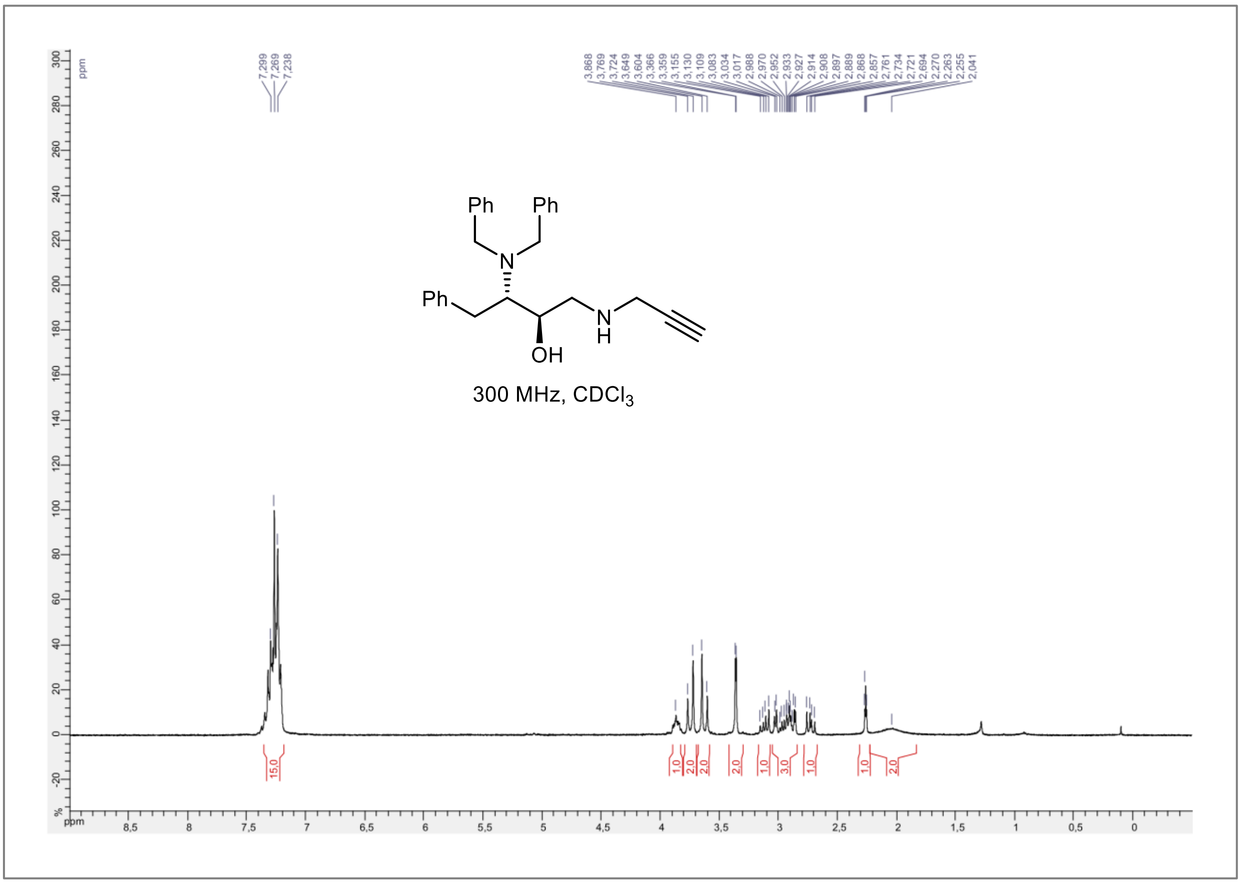

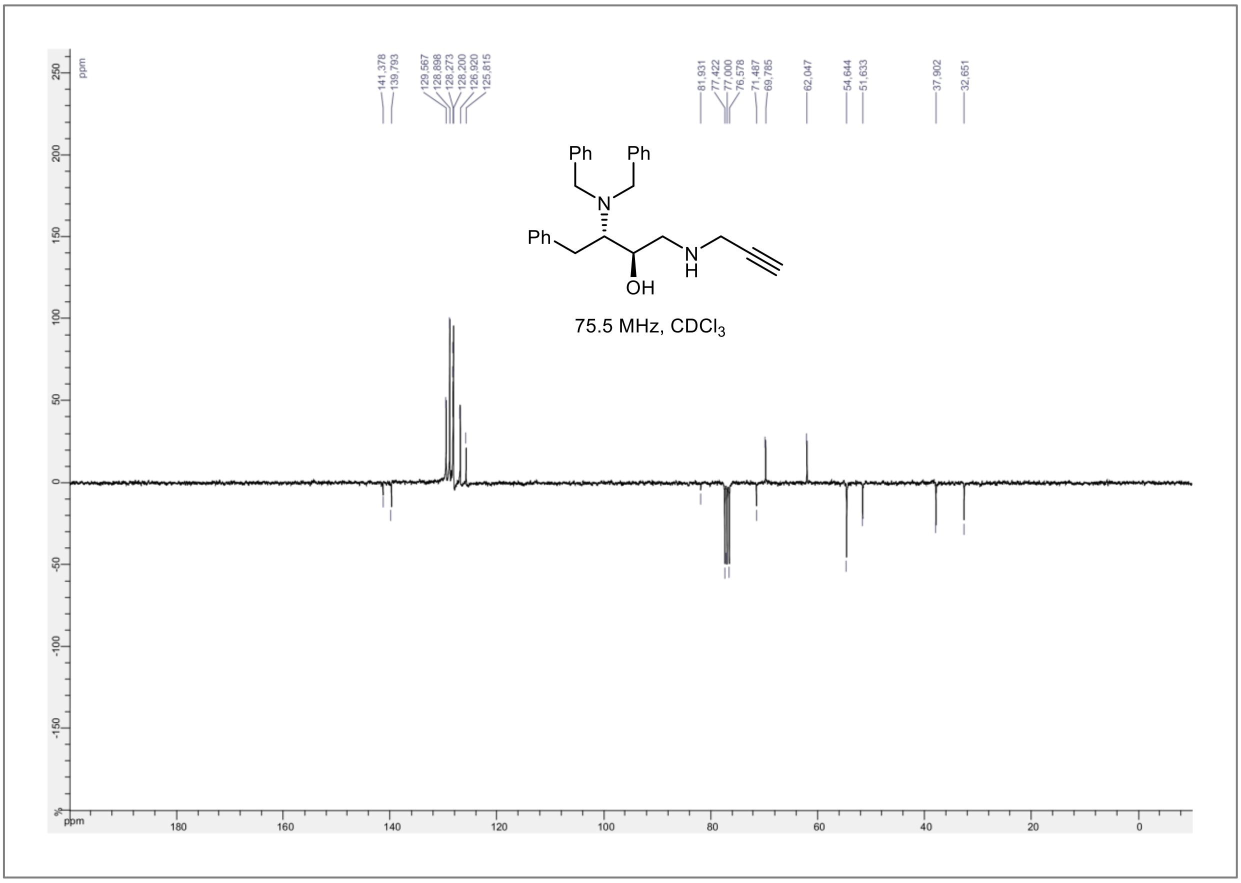

(2*R*,3*S*)-3-(Dibenzylamino)-1-(isopropylamino)-4-phenylbutan-2-ol (**3g**)

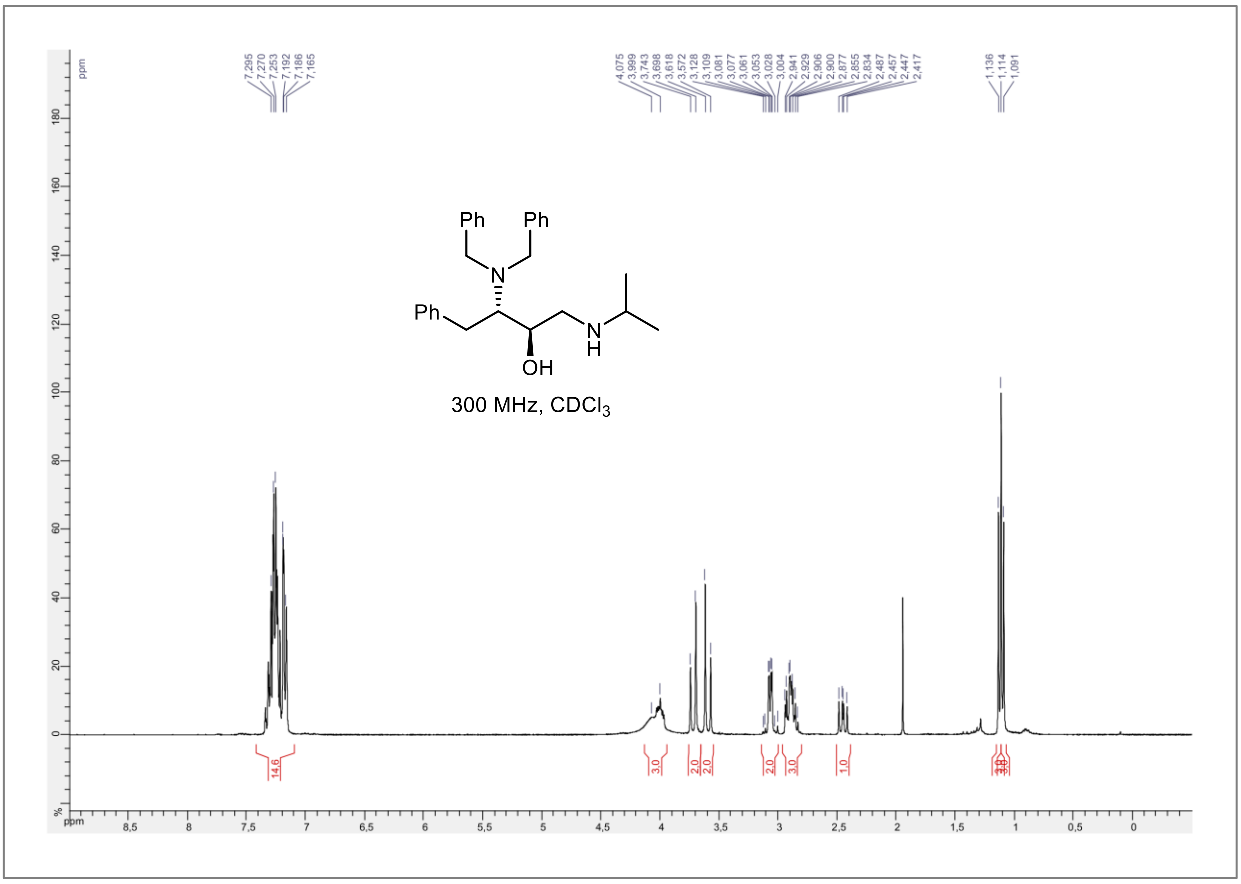

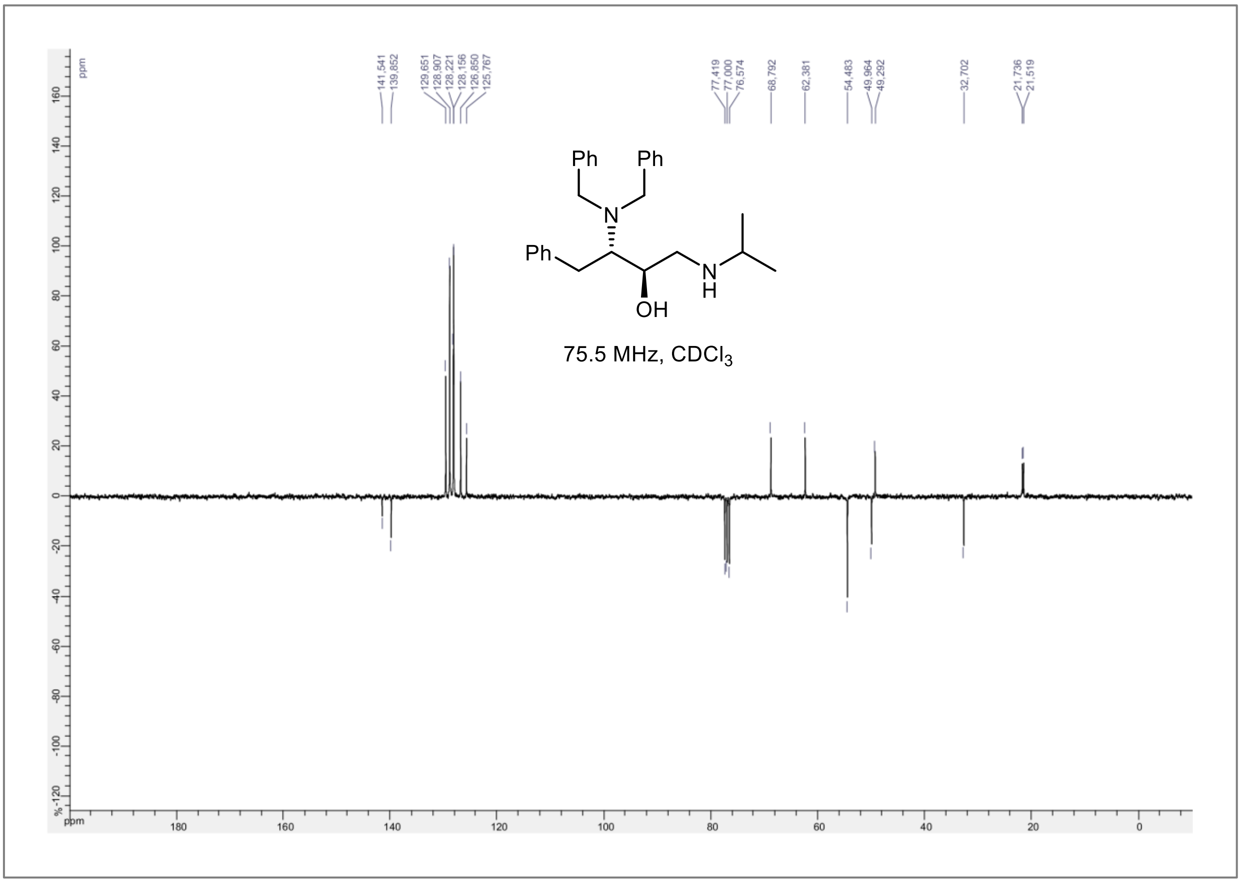

(2*R*,3*S*)-1-(Cyclopropylamino)-3-(dibenzylamino)-4-phenylbutan-2-ol (**3h**)

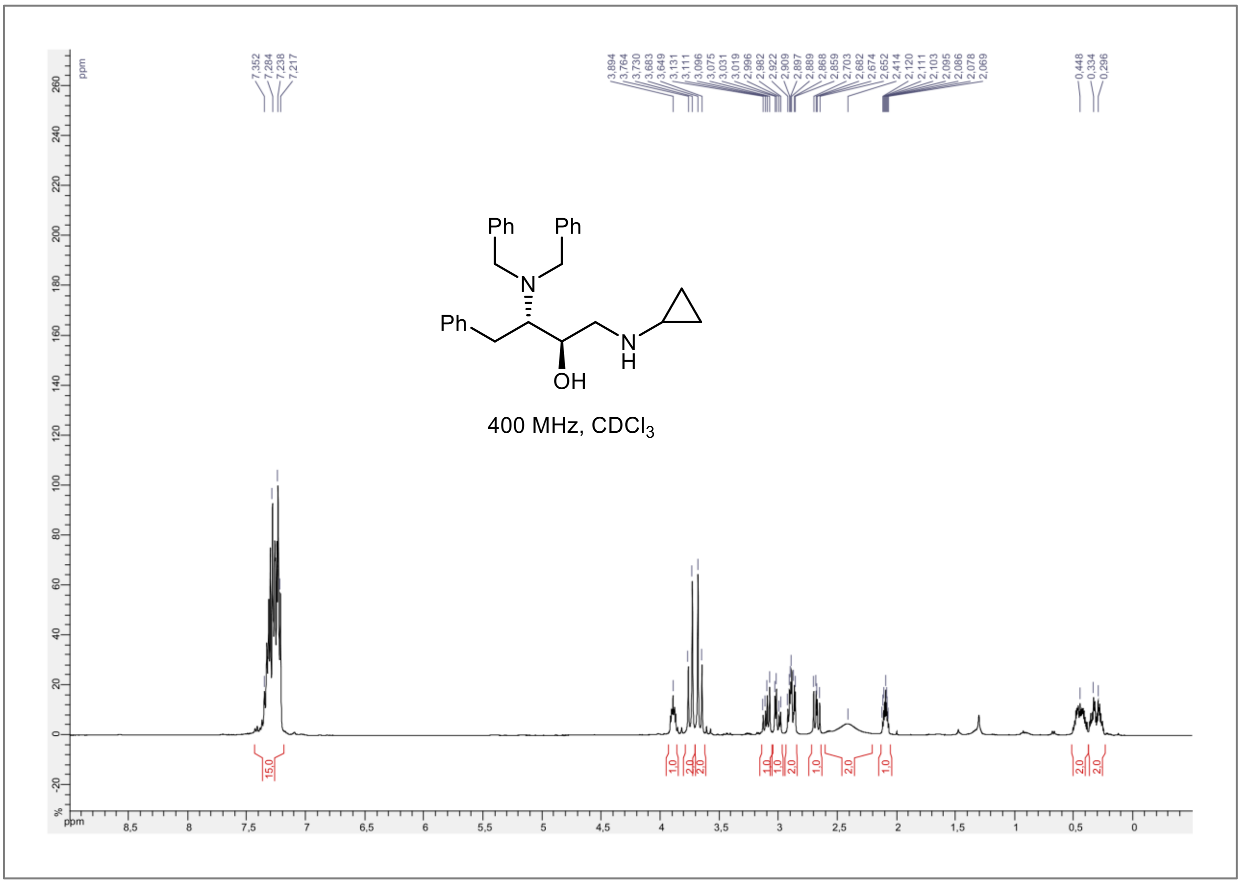

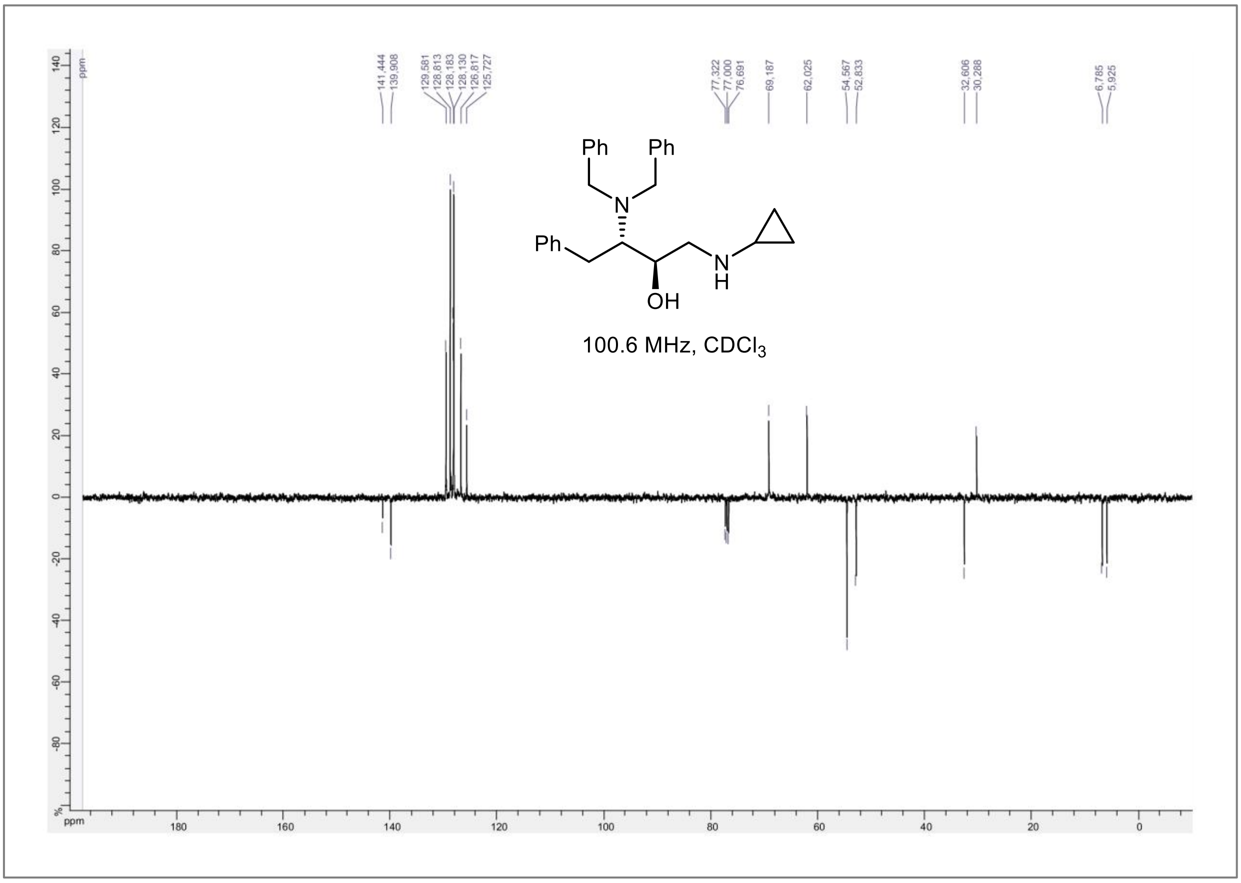

(2*R*,3*S*)-3-(Dibenzylamino)-1-morpholino-4-phenylbutan-2-ol (**3i**)

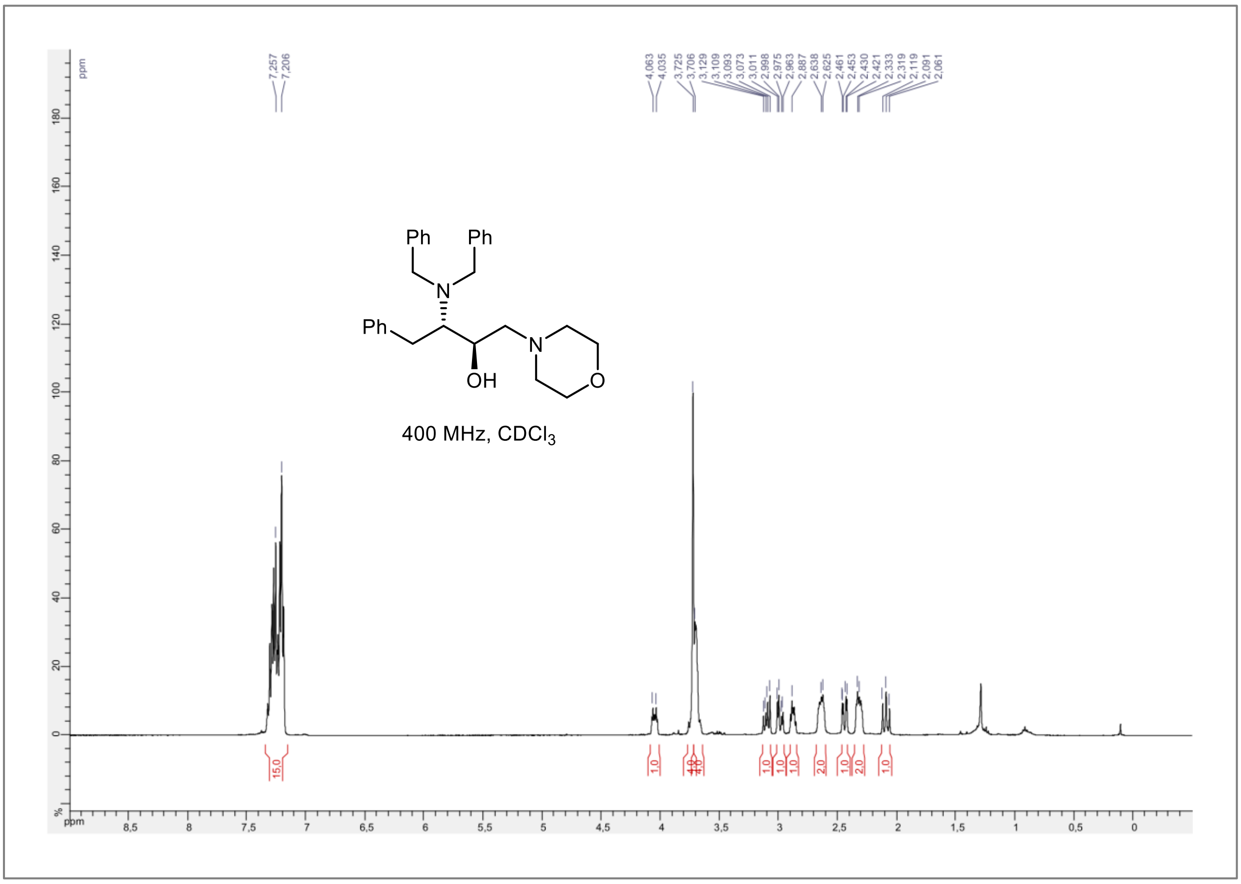

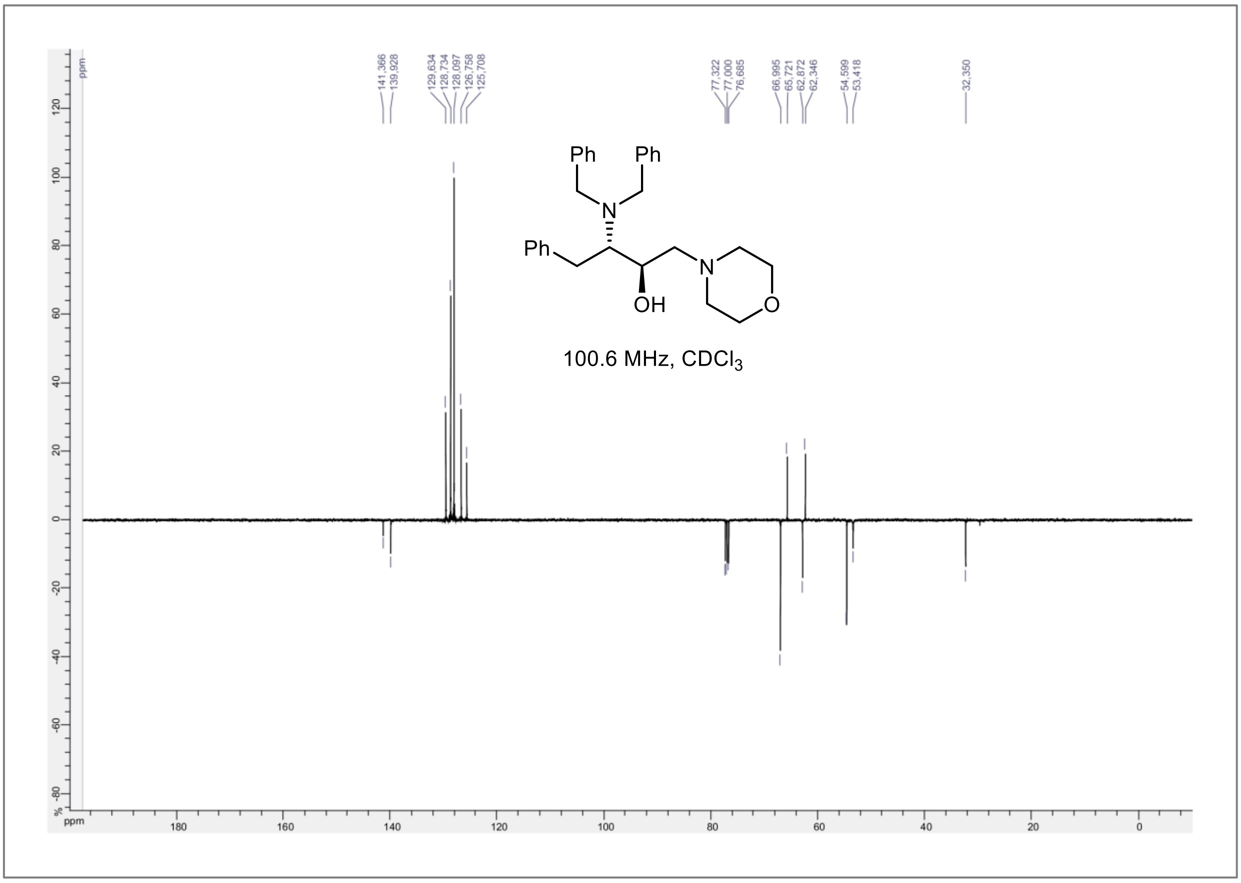

(2*R*,3*S*)-3-(Dibenzylamino)-4-phenyl-1-(pyrrolidin-1-yl)butan-2-ol (**3j**)

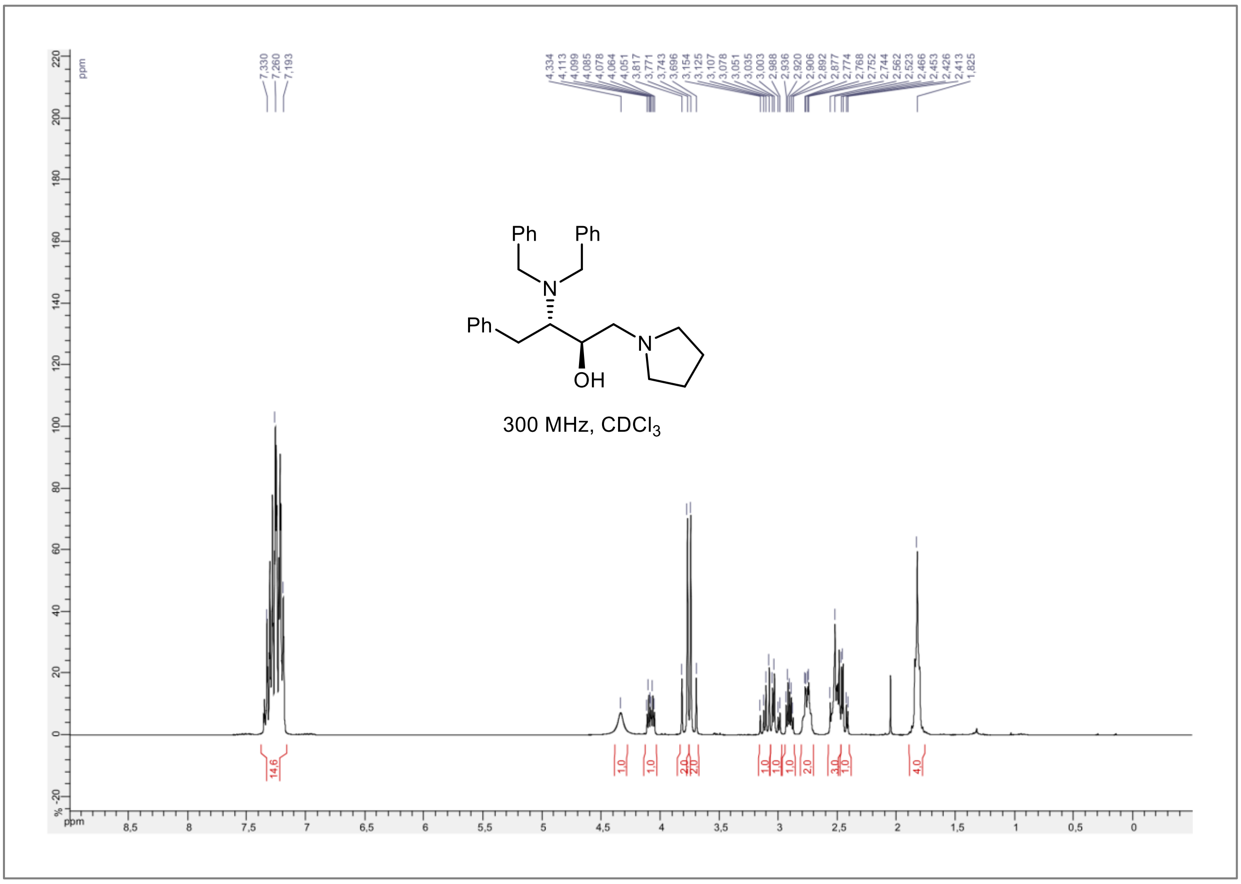

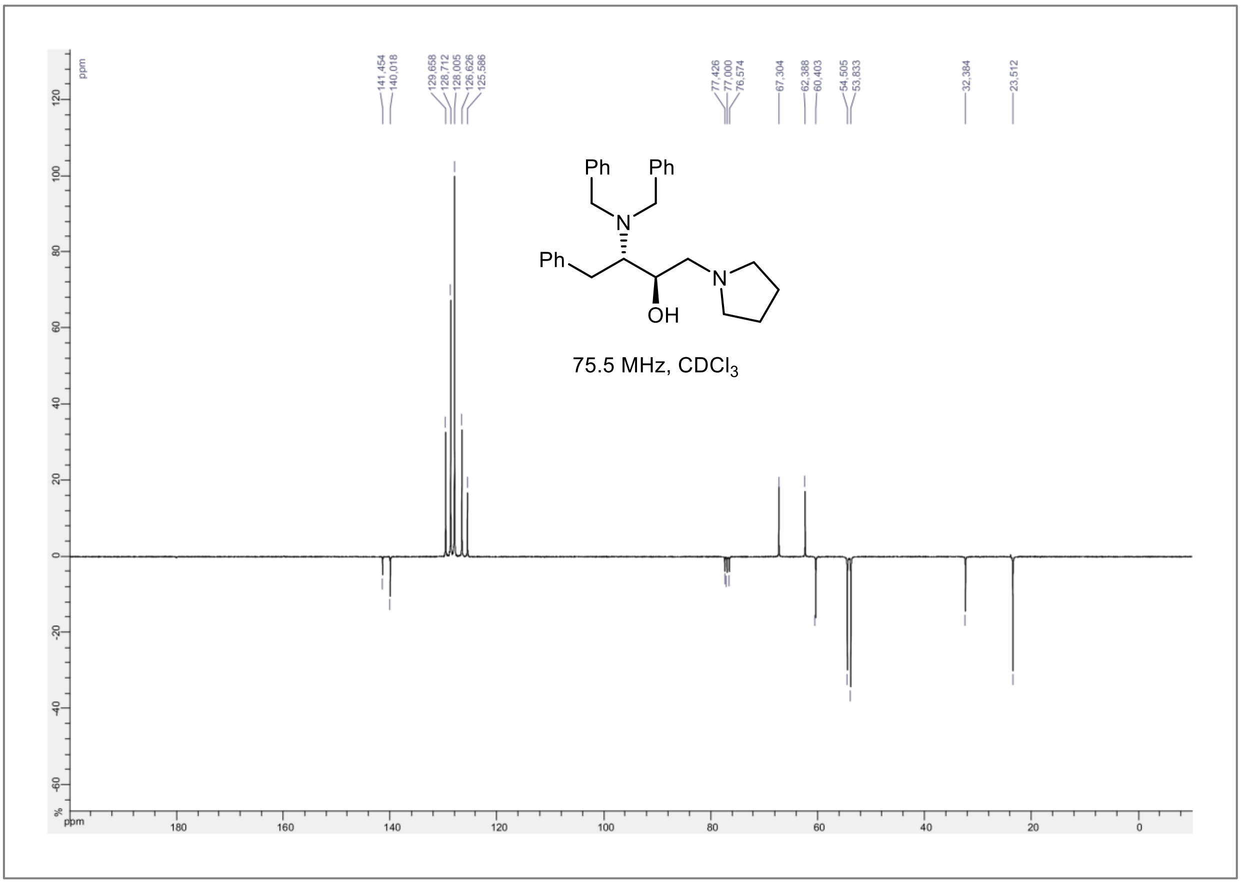

Crystallographic data for compound 1c

A single crystal suitable for X-ray diffraction was obtained by slow diffusion of pentane into a Et_2O solution of **1c** at ambient temperature.

X-ray diffraction data for compound **1c** were collected by using a VENTURE PHOTONIII c7 Bruker diffractometer with Micro-focus IμS source MoK_α radiation (λ = 0.71073 Å). The crystal was mounted on a CryoLoop (Hampton Research) with Paratone-N (Hampton Research) as cryoprotectant then flashfrozen in a nitrogen gas stream at 200 K. The temperature of the crystal was maintained at the selected value by means of a 700 series Cryostream cooling device to within an accuracy of ±1 K. The data were corrected for Lorentz polarization and absorption effects. The structure was solved by direct methods using SHELXS-97[1] and refined against F^2 by full-matrix least-squares techniques using SHELXL-2019[2] with anisotropic displacement parameters for all non-hydrogen atoms. Calculations were performed by using the Crystal Structure crystallographic software package WINGX.[3] The absolute configuration was determined for each compound by refining the Flack parameters[4] using a large number of Friedel's pairs.

An ORTEP plot and crystal data collection and refinement parameters are presented below.

CCDC 2373148 contains the supplementary crystallographic data for this paper. These data can be obtained free of charge from the Cambridge Crystallographic Data Centre and Fachinformationszentrum Karlsruhe via http://www.ccdc.cam.ac.uk/structures/.

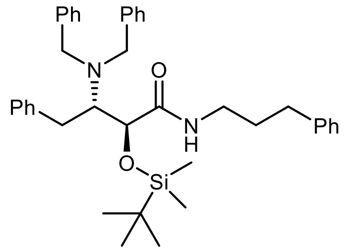

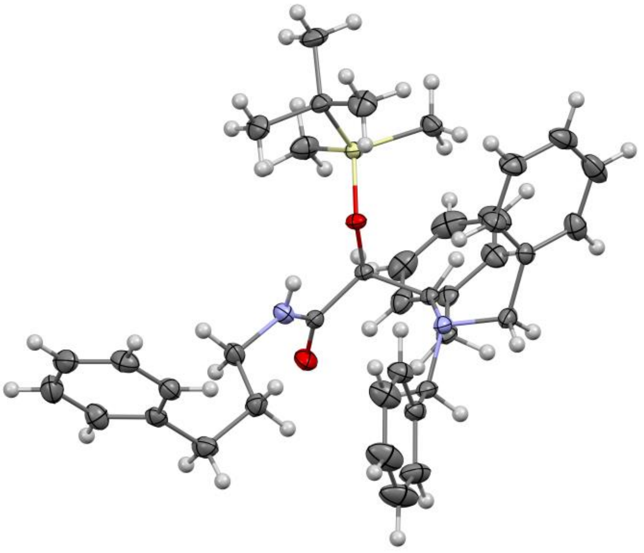

ORTEP drawing of compound **1c**. Thermal ellipsoids are shown at the 30% level.

**Compound**	**1c**
CCDC	2373148
Empirical Formula	$C_{39} H_{50} N_2 O_2 Si$
M_r	606.90
Crystal size, mm^3	0.13 × 0.09 × 0.03
Crystal system	orthorhombic
Space group	$P\ 2_1 2_1 2_1$
a, Å	10.2140(2)
b, Å	16.5390(4)
c, Å	20.9406(6)
α, °	90
β, °	90
γ, °	90
Cell volume, $Å^3$	3537.48(15)
Z ; Z'	4 ; 1
T, K	200(1)
Radiation type ; wavelength Å	MoKα ; 0.71073
F_{000}	1312
μ, mm^{-1}	0.101
range, °	2.218 - 31.005
Reflection collected	197 208
Reflections unique	11 271
R_{int}	0.0652
GOF	1.017
Refl. obs. (I>2(I))	9 274
Parameters ; restraints	402 ; 0
wR_2 (all data)	0.1208
R value (I>2(I))	0.0439
Flack parameter	0.00(2)
Largest diff. peak and hole ($e^-.Å^{-3}$)	0.318 ; –0.141

1. Sheldrick, G. M. SHELXS-97, Program for Crystal Structure Solution, University of Göttingen, Göttingen, Germany, **1997**.
2. Sheldrick, G. M. *Acta Crystallogr. A* **2008**, *64*, 112–122.
3. Farrugia, L. J. *J. Appl. Cryst*. **1999**, *32*, 837–838.
4. Parsons, S.; Flack, H. D.; Wagner, T. *Acta Crystallogr. B* **2013**, *69*, 249–259.
